# Supplementary material for: COVseq is a cost-effective workflow for mass-scale SARS-CoV-2 genomic surveillance
Source: Nat Commun. 2021 Jun 23;12:3903. doi: 10.1038/s41467-021-24078-9 (PMC8222401; doi:10.1038/s41467-021-24078-9)
Supplement: Supplementary file 2 — Supplementary Information [file 41467_2021_24078_MOESM2_ESM.pdf]

SUPPLEMENTARY INFORMATION

COVseq is a cost-effective workflow for mass-scale SARS-CoV-2 genomic surveillance

Michele Simonetti, Ning Zhang, Luuk Harbers, Maria Grazia Milia, Silvia Brossa, Thi Thu Huong Nguyen, Francesco Cerutti, Enrico Berrino, Anna Sapino, Magda Bienko, Antonino Sottile, Valeria Ghisetti & Nicola Crosetto

1. Supplementary Figures pg. 2
2. Supplementary Methods pg. 11
3. Supplementary Tables pg. 21
4. Supplementary Notes pg. 22

1. Supplementary Figures

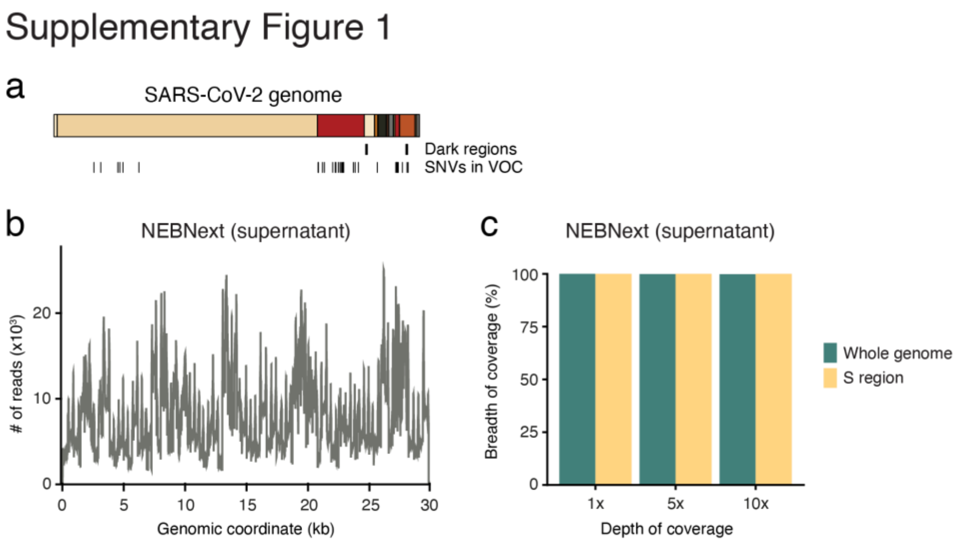

Supplementary Fig. 1. Implementation of the Centers for Disease Control and Prevention (CDC) multiplexed PCR assay for SARS-CoV-2 whole genome amplification (see scheme in **Fig. 1a**). (**a**) Location along the Severe Acute Respiratory Syndrome Coronavirus-2 (SARS-CoV-2) genome (top) of so-called dark regions that, in silico, are not covered by SE300 COVseq (vertical black bars, middle). The locations of the single-nucleotide variants (SNVs) from the UK, South African and Brazilian variants of concern (VOC) are shown in the bottom (vertical black bars). (**b**) Depth of coverage (SE75) along the SARS-CoV-2 genome for a single library prepared using the NEBNext library preparation kit on RNA extracted from the supernatant of a SARS-CoV-2 viral culture (see **Supplementary Data 4 and Methods** for details). The genomic regions displaying the highest depth of coverage correspond to the regions covered by more amplicons in **Fig. 1a**. (**c**) Breadth of coverage (SE75) of the whole SARS-CoV-2 genome and S region at various sequencing depths, for the same NEBNext library as shown in (a).

Supplementary Figure 2

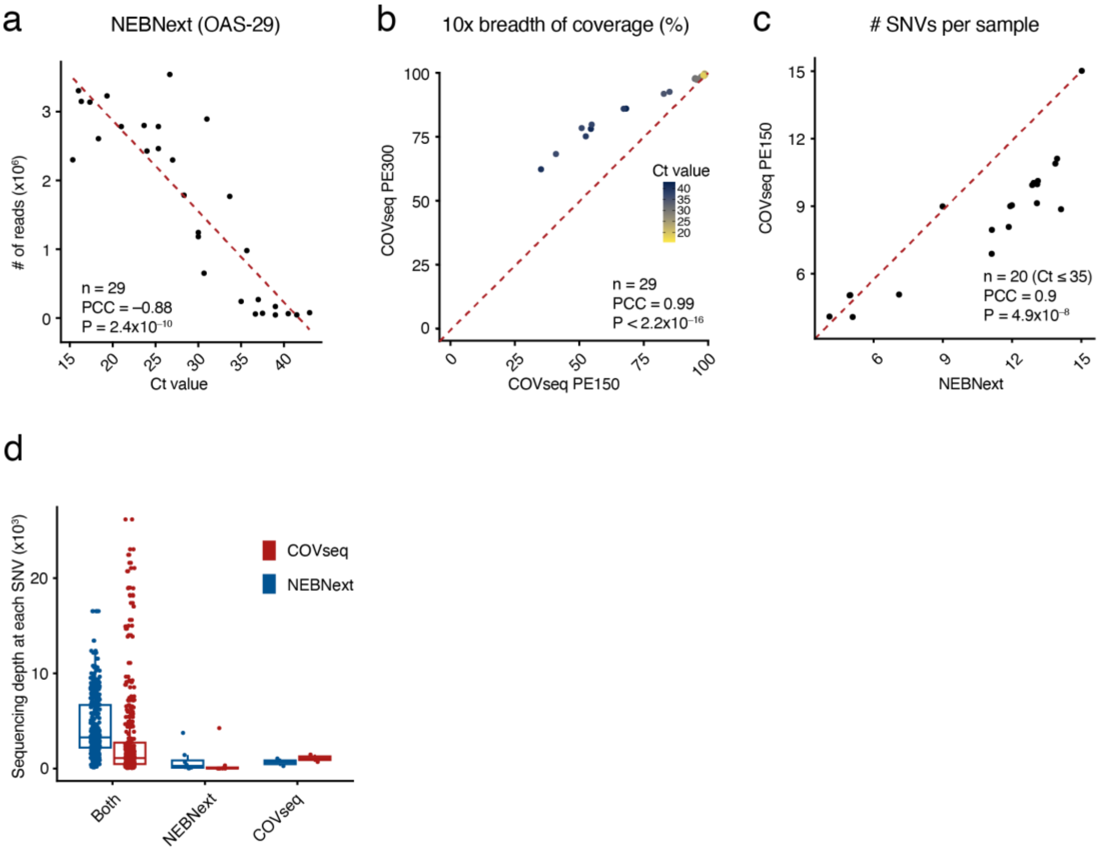

Supplementary Fig. 2. COVseq benchmarking. (**a**) Correlation between the total number of reads obtained and the corresponding Ct value for the OAS-29 samples processed by NEBNext (SE75 sequencing on NextSeq 500). (**b**) Correlation between the breadth of coverage for the same samples as in (a), sequenced by PE150 on NextSeq 500 or PE300 on MiSeq. The color of each point indicates the Ct value of the corresponding sample. Red dashed line indicates the bisector line. (**c**) Correlation between the number of single-nucleotide variants (SNVs) per sample detected by COVseq *vs*. NEBNext in 20 (*n*) out of the 29 OAS-29 samples with Ct ≤ 35. Red dashed line indicates the bisector line. (**d**) Number of reads covering each of the SNVs identified by COVseq, NEBNext or both in the OAS-29 samples. Each dot represents one SNV. In the boxplots, each box extends from the 25th to the 75th percentile, the midline represents the median, and the whiskers extend from –1.5×IQR to +1.5×IQR from the closest quartile, where IQR is the inter-quartile range. In (a-c): n, number of samples; PCC, Pearson's correlation coefficient; *P*, t-test, two-tailed. For a description of the samples, see **Supplementary Data 4**.

Supplementary Figure 3

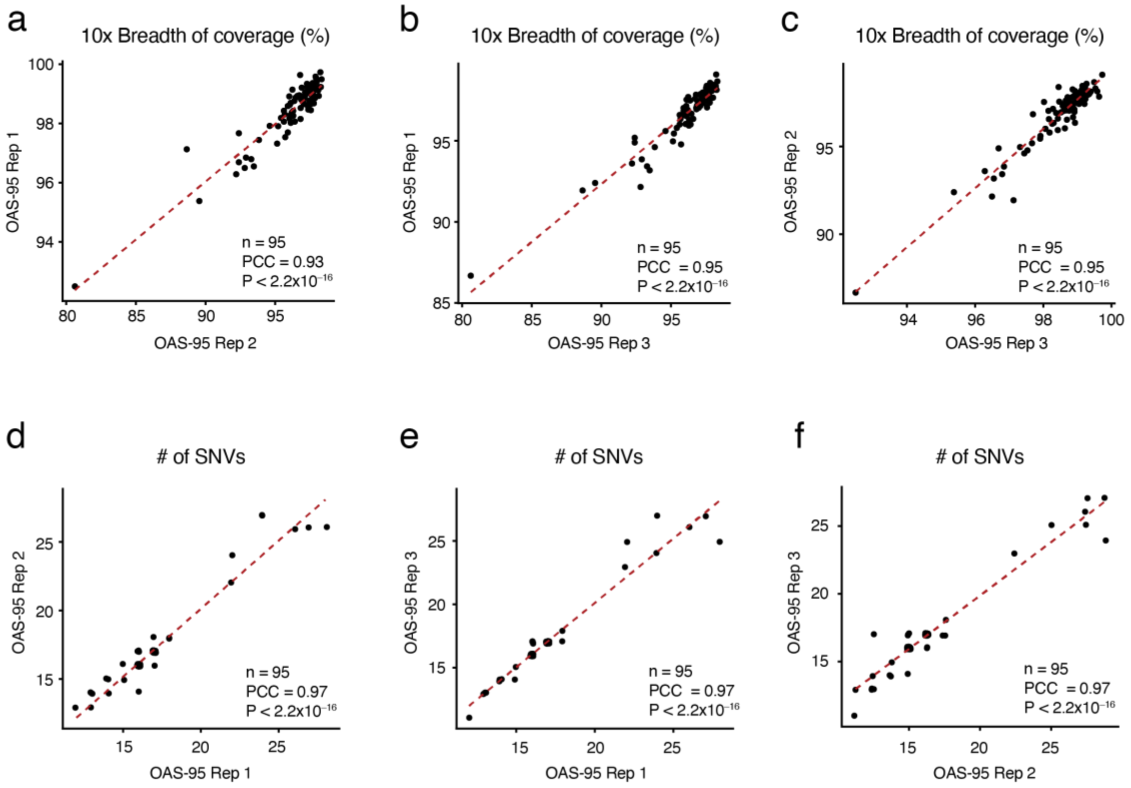

Supplementary Fig. 3. COVseq reproducibility between OAS-95 samples. (**a-c**) Correlation between the breadth of coverage at 10× sequencing depth between three replicate (Rep) COVseq libraries prepared from 95 OAS-95 samples. (**d-f**) Correlation between the number of single-nucleotide variants (SNVs) per sample detected in the same COVseq replicates shown in (a-c). In all panels: *n*, number of samples; PCC, Pearson's correlation coefficient; *P*, t-test, two-tailed. For a description of the samples, see **Supplementary Data 4**.

Supplementary Figure 4

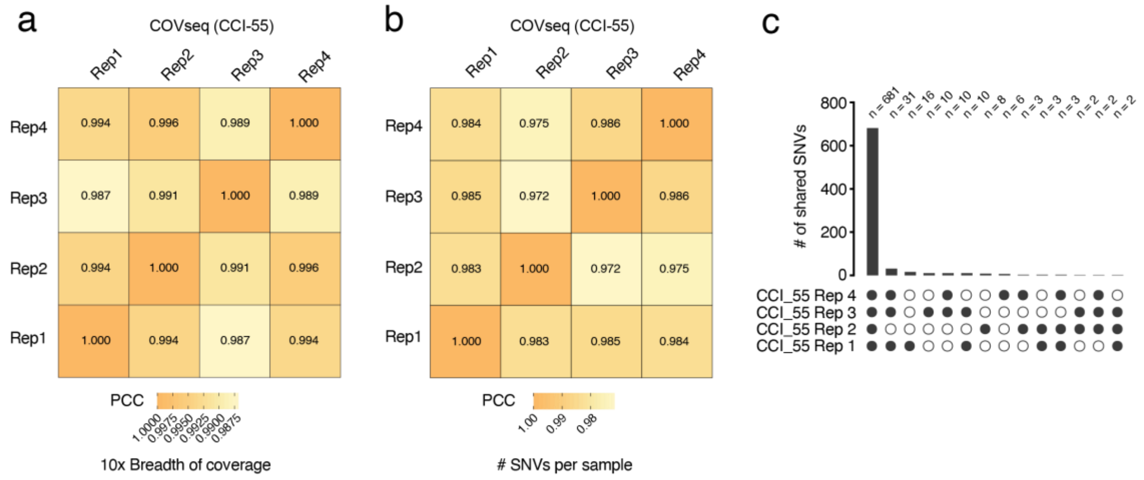

Supplementary Fig. 4. COVseq reproducibility between 55 CCI-55 samples. (**a**) Pearson's correlation coefficient (PCC) of the breadth of coverage at 10× sequencing depth between the four replicate (Rep) COVseq libraries prepared from 55 CCI-55 samples. (**b**) Same as in (a) but showing the PCC of the number of single-nucleotide variants (SNVs) in each replicate. (**c**) Bar plot showing the number of shared SNVs between the four COVseq replicates shown in (a-b). *n*, number of SNVs shared between one, two, three or four replicates (filled circles). For a description of the samples, see **Supplementary Data 4**.

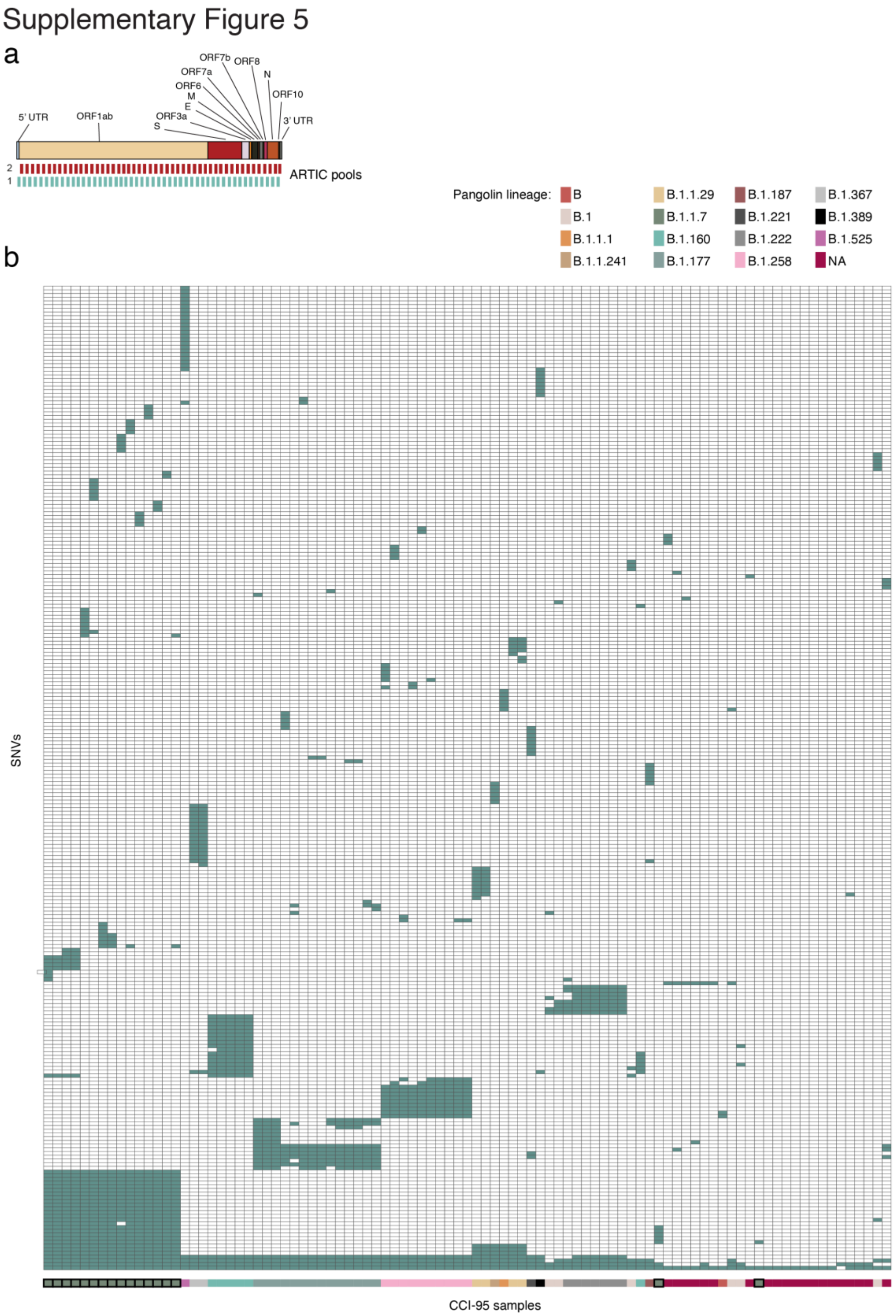

Supplementary Fig. 5. Modified COVseq workflow based on the multiplexed PCR strategy developed by the ARTIC network (https://artic.network/), which further reduces the turnaround

time and costs (see **Methods and Supplementary Notes**). (**a**) Location along the SARS-CoV-2 genome (top) of the ARTIC V3 amplicon pools (colored rectangles). Gene names (top) are according to the reference SARS-CoV-2 sequence NC_045512.2. (**b**) Examples of Pangolin lineages and single-nucleotide variants (SNVs) identified in 95 samples (CCI-95, see **Supplementary Data 4**) sequenced in the frame of the ongoing SARS-CoV-2 genomic surveillance for the Piemonte Region in North-West Italy, in which we are testing the ARTIC-COVseq workflow shown in (a). Rows indicate all the detected SNVs, columns indicate the samples. The Pangolin lineage assigned to the samples encircled in black was confirmed by sequencing the same samples using a commercial kit (CleanPlex) to prepare the libraries (see **Supplementary Notes**).

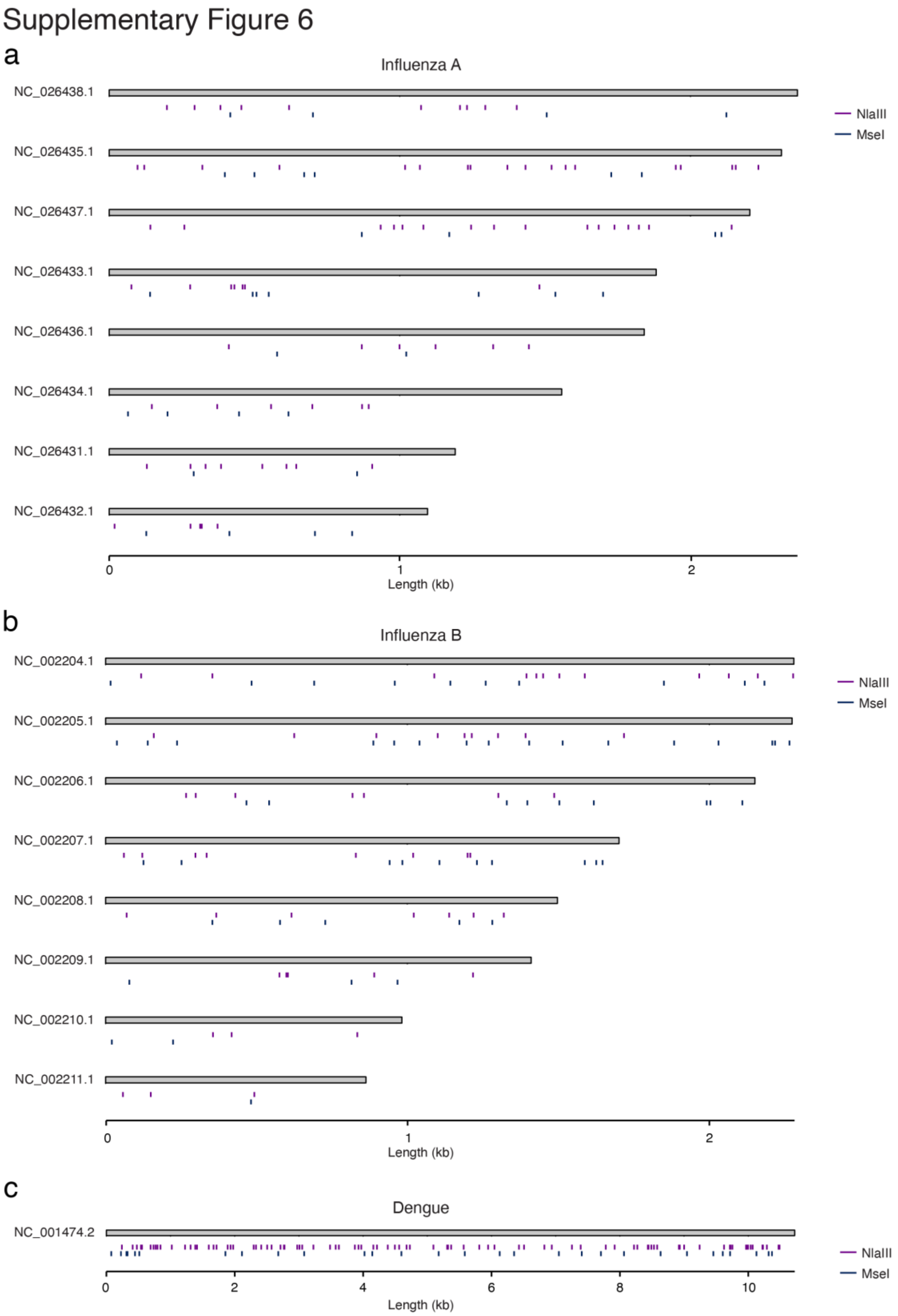

Supplementary Fig. 6. Applicability of COVseq beyond SARS-CoV-2. (**a-c**) Schemes of the locations of MseI and NlaIII recognition sites (vertical-colored bars) along the genome of the H1N1 strain of Influenza type A (a), Influenza type B (b) and Dengue virus (c). Reference

NCBI accession numbers are indicated near each scheme. Combining these two enzymes with virus-specific multiplexed PCR assays (such as those listed in **Supplementary Table 1**) would expand the applications of COVseq in genomic epidemiological surveillance. In all the panels: kb, kilobase.

Supplementary Figure 7

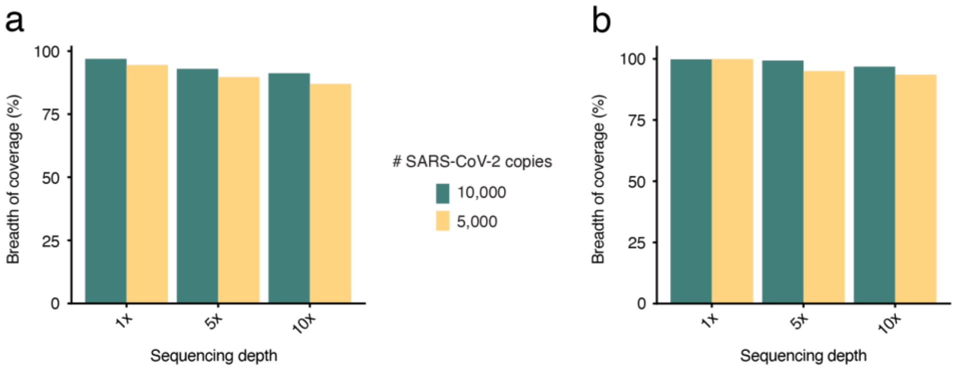

Supplementary Fig. 7. Proof-of-principle COVseq experiment performing all the steps until in vitro transcription (IVT) in small volumes using the I-DOT nanodispensing system (see **Supplementary Methods**). (**a**) Breadth of coverage of the SARS-CoV-2 reference genome at varying sequencing depths, for two different input amounts of synthetic SARS-CoV-2 RNA (5,000 and 10,000 copies). (**b**) Same as in (a), but for the S gene.

2. Supplementary Methods

1. Step-by-step COVseq protocol

REAGENTS

- Absolute Ethanol (VWR, cat. no. 20816.367)
- Nuclease-Free Water (Thermo Fisher Scientific, cat. no. AM9932)
- Mineral oil (Sigma, cat. no. M5904)
- Random hexamers (50 µM) (Thermo Fisher Scientific, cat. no. N80800127)
- dNTPs (10 mM) (Thermo Fisher Scientific, cat.no. R0191)
- NEBNext Q5 Hot Start HiFi PCR Master Mix (NEB, cat.no. M0543L)
- SYBR Green (Thermo Fisher Scientific, cat. no. S7563)
- Primer pools 1,2,3,4,5 and 6 (IDT custom at 50 µM) (see **Supplementary Data 1**)
- IDT ARTIC nCoV-2019 V3 panel pools 100 µM (IDT, cat. no. 10006788)
- MseI (NEB, cat.no. R0525L)
- NlaIII (NEB, cat.no. R0125L)
- CutSmart buffer (NEB, cat. no. B7204S)
- T4 DNA Ligase (Thermo Fisher Scientific, cat. no. EL0011)
- T4 DNA rapid Ligase (Thermo Fisher Scientific, cat. no. K1423)
- COVseq oligonucleotide adapters (see **Supplementary Data 2**)
- UltraPure BSA (50 mg/ml) (Thermo Fisher Scientific, cat. no. AM2616)
- ATP Solution (100 mM) (Thermo Fisher Scientific, cat. no. R0441)
- dNTPs (25mM) (Thermo Fisher Scientific, cat.no. R1121)
- MEGAscript T7 Transcription Kit (Thermo Fisher Scientific, cat. no. AM1334)
- DNase I, RNase-free (Thermo Fisher Scientific, cat. no. AM2222)
- RA3 adapter and RTP, RP1 and RPI primers (custom-made by Integrated DNA Technologies Inc. based on the sequences in the TruSeq Small RNA Library Preparation kit, Illumina)
- RNaseOUT Recombinant Ribonuclease Inhibitor (Invitrogen, cat. no. 10777-019)
- T4 RNA ligase 2, truncated (NEB, cat. no. M0242L)
- SuperScript IV Reverse Transcriptase (Thermo Fisher Scientific, cat. no. 18090050)
- NEBNext UltraII Q5 PCR Mastermix (NEB, cat. no. M0544S)
- Agencourt RNAClean XP with Scalable throughput (Beckman Coulter, cat. no. A63987)
- Agencourt AMPure XP (Beckman Coulter, cat. no. A63881)
- Qubit RNA BR Assay Kit (Thermo Fisher Scientific, cat. no. Q10211)

- Qubit dsDNA BR Assay Kit (Thermo Fisher Scientific, cat. no. Q32850)
- Qubit dsDNA HS Assay Kit (Thermo Fisher Scientific, cat. no. Q32851)
- Bioanalyzer High Sensitivity DNA Kit (Agilent, cat. no. 5067-4627)
- Optional: RNase H (Thermo Fisher Scientific, cat.no. 18021014)

CONSUMABLES

- Eppendorf DNA LoBind microcentrifuge tubes 0.5 ml (Sigma, cat. no. EP0030108035-250EA)
- Eppendorf DNA LoBind microcentrifuge tubes 1.5 ml (Sigma, cat. no. EP0030108051-250EA)
- Sapphire Filter tips, low retention (Greiner Bio-One, cat. no. 771265, 773265, 738265, 750265)
- microTUBE-50 AFA Fiber Screw-Cap (25) (Covaris, cat. no. 520166)
- 96-well plates (Thermo Fisher Scientific, cat. no. 4316813)
- 384-well plates (Thermo Fisher Scientific, cat. no. 4483320)
- Qubit Assay Tubes (Thermo Fisher Scientific, cat. no. Q32856)
- Bioanalyzer High-sensitivity DNA kit (Chips) (Agilent, cat. no. 5067-4626)

EQUIPMENT

- Incubator (for example, Binder incubator, Model KB 53 or Boekel Scientific InSlide Out, cat. no. 05-450-50)
- Tabletop centrifuge (for example, Eppendorf Microcentrifuge 5424)
- I-DOT One (Dispendix GmbH, Stuttgart, Germany)
- Thermoshaker (for example, Eppendorf Thermomixer Compact)
- PCR thermocycler (for example, Biometra TRIO)
- Sonication device (for example, ME220 Focused-ultrasonicator, Covaris)
- DynaMag-2 Magnet (Thermo Fisher Scientific, cat. no. 12321D)
- Qubit 2.0 Fluorometer (Thermo Fisher Scientific, cat. no. Q32866)
- Bioanalyzer 2100 (Agilent, cat. no. G2943CA)

PROCEDURE

DAY 1

First strand synthesis

1. Mix the following components:

RNA	5 µL
50 µM random hexamers	1 µL
10 mM dNTPs	1 µL
Nuclease-Free Water	6 µL

2. Incubate for 5 min at 65 °C
3. Place the tube immediately on ice for 5 min
4. Add the following components:

5x SSIV buffer	4 µL
0.1 M DTT	1 µL
RNaseOUT	1 µL
SSIV Reverse Transcriptase enzyme	1 µL

5. Perform the following steps in a PCR thermocycler with the lid set to 85 °C:

1. 23 °C	10 min
2. 50 °C	10 min
3. 85 °C	10 min
4. 4 °C	Hold

6. Add 1 µL RNase H to the tube and incubate for 20 min at 37 °C
7. **Note**: RNase H is an optional step

Option 1: Multiplex PCR using the CDC primer pools

Note: Prepare primers as 50 µM primer stocks. Add an equal amount of each 50 µM primer stock to six different Eppendorf tubes labeled as pool 1,2,3,4,5 and 6. Prepare 10 µM working concentration by diluting each pool 1:5 with Nuclease-Free Water.

8. Mix the following components:

NEBNext Q5 Hot Start HiFi PCR Master Mix	15 µL
Nuclease-Free Water	9.2 µL
Primer pool 1,2,3,4,5 or 6 (10 µM)	1.8 µL
4x SYBR Green	1 µL

9. Add 3 µL of cDNA to each tube/well
10. Perform the following steps in PCR thermocycler with the lid set to 105 °C:

1.	98 °C	30 sec
2.	98 °C	15 sec
3.	65 °C	5 min
GOTO step 2, 40 times		
4.	4 °C	Hold

DNA purification

11. Pool 20 µL from each of the six amplicon pools into a 1.5 ml Eppendorf DNA LoBind tube or 96 well plate
12. Add 1 vol/vol ratio of AMPure XP beads pre-warmed at room temperature
13. Mix thoroughly and incubate for 10 min at room temperature
14. Place the sample on a magnetic stand
15. Incubate for at least 5 min until the liquid appears clear
16. Remove and discard the supernatant
17. Wash the beads twice with 200 µL of freshly prepared 80% ethanol
18. Air-dry the beads at room temperature

Note: do not dry the beads for more than 5–8 min, since this may result in low DNA yield

19. Remove the sample from the magnetic stand
20. Resuspend the beads in 80 µL of Nuclease-Free Water
21. Incubate for 2 min at room temperature
22. Place the sample back on the magnetic stand
23. Incubate for at least 5 min until the liquid appears clear
24. Transfer the supernatant to a new 1.5 µL Eppendorf DNA LoBind tube
25. Check the library concentration using Qubit dsDNA BR kit

Note: Samples can be stored for a long time at –20 °C

Option 2: Multiplex PCR using the ARTIC V3 primer pools

Note: If using IDT ARTIC nCoV-2019 V3 panel pools, prepare 10 µM working concentration by diluting each pool 1:10 with Nuclease-Free Water. The average concentration of each primer in the final reaction is 15 nM.

8. Mix the following components:

NEBNext Q5 Hot Start HiFi PCR Master Mix	12.5 µL
Nuclease-Free Water	2.9 µL
Primer pool 1 or 2 (10 µM)	3.6 µL

9. Add 6 µL of cDNA to each tube/well
10. Perform the following steps in PCR thermocycler with the lid set to 105 °C:

1.	98 °C	30 sec
2.	98 °C	15 sec
3.	63 °C	5 min
GOTO step 2, 35 times		
4.	4 °C	Hold

DNA purification

11. Pool 20 μL from each of the two amplicon pools into a 1.5 ml Eppendorf DNA LoBind tube or 96 well plate
12. Add 0.8 vol/vol ratio of AMPure XP beads pre-warmed at room temperature
13. Mix thoroughly and incubate for 10 min at room temperature
14. Place the sample on a magnetic stand
15. Incubate for at least 5 min until the liquid appears clear
16. Remove and discard the supernatant
17. Wash the beads twice with 200 μL of freshly prepared 80% ethanol
18. Air-dry the beads at room temperature

Note: do not dry the beads for more than 5–8 min, since this may result in low DNA yield

19. Remove the sample from the magnetic stand
20. Resuspend the beads in 40 μL of Nuclease-Free Water
21. Incubate for 2 min at room temperature
22. Place the sample back on the magnetic stand
23. Incubate for at least 5 min until the liquid appears clear
24. Transfer the supernatant to a new 1.5 μL Eppendorf DNA LoBind tube
25. Check the library concentration using Qubit dsDNA BR kit

Note: Samples can be stored for a long time at –20 °C

DAY 2

Note: To process multiple samples in parallel, we performed all reactions until IVT in 384-well plates. We used the I-DOT One nanodispensing device (Dispendix GmBH) to reduce the volumes of each reagent. Other dispensing systems may also be used; however, volumes might have to be adjusted depending on the technical specifications of each instrument.

DNA digestion

26. Dispense manually 5 μL of mineral oil per well in the targeted region of 384-well plates
27. Dispense 10 nL to 50 nL of purified PCR amplicons

Note: The amount of purified PCR amplicons can be increased or reduced depending on the Ct value of the samples

28. Dispense Nuclease-Free Water to a total volume of 350 nL

Note: From now, after dispensing for each step, we shake the plate in a ThermoMixer at 1,000 rpm for 1 min and centrifuge at 3,220 g for 5 min before each incubation

29. Mix the following components:

NlaIII enzyme	50 nL
MseI enzyme	50 nL
10x CutSmart Buffer	50 nL

30. Dispense 150 nL per well
31. Perform the following incubation steps:

1. 37 °C	1 h
2. 65 °C	20 min
3. 4 °C	Hold

Ligation of COVseq adapters

32. Dispense 150 nL of 33 nM COVseq adapter for NlaIII per well
33. Dispense 150 nL of 33 nM COVseq adapter for MseI per well
34. Dispense 700 nL of ligation mix per well (when using standard ligase):

Nuclease-Free Water	250 nL
5x T4 ligase buffer	150 nL
ATP 10 mM	120 nL
BSA 50 mg/ml	30 nL
T4 standard ligase	150 nL

Alternatively, when using rapid ligase:

Nuclease-Free Water	50 nL
5x T4 ligase buffer	300 nL
ATP 10 mM	120 nL
BSA 50 mg/ml	30 nL
T4 rapid ligase	200 nL

35. Perform incubation at 22° C for 1 h followed by inactivation at 70° C for 5 min

Note: When using rapid ligase decrease the incubation time to 30 min.

36. Dispense manually 5 µL of Nuclease-Free Water/33 nM EDTA (for a final concentration of 25 nM) per well
37. Pool the content of multiple wells manually in a 1.5 mL or 5 mL Eppendorf DNA LoBind tube
38. Spin down the tube and carefully remove the upper phase containing mineral oil

Note: The pooling step can be performed by centrifuging the plate upside down at 117 g for 1 min into a collection plate placed at the bottom of the centrifuge.

DNA cleanup

39. Add 1.2 vol/vol ratio of AMPure XP beads pre-warmed at room temperature
40. Mix thoroughly and incubate for 10 min at room temperature
41. Place the sample on a magnetic stand
42. Incubate for at least 5 min until the liquid appears clear
43. Remove and discard the supernatant
44. Wash the beads twice with freshly prepared 80% ethanol (the ethanol volume should be enough to cover the beads)
45. Air-dry the beads at room temperature

Note: do not dry the beads for more than 5–8 min, since this may result in low DNA yield

46. Remove the sample from the magnetic stand
47. Resuspend the beads in 10 µL of Nuclease-Free Water
48. Incubate for 2 min at room temperature
49. Place the sample back on the magnetic stand
50. Incubate for at least 5 min until the liquid appears clear
51. Transfer the supernatant to a new 1.5 µL Eppendorf DNA LoBind tube
52. Check the library concentration using Qubit dsDNA HS kit

Note: Samples can be stored for a long time at -20 °C

In vitro transcription (IVT)

53. Start with 8 µL of supernatant from the previous step
54. Add the following reagents on ice:

rATP+rUTP+rGTP+rCTP*	8 µL
10x T7 polymerase buffer	2 µL
T7 polymerase	1.5 µL
RNaseOUT Recombinant Ribonuclease Inhibitor	0.5 µL

* Prepared from separate rNTP solutions provided with the MEGAscript T7 Transcription Kit

55. Incubate for 14 hours at 37 °C in a PCR thermocycler with the lid set to 70 °C

Note: IVT can also be performed at 37°C for 2 hours to save time.

DAY 3

RNA cleanup

56. Add 1 µL of DNase I (RNase-free) to the IVT product
57. Incubate for 15 min at 37 °C
58. Bring up the volume to 50 µL by adding 29 µL Nuclease-Free Water, then mix with 90 µL (1.8 vol/vol) of RNAClean XP beads pre-warmed at room temperature
59. Mix thoroughly and incubate for 10 min at room temperature
60. Place the sample on a magnetic stand
61. Incubate for at least 5 min until the liquid appears clear
62. Remove and discard the supernatant
63. Wash the beads twice with 200 µL of freshly prepared 70% ethanol
64. Air-dry the beads at room temperature

Note: do not dry the beads for more than 5–8 min, since this may result in low DNA yield

65. Remove the sample from the magnetic stand
66. Resuspend the beads in 9 µL of Nuclease-Free Water
67. Incubate for 2 min at room temperature
68. Place the sample back on the magnetic stand
69. Incubate for at least 5 min until the liquid appears clear
70. Transfer 8.8 µL of supernatant to a new 0.5 µL Eppendorf DNA LoBind tube
71. Check the library concentration with 1 µL using Qubit dsDNA BR kit

RA3 adapter ligation

72. Add 1 µL of 10 µM RA3 adapter to 7.8 µL obtained after RNA cleanup
73. Incubate for 2 min at 70 °C in a PCR thermocycler, then immediately place the sample on ice
74. Add 3.2 µL of the following mix:

RNA ligase buffer	1.2 µL
RNaseOUT Recombinant Ribonuclease Inhibitor	1 µL
T4 RNA ligase truncated	1 µL

75. Incubate for 2 hours at 25 °C in a PCR thermocycler with the lid set to 30 °C

Reverse transcription

76. Add 2 µL of 10 µM RTP primer per sample
77. In a PCR thermocycler, incubate for 2 min at 70 °C
78. Quickly transfer the sample to ice
79. Add 11 µL of the following mix:

5x SSIV buffer	5 μL
25mM dNTPs	1 μL
0.1M DTT	2 μL
RNaseOUT Recombinant Ribonuclease Inhibitor	1 μL
SuperScript IV reverse transcriptase	2 μL

80. Incubate for 20 min at 50°C followed by inactivation for 10 min at 80 °C in a PCR thermocycler with the lid set to 80 °C

Library indexing and amplification

81. Add 16 μL per sample of the desired indexed Illumina primer
82. Add 359 μL of the following mix:

Nuclease-Free Water	143 μL
NEBNext Ultra II PCR Master Mix	200 μL
10 μM RP1 primer	16 μL

83. Divide the final mix over 8 PCR tubes with each containing 50 μL
84. In a PCR thermocycler perform the following cycles:

1.	98 °C	30 sec
2.	98 °C	10 sec
3.	60 °C	30 sec
4.	65 °C	45 sec
GOTO step 2, 10 times		
5	65 °C	5 min
6	4 °C	Hold

Note: 10 PCR cycles are used for an input to the IVT of ≈ 200 ng. Please, adjust PCR cycles accordingly to the input of the in vitro transcription (IVT) step.

Final library cleanup

85. Pool the 8 PCR tubes for each sample and add 0.8 vol/vol ratio of AMPure XP beads pre-warmed at room temperature
86. Mix thoroughly and incubate for 10 min at room temperature
87. Place the sample on a magnetic stand
88. Incubate for at least 5 min until the liquid appears clear
89. Remove and discard the supernatant
90. Wash the beads twice with 1 ml of freshly prepared 80% ethanol
91. Air-dry the beads at room temperature

Note: do not dry the beads for more than 5–8 min, since this may result in low DNA yield

92. Remove the sample from the magnetic stand
93. Resuspend the beads in 50 μL of Nuclease-Free Water

94. Incubate for 2 min at room temperature
95. Place the sample back on the magnetic stand
96. Incubate for at least 5 min until the liquid appears clear
97. Transfer the supernatant to a new 1.5 μL Eppendorf DNA LoBind tube
98. Check the library concentration using Qubit dsDNA HS kit
99. Check the fragment distribution on a Bioanalyzer 2100 using a DNA HS chip

Note: Libraries can be stored for a long time at –20 °C.

2. COVseq workflow with all the steps until IVT performed on I-DOT

As a proof-of-principle, we used SARS-CoV-2 synthetic RNA (Twist Bioscience, cat. no. 102019), which can be handled in a biosafety level 1 (BSL-1) lab, as it was logistically difficult for us to place our I-DOT nanodispenser in a BSL-2 lab. We performed RT, multiplexed PCR, and barcoding by CUTseq sequentially in the same wells of a 384-well plate, without any intermediate purification step until the samples were pooled before IVT. In brief, we dispensed 50 nL of synthetic SARS-CoV-2 RNA containing either 5,000 or 10,000 genome copies into multiple wells of a 384-well plate pre-filled with 5 μL per well of mineral oil. For each sample, we set up six parallel reactions since we used six individual primer pools following the CDC multiplexed PCR protocol (see above). We then dispensed 15 nL per well of a primer-dNTP mix containing 5 nL of 50 μM random hexamers (Thermo Fisher Scientific, cat.no. N80800127), 5 nL of 10 mM dNTPs (Thermo Fisher Scientific, cat.no. R0191) and 5 nL of Nuclease-Free Water (Thermo Fisher Scientific, cat. no. AM9932). We incubated the plate at 65 °C for 5 min and cooled it down immediately on ice. Afterwards, we added 35 nL per well of a first strand synthesis mix containing 20 nL of SuperScript IV buffer (Thermo Fisher Scientific, cat.no. 18090050), 5 nL of 0.1M DTT (Thermo Fisher Scientific, cat.no. 18090050), 5 nL of RNaseOUT (Thermo, 10777-019) and 5 nL of SSIV reverse transcriptase enzyme (Thermo Fisher Scientific, cat.no. 18090050). We incubated the samples at the following temperatures: 23 °C for 10 min, 50 °C for 10 min, 85 °C for 10 min and hold on ice at 4 °C. After RT, we spun down the plate and performed the multiplex PCR by dispensing 250 nL per well of a PCR mix containing 42 nL of Nuclease-Free Water, 12 nL of 4x SYBR Green (Thermo Fisher Scientific, cat.no. S7563), 175 nL of NEBNext Q5 Hot Start HiFi PCR Master Mix (NEB, cat.no. M0543L) and 21 nL of one of the six primer pools (10 μM). We performed PCR, digestion, ligation, and library preparation using the same conditions as described in the step-by-step COVseq protocol above.

3. Supplementary Tables

Supplementary Table 1. Available multiplexed PCR assays for WGS of other viruses in addition to SARS-CoV-2.

Assay	Target	Weblink
CDC Influenza SARS-CoV-2 (Flu SC2) Multiplex Assay	SARS-CoV-2, Influenza A and Influenza B viruses	https://www.fda.gov/media/139743/download
CDC DENV-1-4 rRT-PCR multiplex assay	Dengue virus	https://www.cdc.gov/dengue/healthcare-providers/testing/molecular-tests/realtime.html#anchor_1556657754547

Supplementary Table 2. List of reference sequences used for alignment and phylogenetic analyses.

Genome	Reference
SARS-CoV-2	NC_045512.2
Human	GRCh38
H1N1, Influenza type A	NC_026431.1 - NC_026438.1
Influenza type B	NC_002204.1 - NC_002211.1
Dengue	NC_001474.2
Adapters	Default FastQ-Screen reference
Arabidopsis	Default FastQ-Screen reference
Drosophila	Default FastQ-Screen reference
E Coli.	Default FastQ-Screen reference
Lambda	Default FastQ-Screen reference
Mitochondria	Default FastQ-Screen reference
Mouse	Default FastQ-Screen reference
PhiX	Default FastQ-Screen reference
Rat	Default FastQ-Screen reference
RRNA	Default FastQ-Screen reference
Vectors	Default FastQ-Screen reference
Worm	Default FastQ-Screen reference
Yeast	Default FastQ-Screen reference

4. Supplementary Notes

1. Cumulative cost analysis

To demonstrate the cost effectiveness of COVseq, we compare our method with three commercial kits (CleanPlex, NEBNext and Nextera) for library preparation compatible with SARS-CoV-2 WGS. Specifically, we compare:

- CDC-COVseq: this is the COVseq workflow based on the multiplexed PCR assay developed by the US Centers for Disease Control and Prevention (CDC) depicted in **Fig. 1a** and used throughout this manuscript.
- ARTIC-COVseq: this is the COVseq workflow based on the V3 multiplexed PCR assay developed by the ARTIC network depicted in **Supplementary Fig. 5a**. As discussed below, we are currently using this workflow to sequence dozens of samples per week in the frame of the SARS-CoV-2 genomic surveillance program that we started for the Piemonte Region in Italy (an example of lineages determined using this workflow is provided in **Supplementary Fig. 5b**).
- CleanPlex SARS-CoV-2 FLEX Panel (Paragon Genomics, cat. no. SKU918015): this is a commercial kit featuring a proprietary primer pool for SARS-CoV-2 enrichment by multiplex PCR. We have used this kit to validate some of the samples processed by ARTIC-COVseq (see **Supplementary Fig. 5a**).
- NEBNext Ultra II FS DNA Library Prep Kit (NEB, cat. no. E7805S): this is a widely used kit for preparing DNA NGS libraries, which can be applied to SARS-CoV-2 amplicons generated by one of the multiplexed PCR assays used in COVseq and described above. We have used this kit to technically validate COVseq on the OAS-29 samples, as shown in **Fig. 1e-j**.
- Nextera XT DNA Library Preparation Kit (Illumina, cat. no. FC-131-1024): this is a transposase-based kit for DNA NGS library preparation, which can be applied to SARS-CoV-2 amplicons generated by one of the multiplexed PCR assays used in COVseq.

We perform all cost analyses based on the following assumptions:

- For COVseq: we assume to use a combination of two enzymes (MseI and NlaIII) to digest the pre-amplified SARS-CoV-2 genome and 96 different COVseq adapters (96 for MseI and 96 for NlaIII, see **Supplementary Data 2**) enabling us to pool 96 samples into the same COVseq library. Moreover, we assume to perform all the barcoding reactions in 96-well plates (see **Fig. 1b**) using the I-DOT One contactless nanodispensing device

(Dispendix GmBH), which we previously utilized for high-throughput CUTseq. However, any other contactless device with similar characteristics should be equally effective. Lastly, we assume to perform the ligation step using standard T4 DNA ligase.

- For commercial kits: we assume to use kits containing 96 different library indexes, so that up to 96 libraries (samples) can be sequenced together in the same run (see considerations on sequencing below).

A detailed list of reagents, volumes per reaction, number of reactions and current prices (as of Apr 2021) is available in **Supplementary Data 6**. For simplicity, we omit the cost of plasticware and other consumables (pipette tips, gloves, etc.) from our analysis. To simulate large-scale SARS-CoV-2 sequencing, such as the one that could be performed by a centralized laboratory or a public health agency, we compute how the cumulative reagent cost grows by increasing the number of samples processed up to 100,000. The simulation does not include sequencing costs, which are discussed separately below. The simulation is run through a custom script written in MATLAB, which we make available upon request. The results of the simulation are presented in **Fig. 4a-c** and discussed below.

The cumulative cost curve for preparing libraries is lower using any of the two COVseq workflows compared to the three commercial kits examined, when more than 300 samples are processed. For 1,000 samples, the mean cost per sample is ~$37 using CDC-COVseq and ~$29 using ARTIC-COVseq, since the latter only requires two PCR pools (see **Supplementary Fig. 5a**). When 10,000 samples are processed, the mean cost per sample is reduced to ~$22 using CDC-COVseq and ~$14 using ARTIC-COVseq. For 100,000 samples, the costs get further reduced, reaching ~$20 using CDC-COVseq and ~$13 using ARTIC-COVseq. In comparison, commercial kits have a cumulative cost 3–5 times higher: CleanPlex has the highest cumulative cost (~$77 and ~$73 for 1,000 and 10,000 up to 100,000 samples, respectively), followed by NEBNext (~$67 and ~$69 per sample for 1,000 and 10,000 up to 100,000 samples, respectively) and Nextera (~$65 and ~$62 per sample for 1,000 and 10,000 up to100,000 samples, respectively). The fact that below 300 samples COVseq is slightly more costly is due to the fact that all COVseq reagents must be purchased separately. Therefore, at least initially, the cost is higher because some reagents can only be purchased in quantities larger than the amount actually needed. (NOTE: the oligos required for preparing COVseq adapters account for the largest fraction of the initial investment needed to use COVseq. However, the quantity of adapters that can be prepared in such way is then sufficient to process millions of samples, see **Supplementary Data 6**). However, for a centralized Covid lab or a public health agency lab—which potentially has to process hundreds of samples per week—COVseq clearly

represents the most cost-effective solution currently available. The COVseq cost per sample could be further reduced by performing all reactions until *in vitro* transcription of the pooled samples in small volumes using the I-DOT nanodispensing device (or any other equivalent machine). This is theoretically possible, as we have demonstrated using synthetic SARS-CoV-2 RNA (**Supplementary Fig. 7a, b and Supplementary Methods**). However, to use this workflow routinely, the I-DOT machine should be placed in a BSL-2 environment, which was not possible for us.

2. Considerations on the sequencing platform and number of samples sequenced together

In this work, we have sequenced most of the COVseq libraries on the NextSeq 500 platform in PE150 mode and some of them (OAS-29 samples) on the MiSeq platform in PE300 mode. In general, the number of samples sequenced in the same run depends on two key parameters:

1. The desired breadth and depth of coverage per sample.
2. The number of sample barcodes in COVseq and library indexes in commercial kits.

Based on the experience we have gathered thus far, we recommend aiming at generating 500,000 reads per sample using either PE150 or PE300, which, as we have shown in **Fig. 1j**, allows detecting almost all the variants present in samples with low Ct value ($\leq$ 35). Regarding the second parameter, the number of samples that can be pooled in the same library in COVseq depends on the number of available adapters. In this work, we have used 96 MseI and NlaIII adapters, which allows 96 samples to be pooled together in the same library. However, in **Supplementary Data 2** we also provide the sequences of 384 MseI and NlaIII adapters, which could be useful when processing many samples. For example, assuming to process 100,000 samples in total using 384 COVseq adapters and aiming at generating 0.5 M reads per sample, 100,000 samples split in 261 libraries with 384 samples each could be sequenced on 5 PE150 runs on Illumina's NovaSeq 6000, for a total cost per sample of ~$14 including library preparation and sequencing costs. Comparatively, using one of the three examined commercial kits with 384 indexes to prepare libraries and sequencing on the same platform would be 5 (Nextera) to 7 (NEBNext) times more expensive. Notably, when using commercial kits, since an individual library is prepared from each sample separately, the concentration of each library must be accurately quantified before pooling multiple libraries into the same sequencing run, which further increases the cost and hands-on time per sample. To our knowledge, all of the advantages described make COVseq the most cost-efficient solution for mass-scale genomic surveillance of SARS-CoV-2 currently available.
