# Supplementary material for: COVseq is a cost-effective workflow for mass-scale SARS-CoV-2 genomic surveillance
Source: Nat Commun. 2021 Jun 23;12:3903. doi: 10.1038/s41467-021-24078-9 (PMC8222401; doi:10.1038/s41467-021-24078-9)
Supplement: Supplementary file 9 — Supplementary Data 7 [file 41467_2021_24078_MOESM9_ESM.pdf]

We gratefully acknowledge the following Authors from the Originating laboratories responsible for obtaining the specimens, as well as the Submitting laboratories where the genome data were generated and shared via GISAID, on which this research is based.

All Submitters of data may be contacted directly via www.gisaid.org

Authors are sorted alphabetically.

Accession ID	Originating Laboratory	Submitting Laboratory	Authors
EPI_ISL_1167033, EPI_ISL_1167086	"Dr. Andrija Stampar" Teaching Institute of Public Health, Department of Clinical Microbiology	Istituto di Genomica Applicata	Davide Scaglione; Eleonora Paparelli; Fedrica Cattonaro; Gabriele Magris; Irena Jurman; Jasmina Vranes; Michele Morgante; Slobodanka Radovic; Vera Vendramin
EPI_ISL_763065, EPI_ISL_794737	1-Laboratory of Microbiology, National Reference Lab, Charles Nicolle Hospital; 2-University of Tunis ElManar, Faculty of Medicine of Tunis, LR99ES09, Tunis, Tunisia	1-Clinical and Experimental Pharmacology Lab, LR16SP02, National Center of Pharmacovigilance, University of Tunis El Manar, Tunis, Tunisia. 2-Neurodegenerative diseases and psychiatric troubles, LR18SP03, Razi Hospital, University of Tunis El Manar, Tunis, Tunisia. 3- Ministry of Health, National Observatory of New and Emerging Diseases, 1006, Tunis, Tunisia	Alia Ben Kahla; Alia BenKahla; Asma Ferjani; Awatef El Moussi; Gaies Emna; Guedi Ali Barreh; Habiba Ben Romdhane; Hanen El Jebari; Ilhem Boutiba-Ben Boubaker; Ines Mdini; Jalila Ben Khelil; Maher Kharrat; Mouna Ben Sassi; Mouna Safer; Nissaf Ben Alaya; Riadh Daghfous; Riadh Gouider.; Salma Abid; Sameh Trabelsi; Sana Ferjani; Sarra Chamman; Souissi Amira; Zaineb Hamzaoui
EPI_ISL_1265422	1. Wojewódzka Stacja Sanitarno - Epidemiologiczna w Katowicach (WSSE Katowice); 2. Wojewódzka Stacja Sanitarno - Epidemiologiczna w Kielcach (WSSE Kielce); 3. Wojewódzka Stacja Sanitarno - Epidemiologiczna w Szczecinie (WSSE Szczecin); 4. Wojewódzka Stacja Sanitarno - Epidemiologiczna w Lublinie (WSSE Lublin); 5. Wojewódzka Stacja Sanitarno - Epidemiologiczna w Gorzowie Wielkopolskim (WSSE Gorzów Wielkopolski); 6. Wojewódzka Stacja Sanitarno-Epidemiologiczna w Gdasku (WSSE Gdask); 7. Wojewódzka Stacja Sanitarno-Epidemiologiczna w Poznaniu (WSSE Pozna); 8. Wojewódzka Stacja Sanitarno-Epidemiologiczna w Opolu (WSSE Opole); 9. Wojewódzka Stacja Sanitarno-Epidemiologiczna we Wrocawiu (WSSE Wrocaw); 10. Wojewódzka Stacja Sanitarno-Epidemiologiczna w Krakowie (WSSE Kraków); 11. Wojewódzka Stacja Sanitarno-Epidemiologiczna w Olsztynie (WSSE Olsztyn); 12. Wojewódzka Stacja Sanitarno-Epidemiologiczna w Bydgoszczy (WSSE Bydgoszcz); 13. Wojewódzka Stacja Sanitarno-Epidemiologiczna w Rzeszowie (WSSE Rzeszów); 14. Wojewódzka Stacja Sanitarno-Epidemiologiczna w Biaymstoku (WSSE Biaystok);	1. ViroGenetics - BSL3 Laboratory of Virology, Maopolska Centre of Biotechnology, Jagiellonian University; 2. Human Genome Variation Research Group, Malopolska Centre of Biotechnology, Jagiellonian University;	Branicki, W.; Gromowski, T.; Klajmon, A.; Kowalski, M.; Labaj; Marszalek, K.; Mazur-Panasiuk, N.; P.P.; Pyrc, K.; Szulc, P.
EPI_ISL_876970, EPI_ISL_877034, EPI_ISL_882679, EPI_ISL_882687, EPI_ISL_882742, EPI_ISL_882748, EPI_ISL_918462, EPI_ISL_935135, EPI_ISL_954190, EPI_ISL_954220			
see above	1.AO Universitaria 'S. Giovanni di Dio e Ruggi D'Aragona, Scuola Medica Salernitana' Hospital / 2.UOC di Virologia e Microbiologia, Università della Campania 'L. Vanvitelli' / 3.AO Universitaria 'Federico II' Napoli Hospital / 4.AORN 'San Giuseppe Moscati' Avellino Hospital / 5.AO 'San Pio - presidio G. Rummo' Benevento Hospital / 6.AO 'Sant'Anna e San Sebastiano' Caserta Hospital / 7.PO 'Maria Santissima Addolorata' Eboli Hospital / 8.Biogem Istituto di Ricerche Genetiche	1. Genome Research Center for Health (CRGS) / 2. Laboratory of Molecular Medicine and Genomics(LMMGe) / 3. Center for Research in Pure and Applied Mathematics (CRMPA)	Alessandro Weisz; Alessandro Weisz (Corresponding Author); Alessia Cossu; Andreina Baj; Aniello Gentile; Annamaria Salvati; Antonello Saccomanno; Arnolfo Petruzziello; Assunta Sellitto; Carlo Ferravante; Domenico Memoli; Domenico Palumbo; Edmondo Adorisio; Elena Alexandrova; Emilia Vaccaro; Fausto Sessa.; Francesca Marciano; Francesca Rizzo; Francesca Rizzo (Corresponding Author); Francesco Curcio; Gianluigi Franci; Giorgio Dirani; Giorgio Giurato; Giorgio Giurato (Corresponding Author); Giovanni Nassa; Giovanni Pecoraro; Giuseppe Fenza; Giuseppe Portella; Gregorio Goffredi; Ilaria Terenzi; Jessica Lamberti; Maddalena Schioppa; Maria Grazia Foti; Maria Landi; Marianna Scrima; Mariarosaria Ingino; Massimiliano Galdiero; Maurizio Fumi; Michela Iacobellis; Michele Caraglia; Michele Cennamo; Morena D'Avenia; Oriana Strianese; Pasquale Pagliano; Rita Greco; Roberta Tarallo; Rosanna Piluscio; Silvia Zanoli; Simona Semprini; Sonia Amabile; Stefania Marzinotto; Teresa Rocco; Valeria Mirici Cappa; Vincenzo Rocco; Viola Melone; Vittoria Letizia; Vittorio Sambri; Ylenia D'Agostino
EPI_ISL_1361523, EPI_ISL_1361594	1.AO Universitaria 'S. Giovanni di Dio e Ruggi D'Aragona, Scuola Medica Salernitana' Hospital / 2.UOC di Virologia e Microbiologia, Università della Campania 'L. Vanvitelli' / 3.AO Universitaria 'Federico II' Napoli Hospital / 4.AORN 'San Giuseppe Moscati' Avellino Hospital / 5.AO 'San Pio - presidio G. Rummo' Benevento Hospital / 6.AO 'Sant'Anna e San Sebastiano' Caserta Hospital / 7.PO 'Maria Santissima Addolorata' Eboli Hospital / 8.Biogem Istituto di Ricerche Genetiche / 9. U.O.C. di Genetica Medica e di Laboratorio A.O.R.N., Azienda Ospedaliera di Rilievo Nazionale Antonio Cardarelli, Napoli / 10. Centro di riferimento Oncologico della Basilicata (IRCCS-CROB), Rionero in Vulture (PZ) / 11. Presidio Ospedaliero di Agropoli, Agropoli (SA).	1. Genome Research Center for Health (CRGS) / 2. Laboratory of Molecular Medicine and Genomics(LMMGe) / 3. Center for Research in Pure and Applied Mathematics (CRMPA)	Alessandro Weisz (Corresponding Author); Alessia Cossu; Andreina Baj; Aniello Gentile; Annamaria Salvati; Antonello Saccomanno; Arnolfo Petruzziello; Assunta Sellitto; Carlo Ferravante; Domenico Memoli; Domenico Palumbo; Edmondo Adorisio; Elena Alexandrova; Emilia Vaccaro; Fausto Sessa.; Francesca Marciano; Francesca Rizzo (Corresponding Author); Francesco Curcio; Gianluigi Franci; Giorgio Dirani; Giorgio Giurato (Corresponding Author); Giovanni Nassa; Giovanni Pecoraro; Giuseppe Fenza; Giuseppe Portella; Gregorio Goffredi; Ilaria Terenzi; Jessica Lamberti; Maddalena Schioppa; Maria Grazia Foti; Maria Landi; Marianna Scrima; Mariarosaria Ingino; Massimiliano Galdiero; Maurizio Fumi; Michela Iacobellis; Michele Caraglia; Michele Cennamo; Morena D'Avenia; Oriana Strianese; Pasquale Pagliano; Rita Greco; Roberta Tarallo; Rosanna Piluscio; Silvia Zanoli; Simona Semprini; Sonia Amabile; Stefania Marzinotto; Teresa Rocco; Valeria Mirici Cappa; Vincenzo Rocco; Viola Melone; Vittoria Letizia; Vittorio Sambri; Ylenia D'Agostino
EPI_ISL_831952	ABC	The Public Health Agency of Sweden	Department of Microbiology; The Public Health Agency of Sweden
EPI_ISL_768778	AIID	Irish Coronavirus Sequencing Consortium - National Virus Reference Laboratory	Alejandro Abner Garcia Leon; Gabriel Gonzalez; Michael Carr; Patrick Mallon
EPI_ISL_471546	AMA DR Jose Soares Hungria	Instituto Adolfo Lutz, Interdiciplinary Procedures Center, Strategic Laboratory	Claudia Regina Gonçalves; Claudio Tavares Sacchi; Erica Valessa Ramos Gomes
EPI_ISL_1085036, EPI_ISL_1166226, EPI_ISL_1169161, EPI_ISL_1169478, EPI_ISL_1219055, EPI_ISL_1229558, EPI_ISL_1229566, EPI_ISL_1229632, EPI_ISL_1229811, EPI_ISL_1298637, EPI_ISL_1298733, EPI_ISL_1359213, EPI_ISL_1359327, EPI_ISL_1359382			
see above	ASL Napoli 1 Centro	AMES Centro Polidiagnostico Strumentale S.r.l.	"Giovanni Savarese; Antonella Di Carlo; Antonio Fico"; Eloisa Evangelista; Luigi D'Amore; Luisa Circelli; Maurizio D'Amora; Monica Ianniello; Nadia Petrillo; Raffaella Ruggiero; Roberto Sirica
EPI_ISL_542108, EPI_ISL_542127, EPI_ISL_542141, EPI_ISL_542146, EPI_ISL_542165, EPI_ISL_542224, EPI_ISL_542225, EPI_ISL_542229, EPI_ISL_542233, EPI_ISL_542235, EPI_ISL_542254, EPI_ISL_542256, EPI_ISL_542257, EPI_ISL_542401, EPI_ISL_542420, EPI_ISL_542426			
see above	ASST GOM Niguarda	Dep. Of Oncology and Hemato-Oncology University of Milan	Antonio Piralla; Carlo Federico Perno; Chiara Vismara; Claudia Alteri; Elisa Matarazzo; Fausto Baldanti; Federica Giardina; Federica Novazzi; Luna Colagrossi; Maria Antonello; Massimo Puoti; Monica Tallarita; Oscar Massimiliano Epis; Roberto Fumagalli; Silvia Renica; Stefano Gaiarsa; Valentino Costabile; Valeria Cento
EPI_ISL_694531	AZ SPHL, Arizona Department of Health Services	TGen North	Ashlyn Pfeiffer; Chris French; Darrin Lemmer; Dave Engelthaler; Hayley Yaglom; Jolene Bowers; Megan Folkerts; The Arizona COVID Genomics Union (ACGU)

EPI_ISL_517642, EPI_ISL_517661	Academic Hospital Paramaribo	Erasmus Medical Center	Bas Oude Munnink; Dion Gajadin; Ed Ijzerman; Emmanuelle Munger; Gary Gummels; Ingrid Krishnadath; Lycke Woittiez; Marion Koopmans; Mireille Van de Veer; Princes Wongsowidjojo; Radjesh Ori; Rohma Banwari; Stephen Vreden
EPI_ISL_528934, EPI_ISL_528937, EPI_ISL_528939, EPI_ISL_528947, EPI_ISL_528949	Agenzia di Tutela della Salute di Bergamo	Istituto Zooprofilattico Sperimentale dell'Abruzzo e Molise "G.Caporale"	Ancora M; Cammà C; Curini V; Di Domenico M; Di Pasquale A; Lorusso A; Mangone I; Marcacci M; Puglia I; Rinaldi A; Savini G.
EPI_ISL_420134, EPI_ISL_549091	Akershus University Hospital, Department for Microbiology and Infectious Disease Control	Norwegian Institute of Public Health, Department of Virology	Hilde Elshaug; Hilde Synnøve Vollan; Kamilla Heddeland Instefjord; Karoline Bragstad; Kathrine Stene-Johansen; Olav Hungnes; Rasmus Riis Kopperud
EPI_ISL_854592	Alberta Precision Labs (APL)	Alberta Precision Labs (APL)	Berenger B; Bernier F; Chui L; Croxen M; Gordon P; Kellner J; Lam LG; Li V; Ma R; Melin A; Pabbaraju K; Tipples G; Wong A; Zelyas N
EPI_ISL_1385811	Alfa Diagnostica LLC	ONCOGENE LLC	ONCOGENE LLC
EPI_ISL_583884, EPI_ISL_853997	Austrian Agency for Health and Food Safety (AGES)	Bergthaler laboratory, CeMM Research Center for Molecular Medicine of the Austrian Academy of Sciences	Adi Steinrigl; Alexander Lercher; Alexandra Popa; Andreas Bergthaler; Anna Schedl; Benedikt Agerer; Christian Paar; Christoph Bock; Christoph Bock; Daniela Schmid; Dorothee von Laer; Elisabeth Puchhammer-Stoeckl; Franz Allerberger; Gernot Walder; Gregor Hörmann; Guenter Weiss; Gunther Vogl; Henrique Colaco; Jakob-Wendelin Genger; Jan Laine; Judith Aberle; Kinga Rigler-Hohenwarter; Lukas Endler; Manfred Nairz; Mark Smyth; Martin Senekowitsch; Martin Senekowitsch; Michael Schuster; Michael Schuster; Peter Hufnagl; Peter Obrist; Rainer Gattringer; Sabine Sussitz-Rack; Stephan Aberle; Thomas Penz; Wegene Borena
EPI_ISL_965131	Azienda Ospedaliera San Giovanni Addolorata	INMI Lazzaro Spallanzani IRCCS	A Di Caro; B Bartolini; CEM Gruber; E Giombini; F Messina; M Gaudio; M Rueca; MR Capobianchi; O Butera; PM Placanica
EPI_ISL_1386115	Azienda Sanitaria Locale di Piacenza - Presidio Ospedaliero - Laboratorio di Microbiologia	U.O. Microbiologia, Laboratorio Unico Centro Servizi - AUSL della Romagna	Giorgio Dirani; Lo Cascio Giuliana; Schiavo Roberta; Silvia Zannoli; Vittorio Sambri
EPI_ISL_1063455, EPI_ISL_1222805, EPI_ISL_1222815	Azienda Sanitaria dell'Alto Adige Laboratorio Aziendale di Microbiologia e Virologia	Istituto di Genomica Applicata	Davide Scaglione; Eleonora Paparelli; Elisa Masi; Elisabetta Giacobazzi; Elisabetta Pagani; Gabriele Magris; Irena Jurman; Irene Bianconi; Michele Morgante; Stefanie Wieser; Vera Vendramin
EPI_ISL_995741	BBMP Urban PHC	Department of Neurovirology, National Institute of Mental Health and Neurosciences (NIMHANS)	Anita S Desai; Anson Kunjumon George; Chitra Pattabiraman; Darshan Sreenivas; Harsha.P.K; Nakka Vijay Kiran Reddy; Pramada Prasad; Risha Rasheed; V Ravi
EPI_ISL_969294	BCCDC Public Health Laboratory	BCCDC Public Health Laboratory	Ana Pacagnella; Corrinne Ng; Dan Fornika; John Tyson; Kim Macdonald; Kimia Kamelian; Linda Hoang; Loretta Janz; Mel Krajden; Prystajecky Natalie; Robert Azana Terry Snutch; Shannon Russell
EPI_ISL_1191697, EPI_ISL_1200690, EPI_ISL_1297362	BIOMNIS LYON	CNR Virus des Infections Respiratoires - France SUD	Antonin Bal; Bruno Lina; Gregory Destras; Gwendolyne Burfin; Hadrien Regue; Laurence Josset; Martine Valette; Quentin Semanas
EPI_ISL_1219499	BIOR	Latvian Biomedical Research and Study Centre	Daina Pule; Davids Fridmanis; Guntars Zarins; Irena Meistere; Ivars Silamikelis; Janis Klovins; Janis Pjalkovskis; Jurijs Perevoscikovs; Kaspars Megnis; Laila Silamikele; Lauma Freimane; Laura Ansone; Liga Birzniece; Monta Ustinova; Nikita Zrelovs; Uga Dumpis; Una Krumina; Vita Rovite
EPI_ISL_1265443	BTKLPP Kelas I Makassar	Eijkman Institute for Molecular Biology, Ministry of Research and Technology/National Agency for Research and Innovation; National Institute of Health Research and Development	Amin Soebandrio; Edison Johar; Frilasita A Yudhaputri; Hana Apsari Pawestri; Hidayat Trimarsanto; Iskandar Adnan; Khin Saw Myint; Lydia V. Panggalo; Safarina G Malik; Slamet; Sukma Oktavianthi; Vivi Setiawaty; Willy Agustine
EPI_ISL_1159375	Bahman Hospital	Laboratory of Molecular Biology and Cancer Immunology,Lebanese University Public Health England	Fadi Abdel Sater; Steven Pullan
EPI_ISL_833333	Batangas City Health Office	Research Institute for Tropical Medicine	Catalino Demetria; Daria Manalo; Edelwisa Mercado; Francisco Gerardo Polotan; Hannah Leah Morito; Inez Andrea Medado; John Leonard Chan; Kirstyn Brunker; Ma Angelica Tujan; Othoniel Jan Onza
EPI_ISL_1117376	Beijing Center for Disease Prevention and Control	Beijing Center for Disease Prevention and Control	Bing Lyu; Daitao Zhang; Fu Li; Lijuan Chen; Quanyi Wang; Shujuan Cui; Yang Pan; Zhaomin Feng; Zhichao Liang
EPI_ISL_509713	Belize Ministry of Health	Pathogen Discovery, Respiratory Viruses Branch, Division of Viral Diseases, Centers for Disease Control and Prevention	Anna Uehara; Clinton Paden; Haibin Wang; Jing Zhang; Krista Queen; Suxiang Tong; Yan Li; Ying Tao
EPI_ISL_780386, EPI_ISL_780410	Bermuda Government Molecular Diagnostics Laboratory (MDL)	Respiratory Virus Unit, National Infection Service, Public Health England	Dr Ayoola Oyinloye (Bermuda); Dr Carika Weldon (Bermuda); PHE Covid Sequencing Team
EPI_ISL_429995, EPI_ISL_730511, EPI_ISL_755261	Biolab Diagnostic Laboratories	Andersen lab at Scripps Research	Ahmad Tibi; Amid Abdelnour with SEARCH Alliance San Diego; Issa Abu-Dayyeh; Lama Hussein; Lina Mohammad; Zein Naber
EPI_ISL_516083	Biomedical Sciences and Public Health, Polytechnic University of Marche	Biomedical Sciences and Public Health, Polytechnic University of Marche	Alessandrini, F.; Bagnarelli, P.; Caucci, S.; Di Sante, L.; Melchionda, F.; Menzo, S.; Onofri, V.; Tagliabracci, A.; Turchi, C.
EPI_ISL_1213509	Bohol Containerized PCR Laboratory	Philippine Genome Center	Alethea R. de Guzman; Anna Ong-Lim; Arianne A. Zamora; Asia Louisa U. Chong; Benedict A. Maralit; Candice Francheska B. Tambaoan; Carlo M. Lapid; Celia Carlos; Devon Ray Pacial; Edsel Maurice Salvaña; El King D. Morado; Eva Maria Cutiongco-de la Paz; Francis A. Tablizo; Irish Coleen A. Asin; Jaime C. Montoya; Jan Michael C. Yap; Jo-Hannah S. Llames; John Q. Wong; Joshua Gregor A. Dizon; Juan Antonio R. Magalang; Karol Sophia Agape R. Padilla; Kenneth M. Kim; Kris P. Punayan; Marc Edsel C. Ayes; Marc Jerrone R. Castro; Maria Rosario Singh-Vergeire and Cynthia P. Saloma; Maria Sofia L. Yangzon; Marissa Alejandria; Razel Nikka M. Hao; Rianna Patricia S. Cruz; Sheila Mae M. Araiza
EPI_ISL_596453	Booali laboratory, Qom, Iran. Department of Virology, School of Public Health, Tehran University of Medical Sciences, Tehran, Iran.	Genetics Research Center, University of Social Welfare and Rehabilitation Sciences	Ali Jafarpour; Azam Ghaziasadi; Hossein Najmbadi; Khadijeh Jalalvand; Kimia Kahrizi; Marzieh Mohseni; Mohammad Khazeni; Seyed Amir Momeni; Seyed Mohammad Jazayeri; Seyedeh elham Mortazavi; Zohreh Fattahi
EPI_ISL_1363121	Borneo Medical Centre	Institute of Health and Community Medicine	Chan Chia Jui; Chua Hock Hin; David Perera; Ooi Mong How; Tonnii Sia Loong Loong; Wong Jyn Shan; Wong Kiing Aik
EPI_ISL_1307719	Botswana Harvard HIV Reference Laboratory	Botswana Harvard HIV Reference Laboratory	Boitumelo Zuze; Botshelo Radibe; David Lawrence; Dorcas Maruapula; Joseph Makhema; Legodile Kooepile; Mosepele Mosepele; Roger Shapiro; Shahin Lockman; Sikhulile Moyo; Simani Gaseitsiwe; Thongbotho Mphoyakgosi; Wonderful T. Choga
EPI_ISL_1001001, EPI_ISL_1034760, EPI_ISL_1046792	Bundeswehr Institute of Microbiology	Bundeswehr Institute of Microbiology	Alexandra Rehn; Enrico Georgi; Malena Bestehorn-Willmann; Markus Antwerpen; Mathias Walter; Mike Pillukat; Roman Wölfel; Sabine Zange
EPI_ISL_833336	Bureau of Quarantine	Research Institute for Tropical Medicine	Catalino Demetria; Daria Manalo; Edelwisa Mercado; Francisco Gerardo Polotan; Hannah Leah Morito; Inez Andrea Medado; John Leonard Chan; Kirstyn Brunker; Ma Angelica Tujan; Othoniel Jan Onza
EPI_ISL_1371897	C H DE LA POLYNESIE FRANCAISE	CNR Virus des Infections Respiratoires - France SUD	Antonin Bal; Bruno Lina; Gregory Destras; Gwendolyne Burfin; Hadrien Regue; Laurence Josset; Martine Valette; Quentin Semanas
EPI_ISL_1313574	CENTRE HOSPITALIER DU HAUT BUGEY	CNR Virus des Infections Respiratoires - France SUD	Antonin Bal; Bruno Lina; Gregory Destras; Gwendolyne Burfin; Hadrien Regue; Laurence Josset; Martine Valette; Quentin Semanas
EPI_ISL_644252	CEPHR / Mater Hospital	Irish Coronavirus Sequencing Consortium - National Virus Reference Laboratory	Alejandro Abner Garcia Leon; Gabriel Gonzalez; Michael Carr; Patrick Mallon
EPI_ISL_683835	CICM	Malaria Research and Training Center (MRTC-Parasito)	Abdoulaye Djimde; Antoine Dara
EPI_ISL_910236, EPI_ISL_910255, EPI_ISL_1380434	CSIR-Centre for Cellular and Molecular Biology	CSIR-Centre for Cellular and Molecular Biology	Archana Bharadwaj Siva; B Himasri; Blessy B John; Divya Tej Sowpati; Karthik Bharadwaj Tallapaka; Lamuk Zaveri; Namami Gaur; Payel Mukherjee; Pratheusa Maccha; Priya Singh; Purushotham Vodnala; Rakesh K Mishra; Sofia Banu; Tulasi Nagabandi; Viswagithe S L
EPI_ISL_935804	Cadham Provincial laboratory	National Microbiology Laboratory (NML)	Anna Majer; Anneliese Landgraff; CanCOGeN's metadata curation team; Darian Hole; David Alexander; Elsie Grudeski; Gary Van Domselaar; Grace Seo; Jared Bullard; Jennifer Tanner; Kerry Dust; Kirsten Biggar; Madison Chapel; Morag Graham; Natalie Knox; Nathalie Bastien; Paul Van Caeseele; Philip Mabon; Public Health Agency of Canada CanCOGeN team; Rhiannon Huzarewich; Russell Mandes; Shari Tyson; Timothy Booth; Yan Li
EPI_ISL_406036, EPI_ISL_410044	California Department of Public Health	Pathogen Discovery, Respiratory Viruses Branch, Division of Viral Diseases, Centers for Dieases Control and Prevention	Anna Uehara; Brett L. Whitaker; Brian Lynch; Clinton R. Paden; Janna' R. Murray; Jing Zhang; Krista Queen; Lijuan Wang; Senthil Kumar K. Sakthivel; Shifaq Kamili; Stephen Lindstrom; Susan I. Gerber; Suxiang Tong; Xiaoyan Lu; Yan Li; Ying Tao
EPI_ISL_1096140	Cambodian National Public Health Laboratory, National	Virology Unit, Institut Pasteur du Cambodge	Chau Darapheak; Chin Savuth; Erik A Karlsson; Kraing Sidonn; Ly Sovann; Sokhoun Yann; Veasna Duong; Yi Sengdoeurn

	Institute of Public Health		
EPI_ISL_534231	Capio S:t Gorans sjukhus	The Public Health Agency of Sweden	Anna Risberg; Anna-Malin Linde; Karin Tegmark-Wisell; Maria Lind Karlberg; Mattias Haukland; Mia Brytting; Olov Svartstrom; Oskar Karlsson Lindsjo; Petra Edquist; Reza Advani; Sandra Broddesson
EPI_ISL_806783	Casa di cura Di Lorenzo- Avezzano	Istituto Zooprofilattico Sperimentale dell'Abruzzo e Molise "G. Caporale"	Ancora M; Calistri P; Cammà C; Curini V; Di Domenico M; Di Pasquale A; Lorusso A; Mangone I; Marcacci M; Puglia I; Rinaldi A; Savini G
EPI_ISL_913086, EPI_ISL_913089	Center for Virology	Center for Virology	Irene Goerzer; Jeremy V. Camp; Monika Redlberger-Fritz; Stephan W. Aberle
EPI_ISL_438045, EPI_ISL_583707, EPI_ISL_853836, EPI_ISL_853888, EPI_ISL_853894	Center for Virology, Medical University of Vienna	Bergthaler laboratory, CeMM Research Center for Molecular Medicine of the Austrian Academy of Sciences	Adi Steinrigl; Alexander Lercher; Alexandra Popa; Andreas Bergthaler; Anna Schedl; Benedikt Agerer; Christian Paar; Christoph Bock; Christoph Bock; Daniela Schmid; Dorothee von Laer; Elisabeth Puchhammer-Stoeckl; Franz Allerberger; Gernot Walder; Gregor Hörmann; Guenter Weiss; Gunther Vogl; Henrique Colaco; Jakob-Wendelin Genger; Jan Laine; Judith Aberle; Kinga Rigler-Hohenwarter; Lukas Endler; Manfred Nairz; Mark Smyth; Martin Senekowitsch; Martin Senekowitsch; Michael Schuster; Michael Schuster; Peter Hufnagl; Peter Obrist; Rainer Gattringer; Sabine Sussitz-Rack; Stephan Aberle; Thomas Penz; Wegene Borena
EPI_ISL_1093481	Center of Advanced Studies and Technology, Molecular Genetics Laboratory	Center of Advanced Studies and Technology, Molecular Genetics Laboratory	Anaclerio Federico; Damiani Verena; De Fabritiis Simone; Ferrante Rossella; Mandatori Domitilla
EPI_ISL_420082	Centers for Disease Control, R.O.C. (Taiwan)	Centers for Disease Control, R.O.C. (Taiwan)	Ji-Rong Yang; Jung-Jung Mu; Ming-Tsan Liu; Yu-Chi-Lin
EPI_ISL_815257	Centogene	Centogene	Krishna Kumar Kandaswamy; Peter Bauer; Vivi Hue-Trang Lieu
EPI_ISL_794593	Central Laboratories, Egyptian Ministry of Health and Population	Central Laboratories, Egyptian Ministry of Health and Population	A.E.; Ali; El Guindy; El Sayes; El Taweel, A.; El-Shesheny, R.; Gomaa, M.; Kamel; Kandeil, A.; Kayali, G.; Kayed; Khalifa; Kutkat, O.; M.A.; M.K.; M.N.; Mahmoud; Mahrous, N.; Moatasim, Y.; Mostafa, A.; N.M.; Naguib, A.; Roshdy; S.H.; Saleh, M.; Shawky, S.; Shehata, M.; W.H.
EPI_ISL_1222766	Central Scientific Research Department, Gomel State Medical University,	Laboratory of Genomics and Bioinformatics of the Forest Research Institute of the NAS of Belarus	A.A.; A.S.; Baranov; E.V.; I.O.; Kiryanov; L.V.; O.V.; O.Y.; Osipkina; P.S. and Mozharovskaya; Padutov; Panteleev; S.V.; Shaforost; Stoma; V.E.; Voropaev; Zyatskov
EPI_ISL_1312563	Centrala laboratorija	Latvian Biomedical Research and Study Centre	Davids Fridmanis; Guntars Zarins; Ivars Silamikelis; Jana Osite; Janis Klovins; Janis Pjalkovskis; Jurijs Perevoscikovs; Kaspars Megnis; Laila Silamikele; Lauma Freimane; Laura Ansone; Liga Birzniece; Marta Priedite; Monta Ustinova; Nikita Zrelovs; Uga Dumpis; Una Krumina; Vita Rovite
EPI_ISL_1357601	Centre For Biotechnology Research And Development	THE AFRICA GENOMICS CENTRE AND CONSULTANCY	Adede Hawi; Cecilia Waruhiu; Damaris Matoke-Muhia; George Michuki; John Njuguna; Lilian Kanjau; Ravena Mubichi
EPI_ISL_539574, EPI_ISL_539576	Centre de Recherches Medicales de Lambarene (CERMEL)	Department of Emerging Infectious Diseases, Institute of Tropical Medicine, Nagasaki University	Akim A. Adegnika; Bertrand Lell; Haruka Abe; Jiro Yasuda; Rodrigue Bikangui; Yuri Ushijima
EPI_ISL_837556, EPI_ISL_837560, EPI_ISL_837571	Centro Nacional de Enfermedades Tropicales (CENETROP)	Laboratory of Respiratory Viruses and Measles, Oswaldo Cruz Institute, FIOCRUZ	Ana Carolina Mendonca; Anna Carolina Paixao; Cinthia Avila; Fernando Motta; Luciana Appolinario; Marilda Siqueira on behalf of the Fiocruz COVID-19 Genomic Surveillance Network; Paola Resende; Roxana Loayza
EPI_ISL_629019	Centro de Biotecnología Vegetal, Universidad Andrés Bello, Center for Genome Regulation	Center for Mathematical Modeling and Center for Genome Regulation. Santiago, Chile	Allende ML; Arriagada G; Bastias M; Bustos F; Castro E; González M; M; Maass A; Meneses C.; Montecino; Orellana A; Sanhueza D; Travisany D
EPI_ISL_1396484	Centro de Tecnología en Salud Pública de la Universidad Nacional de Rosario	Laboratorio Mixto de Biotecnología Acuática (LMBA) on behalf of 'Proyecto Argentino Interinstitucional de genomica de SARS-CoV-2' (PAIS Consortium)	Adriana Giri; Agustina Cerri; Ana Cavatorta; Ana Paletta; Diego Chouhy; Elisa Bolatti; Elizabeth Tapia (argenTAG); Federico Remes Lenicov; Flavio Spetale; Gastón Viarengo; Ignacio García Labari; Javier Murillo; Joaquín Ezpeleta; Julian Acosta; Laura Angelone; Leandro Ciappina; María Re; Pablo Casal; Pilar Bulacio; Silvana Spinelli; Silvia Arranz; Sofía Lavista Llanos; Vanina Villanova; Victoria Posner
EPI_ISL_450524	Centrl Laboratorija	Latvian Biomedical Research and Study Centre	Ivars Silamielis; Jana Oste; Jnis Kloviš; Kaspars Megnis; Marta Priedte; Monta Ustinova; Stella Lapia; Uga Dumpis; Vita Rovte; ikita Zrelovs
EPI_ISL_754004	Charité Universitätsmedizin Berlin, Institut für Virologie/Labor Berlin	Charité Universitätsmedizin Berlin, Institut für Virologie	Barbara Mühlemann; Christian Drosten; Julia Schneider; Jörn Beheim-Schwarzbach; Talitha Veith; Terry Jones; Victor M Corman
EPI_ISL_477133, EPI_ISL_906089, EPI_ISL_1360450	Child Health Research Foundation	Child Health Research Foundation	Afroza Akter Tanni; Arif Mohammad Tanmoy; CHRF Bangladesh Genomics Team; Maksuda Islam; Md Hafizur Rahman; Md Saiful Islam Sajib; Roly Malaker; Samir K Saha; Senjuti Saha; Sharmistha Goswami; Syed Muktadir Al Sium
EPI_ISL_447423	Clinical Microbiology Laboratory, Sheba Medical Center	Stern Lab	Stern Lab
EPI_ISL_1097498	Colorado Department of Public Health and Environment	Colorado Department of Puplic Health and Environment	Diana Ir; Emily A. Travanty; Laura Bankers; Molly C. Hetherington-Rauth; Sarah Elizabeth Totten; Shannon Ely; Shannon R. Matzinger
EPI_ISL_632276, EPI_ISL_632284, EPI_ISL_632285, EPI_ISL_681301	Communicable Disease Laboratory, Public Health Directorate	Communicable Disease Laboratory, Public Health Directorate	AlAbbas, Z.; AlHujairi, Z.; AlTaif, Z.; AlWasti, H.; Altaif, Z.; Alwasti, H.
EPI_ISL_1091201, EPI_ISL_1091236	Croatian Institute of Public Health	Croatian Institute of Public Health	Irena Tabain; Ivana Ferenak
EPI_ISL_1055818	DIP. PREV. AVEZZANO SERVIZIO DI IGIENE EPIDEMIOLOGIAE SANITA' PUBBLICA	Istituto Zooprofilattico Sperimentale dell'Abruzzo e Molise "G. Caporale"	Ancora M; Calistri P; Cammà C; Curini V; Delli Compagni E; Di Domenico M; Di Pasquale A; Lorusso A; Mangone I; Marcacci M; Puglia I; Rinaldi A; Savini G; Scialabba S
EPI_ISL_812735	DOHMH Morrisania	New York City Public Health Laboratory	Jade Wang; et al.
EPI_ISL_1340756, EPI_ISL_1340764	Departamento de Virologia, Laboratorio Central de Salud Pública, Avenida Venezuela y Teniente Escurra, Asunción, Paraguay	Laboratory of Respiratory Viruses and Measles, Oswaldo Cruz Institute, FIOCRUZ	Alice Sampaio Rocha; Ana Carolina Mendonca; Anna Carolina Paixao; Cynthia Vazquez; Fernando Motta; Luciana Appolinario; Marilda Siqueira on behalf of the Fiocruz COVID-19 Genomic Surveillance Network; Paola Resende; Renata Serrano Lopes
EPI_ISL_516924, EPI_ISL_516928	Department for Molecular Diagnostics, Centre for Medical Microbiology, Institute of Public Health of Montenegro	Charite Universitatsmedizin Berlin, Institut fur Virologie	Barbara Muehlemann; Christian Drosten; Julia Schneider; Jörn Beheim-Schwarzbach; Marija Govedarica and Danijela Vujoševi; Talitha Veith; Terry Jones; Victor M Corman
EPI_ISL_1013611	Department for Molecular Diagnostics, Centre for Medical Microbiology, Institute of Public Health, Montenegro	Charité Universitätsmedizin Berlin, Institut für Virologie	Barbara Mühlemann; Christian Drosten; Danijela Vujoševi; Julia Schneider; Julia Tesch; Jörn Beheim-Schwarzbach; Marija Govedarica; Talitha Veith; Terry Jones; Tobias Bleicker; Victor M Corman
EPI_ISL_447055, EPI_ISL_754180	Department for Virology, Molecular Biology and Genome Research, R. G. Lugar Center for Public Health Research, National Center for Disease Control and Public Health (NCDC) of Georgia.	Department for Virology, Molecular Biology and Genome Research, R. G. Lugar Center for Public Health Research, National Center for Disease Control and Public Health (NCDC) of Georgia.	Adam Kotorashvili; Amiran Gamkrelidze.; Ana Papkiauri; Ann Machablishvili; Anna Kasradze; Davit Tsaguria; Ekaterine Khmaladze; Ekaterine Zangaladze; Ekaterine Zhghenti; Giorgi Tomashvili; Gvantsa Brachveli; Gvantsa Chanturia; Irma Burjanadze; Ketevan Sidamonidze; Khatuna Zakhashvili; Lela Sabadze; Lela Urushadze; Magda Dgebuadze; Maia Alkhazashvili; Mari Gavashelidze; Mariam Zakalashvili; Marine Murtskhvaladze; Meri Pantsulaia; Nato Kotaria; Nino Berishvili; Paata Imnadze; Roena Sukhiashvili; Salome Javashvili; Tamar Jashiashvili; Tata Imnadze; Tea Tevdoradze
EPI_ISL_515112	Department of Biochemistry, Cell and Molecular Biology	WACCBIP, University of Ghana	A.K.; Adu, B.; Amenga-Etego; Ampofo, W.; Amuzu; Anang; Arjarquah, A.; Asante, I.; Awandare; Bediako, Y.; Boatemaa, L.; Bonney, E.; Bonney, K.; C.M.; D.S.; Eshun, M.; G.A.; G.B.; J.K.; J.M.; Kotey, E.; Kumordjie, S.; Kyei; L.N.; Magnussen, V.; Morang'a; Mutungi; Ngoi; Quashie, P.; Tei-Maya, F.
EPI_ISL_884855, EPI_ISL_944715	Department of Biochemistry, Cell and Molecular Biology, West African Centre for Cell Biology of Infectious Pathogens (WACCBIP), University of Ghana	Department of Biochemistry, Cell and Molecular Biology, West African Centre for Cell Biology of Infectious Pathogens (WACCBIP), University of Ghana	A.-K.; A.B.; Abass; Adusei-Poku, M.; Akoriyea; Amenga-Etego; Amoako, E.; Ampofo; Amuzu; Asante, I.; Awandare; Bediako, Y.; Boakye; Bonney; Bonney, E.; C.M.; D.S.; Diallo; E.B.; G.A.; J.H.; J.K.; J.M.; Kibinge, N.; Kumi-Ansah, F.; L.N.; Magnussen, V.; Mohammed, A.; Morang'a; N.T.; Ndam; Ngoi; O.D.; Odoom; Odoom, T.; Ofori-Boadu, L.; Quansah; Quashie, P.; S.K.; Saiid, S.; Tapela, K.; Tei-Maya, F.; W.K.
EPI_ISL_907075, EPI_ISL_956332	Department of Biology, University of Basrah	Department of Biology, University of Basrah	A.I.; Abu-Ali; H.F. and Al-Badran; H.M. and Al-Badran; I.F.
EPI_ISL_424629, EPI_ISL_455958, EPI_ISL_498147, EPI_ISL_833188, EPI_ISL_930618	Department of Clinical Microbiology	GIGA Medical Genomics	Axelle Chaslain; Bouchra Boujemla; Cécile Meex; Céline Fombellida-Lopez; Keith Durkin; Maria Artesi; Marie-Pierre Hayette; Pierrette Melin; Raphaël Boreux; Sébastien Bontems; Vincent Bours; Vincent Bours.
EPI_ISL_412974	Department of Infectious Diseases, Istituto Superiore di Sanità, Rome, Italy	Virology Laboratory, Scientific Department, Army Medical Center	Andrea Ciammaruconi; Antonella Fortunato; Antonella Marchi; Concetta Fabiani; Eleonora Benedetti; Filippo Molinari; Florigio Lista; Giancarlo Petralito; Giovanni Faggioni; Paola Stefanelli; Riccardo De Santis; Silvia Fillo; Stefano Fiore
EPI_ISL_1200636	Department of Infectious Diseases, Istituto Superiore di Sanità, Rome, Italy; AOR San Carlo, Potenza, Italy	Department of Infectious Diseases, Istituto Superiore di Sanità	Alessandra Lo Presti; Angela Di Martino; Angela Menchise; Anna Curci; Antonio Picerno; Lopizzo Teresa; Manuela Marra; Marco Crescenzi; Maria Carollo; Paola Stefanelli; Stefano Fiore
EPI_ISL_892732	Department of Infectious Diseases, Kobe Institute of Health	Pathogen Genomics Center, National Institute of Infectious Diseases	Kentaro Itokawa; Makoto Kuroda; Masanori Hashino; Rina Tanaka; Tsuyoshi Sekizuka

EPI_ISL_422413, EPI_ISL_1020316	Department of Laboratory Medicine, National Taiwan University Hospital	Microbial Genomics Core Lab, National Taiwan University Centers of Genomic and Precision Medicine	Chiao-Ling Li; Pei-Jer Chen; Shan-Chwen Chang; Shiou-Hwei Yeh; Sui-Yuan Chang; Ya-Yun Lai; You-Yu Lin
EPI_ISL_977590	Department of Medical Microbiology, Hospital Pengajar Universiti Putra Malaysia	Malaysia Genome Institute	Avisha Richards; Azrin Ahmad; Enizza Kasim; Hui-Yee Chee; Irni Suhayu Sapian; Mohd Faizal Abu Bakar; Mohd Noor Mat Isa; Muhammad MI; Narcisse Joseph; Nor Azfa Johari; Nor Zahrin Hasran; Nurhezreen Md Iqbal; Shamsidar Sopie; Siti Noraini Othman; Syafinaz Amin-Nordin; Yusuf Muhammad Noor
EPI_ISL_501182	Department of Medical Microbiology, University Malaya Medical Centre	Department of Medical Microbiology, Faculty of Medicine, University of Malaya	I-Ching SAM; Jennifer Chong; University Malaya Medical Centre COVID Team; Yoke Fun CHAN; Yoong Min CHONG
EPI_ISL_512844	Department of Medical Research	DMR_Myanmar	Aung Kyaw Kyaw; Aung Zaw Latt; Hlaing Myat Thu; Hnin Ohnmar Soe; Htin Lin; Kay Thi Aye; Lai Lai San; Myat Htut Nyunt; Nan Aye Thida Oo; Ni Ni Zaw; Phyu Win Ei; Su Mon Win; Theingi Win Myat; Wah Wah Aung; Yi Yi Kyaw; Zaw Than Htun
EPI_ISL_1117891	Department of Microbiology, University Innsbruck	Bergthaler laboratory, CeMM Research Center for Molecular Medicine of the Austrian Academy of Sciences	Andreas Bergthaler; Anna Schedl; Bekir Erguner; Benedikt Agerer; Christoph Bock; Jan Laine; Lukas Endler; Maelle Le Moing; Martin Senekowitsch; Michael Schuster; Thomas Penz
EPI_ISL_463747, EPI_ISL_1164660, EPI_ISL_1164697, EPI_ISL_1164747	Department of Molecular Virology, Cyprus Institute of Neurology and Genetics	Department of Molecular Virology, Cyprus Institute of Neurology and Genetics	Anastasis Oulas; Andreas Hadjisavvas; Christina Christodoulou; Christina Tryfonos; Dana Koptides; Denise Alexandrou; George Krashias; George Spyrou; Jan Richter; Maria Loizidou; Mihalis Panayiotidis; Olga Kalakouta; Pavlos Fanis; Stavros Bashiardes
EPI_ISL_1379435	Department of Molecular and Translational Medicine, Section of Microbiology, University of Brescia, ASST Spedali Civili, Brescia	Department of Molecular and Translational Medicine, Section of Microbiology, University of Brescia, ASST Spedali Civili, Brescia	Alberto Zani; Arnaldo Caruso; Erika Scaltriti; Francesca Caccuri; Serena Messali; Simona Fiorentini
EPI_ISL_596455	Department of Pathology, School of Medicine, Imam Khomeini Hospital, Tehran University of Medical Sciences	Genetics Research Center, University of Social Welfare and Rehabilitation Sciences	Ali Jafarpour; Alireza Abdollahi; Azam Ghaziasad; Azam Ghaziasadi; Azar Hadadi; Hossein Najmabadi; Khadijeh Jalalvand; Kimia Kahrizi; Marzieh Mohseni; Reza Najafipour; Saber Soltani; Seyed Mohammad Jazayeri; Seyedeh elham Mortazavi; Zohreh Fattahi
EPI_ISL_1289872	Department of Public Health Microbiology Ljubljana, National Laboratory for Health, Environment and Food	Department of Public Health Microbiology Ljubljana, National Laboratory for Health, Environment and Food	José Gonçalves; Katarina Prosenc; Martin Bosilj; Metka Paragi; Tom Koritnik
EPI_ISL_582509	Department of Respiratory and other Viral Infections of L.V.Gromashevsky Institute of Epidemiology & Infectious Diseases NAMS of Ukraine	Department of Respiratory and other Viral Infections of L.V.Gromashevsky Institute of Epidemiology & Infectious Diseases NAMS of Ukraine, JSC "Farmak"	Alla Mironenko; Andriy Goy; Ihor Kravchuk; Larysa Radchenko; Ludmyla Bolotova; Nataliia Teteriuk
EPI_ISL_1122014	Department of Respiratory and other Viral Infections of L.V.Gromashevsky Institute of Epidemiology & Infectious Diseases NAMS of Ukrain	Department of Respiratory and other Viral Infections of L.V.Gromashevsky Institute of Epidemiology & Infectious Diseases NAMS of Ukrain, JSC "Farmak"	Alla Mironenko; Andriy Goy; Ihor Kravchuk; Larysa Radchenko; Ludmyla Bolotova; Nataliia Teteriuk
EPI_ISL_732955	Department of Tropical Parasitology	Laboratory of Recombinant Vaccines	Boguslaw Szewczyk; Kirsi Aaltonen; Lukasz Rabalski; Maciej Grzybek; Maciej Kosinski; Ravi Kant; Tarja Sironen; Teemu Smura
EPI_ISL_481513, EPI_ISL_481535, EPI_ISL_756291, EPI_ISL_757337, EPI_ISL_759935, EPI_ISL_995839	Department of Virology and Immunology, University of Helsinki and Helsinki University Hospital, Huslab Finland	Department of Virology, Faculty of Medicine, University of Helsinki, Helsinki, Finland	Essi Korhonen; Hanna Jarva; Hanna Liimatainen; Hannimari Kallio-Kokko; Harri Kangas; Hussein Alburkat; Jenni Virtanen; Maija Lappalainen; Maija Suvanto; Olli Vapalahti; Pekka Ellonen; Phuoc Truong; Ravi Kant; Sari Hannula; Satu Kurkela; Teemu Smura
EPI_ISL_468163	Department of Virology, Public Health Laboratories Division, National Institute of Health	Department of Virology, Public Health Laboratories Division, National Institute of Health	Aamer Ikram; Adnan Khurshid; John Klena; Massab Umair; Muhammad Salman; Nazish Badar; Shannon Whitmer
EPI_ISL_1065271	Department of Virus and Microbiological Special Diagnostics, Statens Serum Institut, Copenhagen, Denmark	Aalborg University	Danish Covid-19 Genome Consortium
EPI_ISL_670141, EPI_ISL_748612	Department of Virus and Microbiological Special Diagnostics, Statens Serum Institut, Copenhagen, Denmark	Albertsen Lab, Department of Chemistry and Bioscience, Aalborg University, Denmark	Danish Covid-19 Genome Consortium
EPI_ISL_436989	Department of Virus and Microbiological Special Diagnostics, Statens Serum Institut, Copenhagen, Denmark, Artillerivej 5, 2300 Copenahgen S	Albertsen lab, Department of Chemistry and Bioscience, Aalborg University, Denmark	Rasmus Kirkegaard
EPI_ISL_614464, EPI_ISL_614746, EPI_ISL_616897, EPI_ISL_617736, EPI_ISL_618119, EPI_ISL_619398, EPI_ISL_622647			
see above	Department of Virus and Microbiological Special Diagnostics, Statens Serum Institut, Denmark	Albertsen lab, Department of Chemistry and Bioscience, Aalborg University, Denmark	Danish Covid-19 Genome Consortia
EPI_ISL_449791, EPI_ISL_449792	Dept. of Medical Microbiology, Stavanger University Hospital, Helse Stavanger HF	Norwegian Institute of Public Health, Department of Virology	Hilde Elshaug; Kamilla Heddeland Instefjord; Karoline Bragstad; Kathrine Stene-Johansen; Olav Hungnes; Rasmus Riis Kopperud
EPI_ISL_666604	Dept. of Microbiology and Infection Control, Akershus University Hospital HF	Dept. of Microbiology and Infection Control, Akershus University Hospital HF	Alexander Hesselberg Løvestad; Hege Vangstein Aamot; Nina Handal; Ole Herman Ambur; Silje Bakken Jørgensen
EPI_ISL_754241	Dinkes Tasikmalaya	"School of Life Sciences and Technology & School of Pharmacy-Institut Teknologi Bandung; Molecular Genetics Laboratory-Faculty of Medicine-Universitas Padjadjaran; Laboratorium Kesehatan Provinsi Jawa Barat"	Agung Eru Wibowo; Azzania Fibriani; Catur Riani; Cut Nur Cinthia Alamanda; Ema Rahmawati; Hammam Riza; Hesti Lina Wiraswati; Husna Nugrahapraja; Irvan Faizal; Karimatu Khoirunnisa; Lia Faridah; Marselina Irasonia Tan; Miftahul Faridl; Rifky Waluyajati Rachman; Ryan Bayusantika Ristandi; Savira Ekawardhani; Soni Solistia Wirawan; Tarwadi; Yunia Sribudiani
EPI_ISL_722856, EPI_ISL_722872, EPI_ISL_722896	Dipartimento di Scienze Biomediche e Oncologia Umana - Azienda Ospedaliero Universitaria Consorziale Policlinico	Istituto Zooprofilattico Sperimentale della Puglia e della Basilicata	Bianco A.; Capozzi L.; Chironna M.; Del Sambro L.; Loconsole D.; Parisi A.
EPI_ISL_422759, EPI_ISL_574781, EPI_ISL_632548, EPI_ISL_632774, EPI_ISL_722594, EPI_ISL_763207	Dutch COVID-19 response team	Erasmus Medical Center	Anne van der Linden; Annemiek van der Eijk; Aura Timen; Bas Oude Munnink; Claudia Schapendonk; Corien Swaan; Corine GeurtsvanKessel; David Nieuwenhuijse; Emmanuelle Munger; Irina Chestakova; Jeroen van Kampen; Jolanda Voermans; Madelief Mollers; Manon Haverkate; Marion Koopmans; Marjan Boter; Mark Pronk; Mart Stein; Pascal Lexmond; Reina Sikkema; Richard Molenkamp; Sandra Kengne Kamga Mobou; Stefan van Nieuwkoop; Theo Bestebroer; on behalf of the Dutch national COVID-19 response team.
EPI_ISL_547445, EPI_ISL_636504, EPI_ISL_636521, EPI_ISL_1014566, EPI_ISL_1014574, EPI_ISL_1014576, EPI_ISL_1014632, EPI_ISL_1165320, EPI_ISL_1216281, EPI_ISL_1232326, EPI_ISL_1232327			
see above	Dutch COVID-19 response team	National Institute for Public Health and the Environment (RIVM)	Adam Meijer; AnneMarie van den Brandt; Bas van der Veer; Chantal Reusken; Dennis Schmitz; Dirk Eggink; Florian Zwagemaker; Harry Vennema; Jeroen Cremer; Sharon van den Brink; on behalf of the national COVID-19 response team
EPI_ISL_450518, EPI_ISL_512314, EPI_ISL_770057	E. Gulbja Laboratorija	Latvian Biomedical Research and Study Centre	Dmitrijs Perminovs; Ivars Silamielis; Jnis Kloviš; Jnis Pjalkovskis; Kaspars Megnis; Mikus Gavars; Monta Ustinova; Uga Dumpis; Vita Rovte; ikita Zrelovs
EPI_ISL_1312631, EPI_ISL_1312683, EPI_ISL_1321971	E. Gulbja laboratorija	Latvian Biomedical Research and Study Centre	Davids Fridmanis; Dmitrijs Perminovs; Elina Dimina; Guntars Zarins; Ivars Silamikelis; Janis Klovins; Janis Pjalkovskis; Jurijs Perevoscikovs; Kaspars Megnis; Laila Silamikele; Lauma Freimane; Laura Ansone; Liga Birzniece; Mikus Gavars; Monta Ustinova; Nikita Zrelovs; Uga Dumpis; Una Krumina; Vita Rovite
EPI_ISL_468750	Facultad de Medicina UC	Center for Mathematical Modeling and Center for Genome Regulation. Santiago, Chile	Allende ML; Ferres M.; Gaete A; González M; Maass A; Palma R; Travisany D; Urra C; Varas M
EPI_ISL_1273102	Faculty of Medicine, Al-Quds University	Faculty of Medicine, Al-Quds University	Al-Jawabreh, A.; Dumaidi, K.; Ereqat, S.; Nasereddin, A.
EPI_ISL_775325, EPI_ISL_964959	Foerde Hospital, Department of Microbiology	Norwegian Institute of Public Health, Department of Virology	Atiya R Ali; Hilde Elshaug; Hilde Vollan; Ignacio Garcia Llorente; Kamilla Heddeland Instefjord; Karoline Bragstad; Kathrine Stene-Johansen; Marie Paulsen Madsen; Olav Hungnes; Rasmus Riis Kopperud; Serina B Engebretsen
EPI_ISL_476139	Folkhalsomyndigheten	The Public Health Agency of Sweden	Anna Risberg; Anna-Malin Linde; Karin Tegmark-Wisell; Maria Lind Karlberg; Mattias Haukland; Olov Svartstrom; Oskar Karlsson Lindsjo; Petra Edquist; Reza Advani; Sandra Broddesson; Shamam Muradrasoli
EPI_ISL_581487	Fondation Congolaise pour la recherche medicale (FCRM)	NGS Competence Center Tübingen, Institut für Medizinische Mikrobiologie und Hygiene, Universitätsklinikum Tübingen	Angel Angelov

EPI_ISL_912376	Fondation Congolaise pour la recherche medicale (FCRM), Francine Ntoumi	NGS Competence Center Tuebingen, Institut für Medizinische Mikrobiologie und Hygiene, Universitaetsklinikum Tübingen	Angel Angelov
EPI_ISL_522549	Félix Guyon Hospital	UMR PIMIT Université de La Réunion	Camille Lebarbenchon; David Wilkinson; Patrick Mavingui
EPI_ISL_1265757, EPI_ISL_1265824	GHE REUNION	CNR Virus des Infections Respiratoires - France SUD	Antonin Bal; Bruno Lina; Gregory Destras; Gwendolyne Burfin; Hadrien Regue; Laurence Josset; Martine Valette; Quentin Semanas
EPI_ISL_406798	General Hospital of Central Theater Command of People's Liberation Army of China	BGI & Institute of Microbiology, Chinese Academy of Sciences & Shandong First Medical University & Shandong Academy of Medical Sciences & General Hospital of Central Theater Command of People's Liberation Army of China	Weifeng Shi and Zhenhong Hu; Weijun Chen; Yuhai Bi
EPI_ISL_746651, EPI_ISL_746697, EPI_ISL_1167713, EPI_ISL_1167771	Genetica Molecular and Subdepartamento de Virologia ISP Chile	Instituto de Salud Publica de Chile	Andres Castillo; Barbara Parra; Gisselle Barra; Jaime Lagos; Javier Tognarelli; Jorge Fernandez; Karen Orostica; Loredana Arata; Patricia Bustos; Rodrigo Fasce
EPI_ISL_735353, EPI_ISL_735435	Genomic Laboratory (GLAB) (Conjoint lab of Health Directorate of Istanbul and Istanbul Technical University)	Genomic Laboratory (GLAB), Istanbul Technical University	Arzu Irvem; Ayse Serra Ozel; Betsi Kose; Gizem Alkurt; Gizem Dinler Doganay; Ilker Karacan; Jale Yildiz; Levent Doganay; Mehtap Aydin; Nihat Bugra Agaoglu; Nilsun Altunal; Nisan Denizce Can; Ozlem Akgun Dogan; Payam Zolfagharian; Tugba Kizilboga Akgun; Yasemin Kendir Demirkol
EPI_ISL_496615, EPI_ISL_496616, EPI_ISL_496693, EPI_ISL_1225470, EPI_ISL_1225546, EPI_ISL_1225574, EPI_ISL_1225580			
see above	Gorgas Memorial Laboratory of Health Studies	Gorgas Memorial Laboratory of Health Studies	Adriana Weeden; Alejandra Valoy; Alexander A Martinez; Alexander Martinez; Ambar Moreno; Anyuri Ortiz; Brechla Moreno; Claudia Gonzalez; Claudia Gonzalez Sandra Lopez-Verges; Daniel Castillo; Danilo Franco; Davis Beltran; Dimelza Arauz; Elimelec Valdespino; Gretel Vasquez; Ilka Guerra; Isela Guerrero; Jessica Gondola; Jim Chang; Juan Miguel Pascale; Layda Abrego; Lisseth Saenz; Mabel Martinez-Montero; Maria Chen-German; Marlene Castillo; Melissa Gaitan; Oris Chavarria; Rita Corrales; Rita Rodriguez; Sandra Lopez-Verges; Yamilka Diaz; Yaneth Pitti; Zumara Chaverra
EPI_ISL_906306, EPI_ISL_1001457	Gorgas memorial Institute For Health Studies	Gorgas memorial Institute For Health Studies	Abrego L; Arauz D; Castillo D; Castillo M; Chavarria O; Diaz Y; Díaz Y; Franco D; Gaitan M; Gondola J; Gondola Y; González C; Lopez-Verges S.; Lopez-Verguez S; Martinez AA; Martinez Alexander A.; Moreno A; Moreno B; Pitti Y; Saenz L
EPI_ISL_1001458	Gorgas memorial Institute for Health Studies	Gorgas memorial Institute for Health Studies	Arauz D; Castillo D; Castillo M; Chavarria O; Díaz Y; Franco D; Gaitan M; Gondola J; González C; Lopez-Verges S.; Martinez AA; Moreno A; Moreno B; Pitti Y; Saenz L
EPI_ISL_1159385	Graha Medika Maternity Hospital	Institute of Tropical Disease, Universitas Airlangga	Aldise M Nastri; Diah R Kusumawati; Gatot Soegiarto; Jezzy R Dewantari; Kazufumi Shimizu; Krisnoadi Rahardjo; Laksmi Wulandari; Maria I Lusida; Resti Yudhawati; Rima R Prasetya; Soetjipto; Yasuko Mori
EPI_ISL_447748	Grupo de Investigaciones Microbiológicas-UR (GIMUR), Departamento de Biología, Facultad de Ciencias Naturales, Universidad del Rosario, Bogotá, Colombia	Grupo de Investigaciones Microbiológicas-UR (GIMUR), Departamento de Biología, Facultad de Ciencias Naturales, Universidad del Rosario, Bogotá, Colombia Instituto Nacional de Salud, Bogotá, Colombia Icahn School of Medicine at Mount Sinai, New York, USA	Adriana Castillo; Alberto Paniz-Mondolfi; Ana S. Gonzalez-Reiche; Angelica Rico; Aníbal A. Teherán; Carolina Florez; Carolina Hernandez; David Martínez; Emilia Mia Sordillo; Esther C. Barros; Harm van Bakel; Jesús E. Jaimes; Juan David Ramírez; Laura Vega; Lisseth Pardo; Marina Muñoz; Martha L. Ospina; Matthew M. Hernandez; Nathalia Ballesteros; Sergio Castañeda; Sergio Gomez; Viviana Simon
EPI_ISL_1273048, EPI_ISL_1273071	Guam Public Health Laboratory	Centers for Disease Control and Prevention Division of Viral Diseases, Pathogen Discovery	Anna Montmayeur; Anna Uehara; Ben L. Rambo-Martin; Clinton R. Paden; Dhwani Batra; Haibin Wang; Jasmine Padilla; Jing Zhang; Justin Lee; Katie Dillon; Krista Queen; Kristen Knipe; Kristine Lacek; Lori Rowe; Mark Burroughs; Matthew Schmerer; Mili Sheth; Peter W. Cook; Rachel Marine; Sam Shepard; Sarah Nobles; Shoshona Le; Suxiang Tong; Yan Li; Ying Tao
EPI_ISL_428466	Guangdong Provincial Center for Diseases Control and Prevention;Guangdong Provincial Institute of Public Health	School of Public Health, The University of Hong Kong	Bosheng Li; Hanri Zeng; Haogao Gu; Hui-Ling Yen; Jie Wu; Leo L.M. Poon; Lijun Liang; Tie Song; Yao Hu; Yingchao Song; Zhencui Li
EPI_ISL_596269	HELIX LCC	WHO National Influenza Centre Russian Federation	Andrey Komissarov; Anna Ivanova; Artem Fadeev; Daria Danilenko; Dmitry Bazhenov; Kseniya Komissarova
EPI_ISL_602396, EPI_ISL_733038, EPI_ISL_733368, EPI_ISL_1372376	HELIX LLC	WHO National Influenza Centre Russian Federation	Andrey Komissarov; Anna Ivanova; Artem Fadeev; Daria Danilenko; Dmitry Bazhenov; Dmitry Lioznov; Elena Nabieva; Georgii Bazykin; Ksenia Safina; Kseniya Komissarova; Maria Pisareva; Maria Timofeeva; Tamila Musaeva; Veronika Eder
EPI_ISL_1170956	HIV Molecular Lab, Ethiopian Public Health Institute	HIV Molecular Lab	A.G.; Abichu, G.; Ahyong, V.; D. and Tato, C.; Damena, D.; Detweiler, A.; Fahsbender, E.; Gutema, G.; Kalantar, K.; Kidane, E.; Leta; Neff, N.; S.K.; Tan, M.; Tessema; Vanaerschot, M.; Weldemariam
EPI_ISL_1360026, EPI_ISL_1360028	HOPITAL UNIVERSITAIRE DE FORT DE FRANCE	CNR Virus des Infections Respiratoires - France SUD	Antonin Bal; Bruno Lina; Gregory Destras; Gwendolyne Burfin; Hadrien Regue; Laurence Josset; Martine Valette; Quentin Semanas
EPI_ISL_487370, EPI_ISL_501242	Hellenic Pasteur Institute, National Influenza Reference laboratory of Southern Greece & Unit of Bioinformatics and Applied Genomics	Hellenic Pasteur Institute, National Influenza Reference laboratory of Southern Greece & Unit of Bioinformatics and Applied Genomics	Andreas Mentis; Androniki Voulgari-Kokota; Antonios Kalliaropoulos; Aspasia Kontou; Athanasios Kossyvakis; Evangelidou Maria; Horefti Elina; Timokratis Karamitros; Vasiliki Pogka
EPI_ISL_430469	Hellenic Pasteur Institute, Public Health Laboratories	Hellenic Pasteur Institute, Public Health Laboratories, Unit of Bioinformatics and Applied Genomics	Andreas Mentis; Androniki Voulgari-Kokota; Antonios Kalliaropoulos; Aspasia Kontou; Athanasios Kossyvakis; Evangelidou Maria; Horefti Elina; Timokratis Karamitros; Vasiliki Pogka
EPI_ISL_451649	Hematology Laboratory, Section of Molecular Diagnostics, University Clinical Centre, Medical University of Gdansk	Laboratory of Recombinant Vaccines	Adam Sodol; Aneta Szulc; Boguslaw Szewczyk; Ewa Milosz; Krystyna Bienkowska-Szewczyk; Krzysztof Lewandowski; Lukasz Rabalski; Marlena Robakowska
EPI_ISL_700089	Hematopathology Laboratory, ACTREC, TMC	Hematopathology Laboratory, ACTREC, TMC	ACTREC; Hematopathology Laboratory
EPI_ISL_1166933, EPI_ISL_1166949	Hopital	National Reference Center for Viruses of Respiratory Infections, Institut Pasteur, Paris	Angela Brisebarre; Camille Capel; Combe Patrice; Etienne Simon-Lorière; Marion Barbet; Maud Vanpeene; Méline Bizard; Sylvie Behillil; Sylvie van der Werf; Vincent Enouf
EPI_ISL_1381164	Hospital	National Reference Center for Viruses of Respiratory Infections, Institut Pasteur, Paris	Angela Brisebarre; Bastian Sylvaine; Camille Capel; Etienne Simon-Lorière; Louise Lefrançois; Marion Barbet; Maud Vanpeene; Méline Bizard; Sylvie Behillil; Sylvie van der Werf; Vincent Enouf
EPI_ISL_1196864	Hospital Alor Gajah	Institute for Medical Research, Infectious Disease Research Centre, National Institutes of Health, Ministry of Health Malaysia	Kamel K; Mohd Zawawi Z; Ramly N; Robert F; Suppiah J; Thayan R
EPI_ISL_654046, EPI_ISL_654082, EPI_ISL_654129, EPI_ISL_654150, EPI_ISL_780094	Hospital General Universitario Gregorio Marañón	SeqCOVID-SPAIN consortium/IBV(CSIC)	Darío García de Viedma; Jon Sicilia; Julia Suárez; Laura Pérez-Lago; Marta Herranz; Patricia Muñoz and SeqCOVID-SPAIN consortium; Pilar Catalán
EPI_ISL_412964	Hospital Israelita Albert Einstein	Instituto Adolfo Lutz Interdisciplinary Procedures Center Strategic Laboratory	Andrew Rambaut; Claudia Regina Gonçalves; Claudio Tavares Sacchi; Daniela Bernardes Borges da Silva; Ester Cerdeira Sabino; Flávia Cristina da Silva Sales; Ingra Morales Claro; Jaqueline Goes de Jesus; Joshua Quick; Maria do Carmo; Nicholas James Loman; Nuno Rodrigues Faria; Sampaio Tavares Timenetsky
EPI_ISL_831144	Hospital Universitario La Paz (Madrid)	SeqCOVID-SPAIN consortium/IBV(CSIC)	Elias Dahdouh; Fernando Lázaro-Perona; Jesús Mingorance and SeqCOVID-SPAIN consortium; María Rodríguez-Tejedor
EPI_ISL_530117	Hospital Universitario Ramón y Cajal	Hospital Universitario La Paz	Elias Dahdouh; Esther Viedma; Fernando Lázaro; Jesús Mingorance; Juan Carlos Galán; Julio García; María Rodríguez; Mª Dolores Folgueira; Natalia Stella; Rafael Cantón; Rafael Delgado; Raúl Recio; Sara González
EPI_ISL_789887	Houston Methodist Hospital	Houston Methodist Hospital	David W. Bernard; Heather Hendrickson; James J. Davis; Layne Pruitt; Marcus Nguyen; Matthew Ojeda Saavedra; Maulik Shukla; Paul A. Christensen; Prasanti Yerramilli; Randall J. Olsen; S. Wesley Long; Sishir Subedi; and James M. Musser
EPI_ISL_645080	Human Genome Variation Research Group, Malopolska Centre of Biotechnology	Human Genome Variation Research Group, Malopolska Centre of Biotechnology	Botwina, P.; Branicki, W.; Dabrowska, A.; Foremny, J.; Gromowski, T.; Klajmon, A.; Kopera, K.; Kowalski, M.; Labaj; Marszalek, K.; Owczarek, K.; P.P.; Pisarek, A.; Pospiech, E.; Pyrc, K.; Sanak, M.; Swadzba, J.; Szczepanski, A.
EPI_ISL_526222	Hungarian Defence Forces Military Medical Centre	National Laboratory of Virology, Szentágothai Research Centre	Balázs Somogyi; Bálint Eszenyi; Endre Gábor Tóth; Ferenc Jakab; Gábor Kemenesi
EPI_ISL_833197	Hôpital Bichat Claude Bernard, Laboratoire de Virologie	IAME UMR1137 Inserm, Université de Paris, Hôpital Bichat	Alexandre Storto; Amélie Recoing; Antoine Bridier; Benoit Visseaux; Charlotte Charpentier; Diane Descamps; Gilles Collin; Lena Daniel; Mélanie Bertine; Nadhira Houhou-Fidouh; Quentin Le Hingrat; Siham Hamri

EPI_ISL_653781, EPI_ISL_653797, EPI_ISL_653806, EPI_ISL_653807, EPI_ISL_653823, EPI_ISL_722851, EPI_ISL_722865, EPI_ISL_722867, EPI_ISL_722871			
see above	I.R.C.C.S. "S. De Bellis" - Ente Ospedaliero	Istituto Zooprofilattico Sperimentale della Puglia e della Basilicata	Bianco A.; Capozzi L.; Cipolletta D.; Del Sambro L.; Galante D.; Lippolis A.; Manzulli V; Notarnicola M.; Pace L.; Parisi A.; Rondinone V.
EPI_ISL_590698, EPI_ISL_603137, EPI_ISL_603139, EPI_ISL_603173, EPI_ISL_603178, EPI_ISL_603187, EPI_ISL_609991, EPI_ISL_1091277			
see above	INMI Lazzaro Spallanzani IRCCS	INMI Lazzaro Spallanzani IRCCS	A Di Caro; Antonino Di Caro; B Bartolini; Barbara Bartolini; Beatrice Valli; C.E.M Gruber; CEM Gruber; Cesare E.M. Gruber; E Giombini; Eleonora Lalle; Emanuela Giombini; F Messina; Francesco Messina; Francesco Vairo; Giuseppina Cappiello; M Rueca; MR Capobianchi; Maria R. Capobianchi; Martina Rueca; O Butera; Simone Lanini
EPI_ISL_410545, EPI_ISL_410546	INMI Lazzaro Spallanzani IRCCS	Laboratory of Virology, INMI Lazzaro Spallanzani IRCCS	Barbara Bartolini; Cesare E. M. Gruber; Concetta Castilletti; Eleonora Lalle; Emanuela Giombini; Emanuele Nicastri; Fabrizio Carletti; Francesca Colavita; Francesco Messina; Giuseppe Ippolito.; Maria R. Capobianchi; Martina Rueca
EPI_ISL_826830	INSPI-CRN DE INFLUENZA Y OTROS VIRUS RESPIRATORIOS	Instituto de Salud Publica de Chile	Alfredo Bruno; Andres Castillo; Barbara Parra; Domenica de Mora; Gisselle Barra; Jaime Lagos; Javier Tognarelli; Jimmy Garcez; Jorge Fernandez; Loredana Arata; Manuel Gonzalez; Martiza Olmedo; Michelle Paez; Patricia Bustos; Rodrigo Fasce; Solon Narvaez
EPI_ISL_775261	INT Fondazione Pascale	INT Fondazione Pascale	INT Fondazione Pascale
EPI_ISL_751318, EPI_ISL_751325, EPI_ISL_751340, EPI_ISL_751341, EPI_ISL_751343, EPI_ISL_751384, EPI_ISL_751386, EPI_ISL_751390, EPI_ISL_751417, EPI_ISL_751428, EPI_ISL_751437			
see above	IRCCS Sacro Cuore Don Calabria Hospital, Department of Infectious, Tropical Diseases & Microbiology	University of Verona, Department of Biotechnology	Antonio Mori; Chiara Degli Esposti; Chiara Piubelli; Cristina Beltrami; Elena Pomari; Emanuela Cosentino; Giulia Lopatriello; Luca Marcolungo; Massimo Delledonne; Michela Deiana
EPI_ISL_572323	IZSM	IZSM	Giovanna Fusco; Lorena Cardillo; Maurizio Viscardi
EPI_ISL_900647, EPI_ISL_1086066, EPI_ISL_1086075, EPI_ISL_1086114, EPI_ISL_1390494, EPI_ISL_1390525	IZSM	TIGEM	Andrea Ballabio; Anna Manfredi; Antonio Grimaldi; Antonio Limone; Antonio Limone Luigi Atripaldi Pellegrino Cerino; Biancamaria Pierri; Biancamaria Pierri Claudia Tiberio Valentina Bouche; Chiara Colantuono; Davide Cacchiarelli; Davide Cacchiarelli.; Denise Di Concilio; Francesco Panariello; Lucio Di Filippo; Marcello Salvi; Maria Concetta Cuomo; Patrizia Annunziata; Pellegrino Cerino; Valentina Bouche
EPI_ISL_738131	IZSM-U.O.C. Virologia	Istituto Zooprofilattico Sperimentale del Mezzogiorno	Giovanna Fusco; Lorena Cardillo; Maurizio Viscardi
EPI_ISL_1018088	Immunology, Noguchi Memorial Institute for Medical Research	Immunology, Noguchi Memorial Institute for Medical Research	Adu, B.; Adusei-Poku; Agbodzi, B.; Ampofo; Appiah-Kubi, J.; Asare; Bonney; Egyir, B.; J.K.; K.M.; Kumordjie, S.; M.A.; Mohktar, Q.; Odoom; Oteng, F.; Owusu-Nyantakyi, C.; W.K.; Yeboah, C.
EPI_ISL_413523	Indian Council of Medical Research-National Institute of Virology	National Influenza Center, Indian Council of Medical Research-National Institute of Virology	Choudhary ML; Potdar V; Shete-Aich A; Yadav PD
EPI_ISL_498230, EPI_ISL_498252	Institut Pasteur de Dakar	Institut Pasteur de Dakar	Amadou Alpha Sall.; Mamadou Diop; Mamadou Malado Jallow; Marie Henriette Dior Ndione; Moussa Moise Diagne; Ndongo Dia; Ousmane Faye; Safietou Sankhe Mbengue
EPI_ISL_1013418, EPI_ISL_1013431	Institut Pasteur de Guadeloupe	National Reference Center for Viruses of Respiratory Infections, Institut Pasteur, Paris	Angela Brisebarre; Camille Capel; Etienne Simon-Lorière; Marion Barbet; Maud Vanpeene; Méline Bizard; Sylvie Behillil; Sylvie van der Werf; Talarmin Antoine; Vincent Enouf
EPI_ISL_455792	Institute for Medical Research, Infectious Disease Research Centre, National Institutes of Health, Ministry of Health Malaysia	Malaysia Genome Institute	Azrin Ahmad; Enizza Kasim; Irni Suhayu Sapian; Jeyanthi Suppiah; Mohd Faizal Abu Bakar; Mohd Noor Mat Isa; Norazah Ahmad; Nurhezreen Md Iqbal; Ravindran Thayan; Shahrul Hisham Zainal Ariffin; Shamsidar Sopie; Siti Noraini Othman; Tahir Aris; Yusuf Muhammad Noor; Zarina Mohd Zawawi
EPI_ISL_718275, EPI_ISL_718279, EPI_ISL_718314, EPI_ISL_944096, EPI_ISL_968089, EPI_ISL_1081350	Institute for Medical Research, Infectious Disease Research Centre, National Institutes of Health, Ministry of Health Malaysia	Institute for Medical Research, Infectious Disease Research Centre, National Institutes of Health, Ministry of Health Malaysia	Azizan MA; Kamel K; Mohd-Zawawi Z; Suppiah J; Thayan R
EPI_ISL_812951	Institute for Urban Disease Control and Prevention	COVID-19 Network Investigations (CONI) Alliance	Amornmas Kongklieng; Anek Mungaomklang; Angkana Huang; Anthony R. Jones; Arporn Wangwiwatsin; Bhakbhoom Panthan; Chonticha Klungtong; Duangkamon Loesbanluechai; Ekawat Pasomsub; Elizabeth Batty; Insee Sensorn; Janjira Thaipadungpanit; Kamolthip Atsawawaranunt; Khajohn Joonsalak; Kingkan Rakmanee; Krittikorn Kumpornsin; Namfon Kotanan; Prayuth Kaewmalang; Pukkaporn Parnwijitkul; Stefan Fernandez; Thanat Chookajorn; Theerarat Kochakarn; Treewat Watthanachockchai; Wasun Chantratita; Wudtichai Manasatienkij
EPI_ISL_1055779	Institute of Biocides and Medical Ecology, Belgarde, Serbia	Virology Department Institute of Microbiology and Immunology Faculty of Medicine University of Belgrade	Abazovic Dzihan; Banko Ana; Despot Dragana; Loncar Ana; Milicevic Ognjen; Miljanovic Danijela
EPI_ISL_582032	Institute of Human Genetics, Polish Academy of Sciences	Institute of Human Genetics, Polish Academy of Sciences	Adam Ustaszewski; Andrzej Pawski; Emilia Lis; Ewa Zitkiewicz; Marta Kaczmarek-Ry; Micha Witt; Szymon Hryhorowicz
EPI_ISL_1098797, EPI_ISL_1181830	Institute of Microbiology and Immunology, Faculty of Medicine, University of Ljubljana	Institute of Microbiology and Immunology, Faculty of Medicine, University of Ljubljana	Alen Sulji; Andrej Celar; Dominika Šturm; Doroteja Vlaj; Mario Poljak; Matic Brvar; Miša Korva; Patricija Pozvek; Samo Zakotnik; Tatjana Avši - Županc; Tomaž Mark Zorec; Špela Pleh
EPI_ISL_548942	Institute of Microbiology, University of Veterinary and Animal sciences	Institute of Microbiology, University of Veterinary and Animal sciences	Ali; Altaf, I.; Anwar, M.; Ashraf; Asif, A.; Attique; Awan; Aziz; Bhatti; Cheema; Fazal, S.; Hassan, S.; Khan; Khan, N.; M.A.; M.B.; M.M.; M.S.; M.T.; M.U.; M.W.; M.Z.; Mehmood, A.; Mukhtar, N.; N.A.; Nawaz, M.; Rafique, S.; Rana; Raza, S.; S.Q.; S.Z.; Sardar, N.; Sarwar, H.; Shabbir; Shah; Tahir, Z.; Yaqub, T.; Younis
EPI_ISL_477016, EPI_ISL_486843, EPI_ISL_527814, EPI_ISL_697791	Institute of Microbiology, Universidad San Francisco de Quito	Institute of Microbiology, Universidad San Francisco de Quito	Belén Prado-Vivar; Bernardo Gutiérrez; Carla Torres; Fausto Maldonado; Gabriel Trueba; Geovanny Carzola; Jonathan Araujo; Jorge Luis Velez; Juan José Guadalupe; Khurram Mahbbob; Michelle Grunauer; Monica Becerra-Wong; Patricio Rojas-Silva; Paúl Cárdenas; Sully Márquez; Verónica Barragán
EPI_ISL_403929	Institute of Pathogen Biology, Chinese Academy of Medical Sciences & Peking Union Medical College	Institute of Pathogen Biology, Chinese Academy of Medical Sciences & Peking Union Medical College	Chao Wu; Jianwei Wang; Lili Ren; Qi Jin; Yiwei Liu; Zhiqiang Wu; Zichun Xiang
EPI_ISL_577734, EPI_ISL_718251, EPI_ISL_875538, EPI_ISL_959645, EPI_ISL_1234435	Institute of Virology, Biomedical Research Center of the Slovak Academy of Sciences, Bratislava	Faculty of Natural Sciences, Comenius University, Bratislava	Boris Klempa; Broa Brejová; Dominika Friová; Edita Staroová; Elena Tichá; Jozef Nosek; Juraj Kopáek; Kristína Boršová; Martina Liková; Martina Neboháová; Monika Sláviková; Sabina Fumaová Havlíková; Tomáš Vina; Viktória Hodorová; Viktória abanová; ubomíra Lukáiková
EPI_ISL_417878	Institute of Virology, Biomedical Research Center of the Slovak Academy of Sciences, Bratislava; Public Health Authority of the Slovak Republic, Bratislava	Institute of Virology, Biomedical Research Center of the Slovak Academy of Sciences, Bratislava; Comenius University Science Park, Bratislava	Boris Klempa; Diana Rusáková; Edita Staroová; Elena Tichá; Jaroslav Budiš; Juraj Kopáek; Juraj Koi; Martina Liková; Miroslav Böhmer; Monika Slávikova; Sabina Fumaová Havlíková; Tomáš Szemeš; Werner Krampl
EPI_ISL_1017695	Institute of Virology, Vaccines and Sera "Torlak"	Institute of microbiology and Immunology, Faculty of Medicine, University of Belgrade	Jankovic, M.; Jovanovic, T.; Knezevic, A.; Milicevic, O.; Sekler, M.; Tesovic, B.; Vidanovic, D.
EPI_ISL_491287	Instituto Gulbenkian de Ciência	Instituto Gulbenkian de Ciência	Cathy Paulino; Joao Sobral; João Costa; Ricardo Leite; Susana Ladeiro
EPI_ISL_491944, EPI_ISL_491947, EPI_ISL_491951	Instituto Nacional de Investigación en Salud Pública - INSPI	INSPI - Charité	Alberto Orlando; Alexandra Usiña; Alfredo Bruno Caicedo; Andres Moreira-Soto; Anna-Lena Sander; Denisses Portugal; Domenica de Mora Coloma; Jan Felix Drexler; Juan Carlos Zeballos; Manuel Gonzalez; Maritza Olmedo; Nina Krause; Silvia Salgado
EPI_ISL_1315515	Instituto Nacional de Medicina Genomica	Instituto Nacional de Medicina Genomica	Alcaraz N; Arriaga-Canon C; Cedro-Tanda A; Frías-Jimenez E; Garcia-Cárdenas FJ; Gonzalez-Barrera D; González-Woge MA; Herrera-Montalvo LA; Hidalgo-Miranda A; Mendoza-Vargas A; Munguia-Garza; Ramirez-Vega O; Rangel-DeLeon D; Reyes-Grajeda JP
EPI_ISL_536478, EPI_ISL_536518	Instituto Nacional de Salud	Laboratorio de Infecciones Respiratorias Agudas	David Tarazona; Dennis Carhuaricra; Eduardo Juscamayta Lopez; Faviola Valdivia Guerrero; Lenin Maturrano Hernandez; Nancy Rojas Serrano; Ronnie Gavilan Chavez
EPI_ISL_456129	Instituto Nacional de Salud - Unidad de Secuenciación y Análisis Genómico	Instituto Nacional de Salud, Universidad Cooperativa de Colombia, Instituto Alexander von Humboldt, Imperial College-London, London School of Hygiene & Tropical Medicine	Astrid C. Flórez; Carlos Franco-Muñoz; Christian Julian VillabonaArenas; Diana Marcela Walteros-Acero; Diego A. Álvarez-Díaz; Erika Ospitia; Gloria Puerto; Jose A. Usme-Ciro; Juliana Barbosa; Katherine Laiton-Donato; Liz Villabona-Arenas; Luz Dary Rodriguez; Mailyn A.Gonzalez; Marcela Mercado-Reyes.; Martha Lucia Ospina Martinez; Nicolas D. Franco-Sierra; Sergio Gomez-Rangel; Sussy Echeverria; Zulma M. Cucunubá
EPI_ISL_447763	Instituto Nacional de Salud, Bogotá, Colombia	Grupo de Investigaciones Microbiológicas-UR (GIMUR), Departamento de Biología, Facultad de Ciencias Naturales,	Adriana Castillo; Alberto Paniz-Mondolfi; Ana S. Gonzalez-Reiche; Angelica Rico; Aníbal A. Teherán; Carolina Florez; Carolina Hernandez; David Martínez; Emilia Mia Sordillo; Esther C. Barros; Harm van Bakel; Jesús E. Jaimes; Juan David Ramírez; Laura Vega; Lisseth Pardo; Marina Muñoz;

		Universidad del Rosario, Bogotá, Colombia Instituto Nacional de Salud, Bogotá, Colombia Icahn School of Medicine at Mount Sinai, New York, USA	Martha L. Ospina; Matthew M. Hernandez; Nathalia Ballesteros; Sergio Castañeda; Sergio Gomez; Viviana Simon
EPI_ISL_739674	Instituto Nacional de Salud, Bogotá, Colombia	Instituto Nacional de Salud, Bogotá, Colombia	Carlos Franco-Muñoz; Diego A. Álvarez-Díaz; Diego Andrés Prada; Jonathan Reales; Katherine Laiton-Donato; Magdalena Weisner; Marcela Mercado-Reyes; Martha Lucia Ospina Martinez; Mauricio Pacheco-Montealegre; Sheryl Corchuelo
EPI_ISL_887420, EPI_ISL_887427, EPI_ISL_887461, EPI_ISL_887503, EPI_ISL_964937	Instituto Nacional de Saude (INS), Mozambique	KRISP, KZN Research Innovation and Sequencing Platform	Emmanuel S; Giandhari J; Nadia Sitoe; Nalia Ismael; Nedio Mabunda; Paulo Arnaldo; Pillay S; Tegally H; Wilkinson E; de Oliveira T
EPI_ISL_511029, EPI_ISL_693581, EPI_ISL_1023463, EPI_ISL_1117190	Instituto Nacional de Saude (INSA)	Instituto Nacional de Saude (INSA)	Borges et al
EPI_ISL_941857, EPI_ISL_1116821	Instituto Nacional de Saude (INSA) and Instituto Gulbenkian de Ciencia (IGC)	Instituto Nacional de Saude (INSA) and Instituto Gulbenkian de Ciencia (IGC)	Borges et al
EPI_ISL_913928, EPI_ISL_913931	Instituto de Diagnostico y Referencia Epidemiologicos INDRE_RNLSP	Instituto de Diagnostico y Referencia Epidemiologicos (INDRE)	Abril Rodriguez-Maldonado; Adnan Araiza-Rodriguez; Claudia Wong-Arambula; David Fragoso-Fonseca; Ernesto Ramirez-Gonzalez.; Fabiola Garces-Ayala; Gisela Barrera-Badillo; Irma Lopez-Martinez; Lucia Hernandez-Rivas; Mayra Jimenez-Morales; Nancy Munoz-Hernandez; Natividad Cruz-Ortiz; Sergio Rangel-Guerrero; Tatiana Nunez-Garcia
EPI_ISL_748141	Instituto de Investigaciones Biologicas Clemente Estable	Institut Pasteur de Montevideo	Ana Carolina Mendonça; Andres Lizasoain; Camila Simoes; Cecilia Alonso; Cecilia Salazar; Daiana Mir; Fernando Lopez-Tort; Fernando Motta; Gonzalo Bello; Ighor Arantes; Ignacio Ferrés; Jose Sotelo; Leticia Maya; Letícia Garay Martins; Luciana Appolinario; Lucia Spangenberg; Mailen Arleo; Mariana Brandes; Marilda Mendonça Siqueira; Marilda Tereza Mar da Rosa; Maria Jose Benitez-Galeano; Martín Graña; Matias Castells; Matias Victoria; Matías Salvo; Natalia Rego; Natalia Reyes; Pablo Smircich; Paola Cristina Resende; Rodney Colina; Tamara Fernandez-Calero; Tania Possi; Tatiana Schäffer Gregianini; Veronica Noya; Yasser Vega
EPI_ISL_1395784	Instituto de Investigaciones Biomédicas en Retrovirus y SIDA (INBIRS)	Área de Secuenciación del Laboratorio de Virología del Hospital de Niños Dr. Ricardo Gutierrez on behalf of 'Proyecto Argentino Interinstitucional de genomica de SARS-CoV-2' (PAIS Consortium)	Acuña; Alexay; D; Federico Remes Lenicov; Goya; Horacio Salomón; LE; Lusso; M; MI; Nabaes Jodar; Natale; S; Valinotto; Vanesa Seery; Viegas, M.
EPI_ISL_1167157	Iressef Genomics lab	L'institut de Recherche en Santé, de Surveillance Épidémiologique et de Formation (IRESSEF)	Abdou PADANE; Abdoulie KANTEH; Abdul Karim SESAY; Ambroise AHOUIDI; Aminata DIA; Aminata MBOUP; Astou Gaye GAYE; Barada CISSE; Birahim Piere NDIAYE; Gora LO; Khadim GUEYE; Moustapha MBOW; Nafisatou LEYE; Ndeye Coumba Toure KANE; Papa Alassane DIAW; Souleymane MBOUP; Yacine DIA
EPI_ISL_956389	Isolation - Virology Unit, Institut Pasteur du Cambodge; Sequencing - US National Institute of Allergy and Infectious Diseases Cambodia, US Naval Medical Research Unit -2, Cambodia National Institute for Public Health	Virology Unit, Institut Pasteur du Cambodge	Chau Darapheak; Chin Savuth; Erik A Karlsson; Jennifer Bohl; Jessica Manning; Jose A Garcia-Rivera; Kraing Sidonn; Ly Sovann; Sophana Chea; Sreyngim Lay; Veasna Duong; Vireak Heang; Yi Sengdoeurn
EPI_ISL_1240648	Israel Central Virology Laboratory	Israel National Consortium for SARS-CoV-2 sequencing	Assaf Rokney; Dana Bar-Ilan; David A. Zeevi; Efrat Dahan Bucris; Efrat Glick-Saar; Efrat Rorman; Ella Mendelson; Ephraim Fass; Eva Nachum; Gal Zizelski Valenci; Gideon Rechavi; Israel Nissan; Joseph Jaffe; Maya Davidovich Cohen; Michal Mandelboim; Mor Rubinstein; Neta Zuckerman; Omer Murik; Omri Nayshool; Oran Erster; Orna Mor; Tzvia Mann
EPI_ISL_516887, EPI_ISL_516894, EPI_ISL_649082, EPI_ISL_649094	Israel Central Virology laboratory	Israel Central Virology laboratory	Efrat Dahan Bucris; Ella Mendelson; Michal Mandelboim; Neta Zuckerman; Oran Erster
EPI_ISL_477201	Istituto Zooprofilattico Sperimentale Puglia e Basilicata;	Beaconlab (Bioinformatics, Evolution and Comparative Genomics lab), Dept of Biosciences, University on Milan	Chiara M.; Manzari C.; Parisi A.; Pesole G.
EPI_ISL_736798, EPI_ISL_736820, EPI_ISL_736830, EPI_ISL_736843, EPI_ISL_736885, EPI_ISL_776910, EPI_ISL_776956, EPI_ISL_776968, EPI_ISL_777000, EPI_ISL_778646, EPI_ISL_778653, EPI_ISL_778664, EPI_ISL_778677, EPI_ISL_778681, EPI_ISL_778709, EPI_ISL_778722, EPI_ISL_778724, EPI_ISL_778728, EPI_ISL_778743, EPI_ISL_778744, EPI_ISL_778750, EPI_ISL_778763, EPI_ISL_778767, EPI_ISL_778773, EPI_ISL_778778, EPI_ISL_778780, EPI_ISL_778781, EPI_ISL_778784, EPI_ISL_778789, EPI_ISL_778793, EPI_ISL_778802, EPI_ISL_778803, EPI_ISL_778813, EPI_ISL_833555, EPI_ISL_833563, EPI_ISL_833566, EPI_ISL_833568, EPI_ISL_833569, EPI_ISL_833570, EPI_ISL_837255, EPI_ISL_837270, EPI_ISL_837280, EPI_ISL_837306, EPI_ISL_837311, EPI_ISL_837312, EPI_ISL_837447, EPI_ISL_837451, EPI_ISL_837467, EPI_ISL_837469, EPI_ISL_837487, EPI_ISL_960459, EPI_ISL_960504, EPI_ISL_960516, EPI_ISL_960519, EPI_ISL_960632, EPI_ISL_960647, EPI_ISL_1307875			
see above	Istituto Zooprofilattico Sperimentale del Mezzogiorno	TIGEM	Andrea Ballabio; Anna Manfredi; Antonio Grimaldi; Antonio Grimaldi Patrizia Annunziata Francesco Panariello Biancamaria Pierri Claudia Tiberio Valentina Bouche Chiara Colantuono Maria Concetta Cuomo Denise Di Concilio Lucio Di Filippo Anna Manfredi Marcello Salvi Antonio Limone Luigi Atripaldi Pellegrino Cerino Andrea Ballabio Davide Cacchiarelli; Antonio Limone; Biancamaria Pierri; Chiara Colantuono; Davide Cacchiarelli.; Denise Di Concilio; Francesco Panariello; Lucio Di Filippo; Marcello Salvi; Maria Concetta Cuomo; Patrizia Annunziata; Pellegrino Cerino; Valentina Bouche
EPI_ISL_1254732, EPI_ISL_1254736, EPI_ISL_1254793, EPI_ISL_1254814, EPI_ISL_1254822, EPI_ISL_1254842, EPI_ISL_1254891, EPI_ISL_1254901, EPI_ISL_1254913, EPI_ISL_1255003			
see above	Istituto Zooprofilattico Sperimentale del Mezzogiorno	Telethon Institute of Genetics and Medicine (TIGEM)	Antonio Grimaldi Patrizia Annunziata Francesco Panariello Biancamaria Pierri Claudia Tiberio Valentina Bouche Chiara Colantuono Maria Concetta Cuomo Denise Di Concilio Lucio Di Filippo Anna Manfredi Marcello Salvi Antonio Limone Luigi Atripaldi Pellegrino Cerino Andrea Ballabio Davide Cacchiarelli
EPI_ISL_1086065	Istituto Zooprofilattico Sperimentale del Mezzogiorno (IZSM)	Telethon Institute of Genetics and Medicine (TIGEM)	Andrea Ballabio; Anna Manfredi; Antonio Grimaldi; Antonio Limone; Biancamaria Pierri; Chiara Colantuono; Davide Cacchiarelli.; Denise Di Concilio; Francesco Panariello; Lucio Di Filippo; Marcello Salvi; Maria Concetta Cuomo; Patrizia Annunziata; Pellegrino Cerino; Valentina Bouche
EPI_ISL_763328	Istituto Zooprofilattico Sperimentale dell' Umbria e delle Marche -Togo Rosati	Istituto Superiore di Sanità	Gabriele Vaccari; Giovanni Ianiro; Ilaria Di Bartolo; Luca De Sabato; Massimo Biagetti; Monica Giammarioli
EPI_ISL_1020167	Istituto Zooprofilattico Sperimentale dell' Umbria e delle Marche -Togo Rosati Università degli Studi di Perugia	Department of Food safety, Nutrition and Veterinary public health - Istituto Superiore di Sanità	Antonella Mencacci; Barbara Camilloni; Gabriele Vaccari; Giovanni Ianiro; Ilaria Di Bartolo; Luca De Sabato; Massimo Biagetti; Monica Giammarioli
EPI_ISL_649940, EPI_ISL_722890, EPI_ISL_722893, EPI_ISL_722911, EPI_ISL_794801, EPI_ISL_1008674	Istituto Zooprofilattico Sperimentale della Puglia e della Basilicata	Istituto Zooprofilattico Sperimentale della Puglia e della Basilicata	Bianco A.; Capozzi L.; Chironna M.; Cipolletta D.; Del Sambro L.; Galante D.; Giannico A.; Loconsole D.; Manzulli V; Pace L.; Parisi A.; Rondinone V.; Sallustio A.; Simone D.
EPI_ISL_450793	Jamaica Ministry of Health and Wellness	Pathogen Discovery, Respiratory Viruses Branch, Division of Viral Diseases, Centers for Disease Control and Prevention	Anna Montmayeur; Anna Uehara; Bettina Bankamp; Clinton R. Paden; Haibin Wang; Jing Zhang; Krista Queen; Rachel Marine; Suxiang Tong; Yan Li; Ying Tao; Zachary Weiner
EPI_ISL_1233953	Jamil-ur-Rahman Center for Genome Research, Dr. Panjwani Center for Molecular Medicine and Drug Research	Jamil-ur-Rahman Center for Genome Research, Dr. Panjwani Center for Molecular Medicine and Drug Research	Ansari; I.A.; Irfan, M.; Khan; Nisa, Z.; Rashid, M.; S.K.; Shakeel, M.; Zaibunnisa
EPI_ISL_457935	KEMRI-Centre for Virus Research	KEMRI-Wellcome Trust Research Programme/KEMRI-CGMR-C Kilifi	Githinji G. et al 2020
EPI_ISL_568735, EPI_ISL_568805, EPI_ISL_806611, EPI_ISL_806632, EPI_ISL_806657, EPI_ISL_806660, EPI_ISL_806693, EPI_ISL_806704, EPI_ISL_855530, EPI_ISL_968871, EPI_ISL_968925			
see above	KEMRI-Wellcome Trust Research Programme/KEMRI-CGMR-C Kilifi	KEMRI-Wellcome Trust Research Programme/KEMRI-CGMR-C Kilifi	Githinji et al; Githinji et al 2020
EPI_ISL_407976	KU Leuven, Clinical and Epidemiological Virology	KU Leuven, Clinical and Epidemiological Virology	Annabel Rector; Bert Vanmechelen; Elke Wollants; Els Keyaerts; Lies Laenen; Marc Van Ranst; and Piet Maes
EPI_ISL_458209, EPI_ISL_476983, EPI_ISL_1093369, EPI_ISL_1382865	KU Leuven, Rega Institute, Clinical and Epidemiological Virology	KU Leuven, Rega Institute, Clinical and Epidemiological Virology	Bert Vanmechelen; Joan Marti-Carerras; Piet Maes; Tony Wawina-Bokalanga
EPI_ISL_475544	Karolinska Universitetslaboratoriet	The Public Health Agency of Sweden	Anna Risberg; Anna-Malin Linde; Karin Tegmark-Wisell; Maria Lind Karlberg; Mattias Haukland; Olov Svartstrom; Oskar Karlsson Lindsjo; Reza Advani; Sandra Broddesson; Shaman Muradrasoli
EPI_ISL_1313460	Karolinska University Hospital	Karolinska University Hospital	Annelie Bjerkner; Isak Sylvin; Jan Albert; Karolina Ininbergs; Lina Guerra Blomqvist; Lynda Eneh; Martin Ekman; Martina Wahlund; Robert Dyrdak; Sandra

			Broddesson; Tanja Normark; Tobias Allander; Valtteri Wirta; Zhibing Yun
EPI_ISL_934333, EPI_ISL_1168039	Klinisk mikrobiologi	The Public Health Agency of Sweden	Anna Risberg; Anna-Malin Linde; Carlo Berg; Karin Tegmark-Wisell; Maria Lind Karlberg; Mattias Haukland; Mia Brytting; Noura Walai; Oskar Karlsson Lindsjo; Petra Edquist; Petra Holmstrom; Reza Advani; Sofia Stamouli
EPI_ISL_718004	Lab voor klinische biologie	Onderzoeksgroep Virologie	Bruno Verhasselt; Hans Nauwynck; Laurens Lambrechts; Linos Vandekerckhove; Marthe Pauwels; Nick Vereecke; Sebastiaan Theuns
EPI_ISL_1307953	Lab. Microbiologia e Virologia Cotugno A.O. dei Colli	TIGEM	Antonio Grimaldi Patrizia Annunziata Francesco Panariello Biancamaria Pierri Claudia Tiberio Valentina Bouche Chiara Colantuono Maria Concetta Cuomo Denise Di Concilio Lucio Di Filippo Anna Manfredi Marcello Salvi Antonio Limone Luigi Atripaldi Pellegrino Cerino Andrea Ballabio Davide Cacchiarelli
EPI_ISL_855613, EPI_ISL_856694, EPI_ISL_856700, EPI_ISL_860095	Lab. Microbiologia e Virologia, Cotugno, A.O. dei Colli	Lab. Microbiologia e Virologia, Cotugno, A.O. dei Colli	Anna Perfetti; Claudia Tiberio; Luigi Atripaldi
EPI_ISL_775219	Laboratoire Biolife	Laboratoire de Biotechnologie	Afaf Alaoui; Amina Benouda; Bouchra Belfquih; Hanae Dakka; Lahcen belyamani; Mohamed Chenaoui; Mohammed Walid Chemao Elfihri; Mouna Ouadghiri; Otmane Touzani; Saaid Amzazi and Azeddine Ibrahimi; Tarik Aanniz
EPI_ISL_445075	Laboratoire National de Sante, Microbiology, Virology	Laboratoire National de Sante, Microbiology, Epidemiology and Microbial Genomics	Anke Wienecke-Baldacchino; Ardashel Latsuzbaia; Catherine Ragimbeau; Guillaume Fournier; Jessica Tapp; Joel Mossong; Tamir Abdelrahman; Trung Nguyen Nguyen
EPI_ISL_413593	Laboratoire National de Santé	Erasmus Medical Center	Anne van der Linden; Annemiek van der Eijk; Bas Oude Munnink; Claudia Schapendonk; Corine GeurtsvanKessel; David Nieuwenhuijse; G. Fournier; Irina Chestakova; J. Mossong; Jeroen van Kampen; Jolanda Voermans; Marion Koopmans; Mark Pronk; Pascal Lexmond; Reina Sikkema; Richard Molenkamp; T. Abdelrahman; T. Nguyen; on behalf of the Dutch national COVID-19 response team.
EPI_ISL_1116464, EPI_ISL_1116468, EPI_ISL_1118884	Laboratoire de Microbiologie- CHU Habib Bourguiba - Sfax	Laboratoire des Procédés de Criblage Moléculaire et Cellulaire-Centre de Biotechnologie de Sfax	A. and Masmoudi, S.; Abdelmoulah, F.; Abid, N.; Ajili, F.; Aouni, M.; Ben Ayed, I.; Bensaid, M.; Chtourou, A.; Elargoubi, A.; Fki-berrajah, L.; Gaaloul, I.; Gargouri, S.; Hammami, A.; Kamoun, S.; Karray Hakim, H.; Kharat, N.; Mastouri, M.; Mhalla, S.; Nabli, A.; Rebai; Smeti, I.; Souissi, A.; Stambouli, N.; Turki, M.
EPI_ISL_476026	Laboratoire de Recherche et d'Analyses Médicales de la Gendarmerie Royale	Laboratoire de Recherche et d'Analyses Médicales de la Gendarmerie Royale	Amal SOUIRI; Sanaâ Lemriss; Saâd EL KABBAJ
EPI_ISL_954722	Laboratoire de santé publique du Québec	Laboratoire de santé publique du Québec	Guillaume Bourque; Ioannis Ragoussis; Jesse Shapiro; Mark Lathrop and Michel Roger on behalf of the CoVSeQ research group (http://covseq.ca/researchgroup); Sandrine Moreira
EPI_ISL_1197037	Laboratoire de virologie clinique - Institut Pasteur de Tunis	1-Laboratory of Microbiology, National Reference Lab, Charles Nicolle Hospital; 2-University of Tunis ElManar, Faculty of Medicine of Tunis, LR99ES09, Tunis, Tunisia	Alia BenKahla; Anissa Chouikha; Fares Wasfi; Henda Triki; Ilhem Boutiba-Ben Boubaker.; Imen Kacem; Ines Mdini; Jalila Ben Khelil; Maher Kharrat; Manel Ben Sassi; Mariem Gdoura; Mouna Ben Sassi; Mouna Safer; Nissaf Ben Alaya; Riadh Daghfous; Riadh Gouider; Roua Ben Othman; Salma Abid; Sameh Trabelsi; Sana Ferjani; Sara Chammam; Sondes Haddad
EPI_ISL_476830	Laboratoire des Fièvres Hémorragiques Virales du Benin	Charité-Universitätsmedizin Berlin	Anges; Drexler; Jan Felix; Moreira-Soto Andres; Sander Anna-Lena; Yadouleton
EPI_ISL_910424, EPI_ISL_910734, EPI_ISL_1383433	Laboratoire national de sante, Microbiology, Virology	Laboratoire national de sante, Microbiology, Microbial Genomics Platform	Anke Wienecke-Baldacchino; Catherine Ragimbeau; Fatu Djabi; Jessica Tapp; Lise Pignon; Raoul Salmon; Tamir Abdelrahman; Trung Nguyen Nguyen
EPI_ISL_740005, EPI_ISL_740161, EPI_ISL_744631	Laboratoire national de santé, Microbiology, Virology	Laboratoire national de santé, Microbiology, Microbial Genomics Platform	Anke Wienecke-Baldacchino; Catherine Ragimbeau; Fatu Djabi; Jessica Tapp; Lise Pignon; Raoul Salmon; Tamir Abdelrahman
EPI_ISL_1384629	Laboratoires Reunis	Laboratoire national de sante, Microbiology, Microbial Genomics Platform	Anke Wienecke-Baldacchino; Bernard Weber; Catherine Ragimbeau; Fatu Djabi; Jessica Tapp; Lise Pignon; Raoul Salmon; Tamir Abdelrahman
EPI_ISL_1384477, EPI_ISL_1384536, EPI_ISL_1384542	Laboratoires d'analyses medicales - Ketterthill	Laboratoire national de sante, Microbiology, Microbial Genomics Platform	Anke Wienecke-Baldacchino; Caroline Scheiber; Catherine Ragimbeau; Fatu Djabi; Jessica Tapp; Lise Pignon; Raoul Salmon; Serge Vedy; Tamir Abdelrahman
EPI_ISL_1299547	Laboratorio Analisi Osp. Città di Castello - Azienda USL Umbria1	Istituto Zooprofilattico Sperimentale dell'Abruzzo e Molise "G. Caporale"	Ancora M; Calistri P; Cammà C; Curini V; Di Domenico M; Di Pasquale A; Lorusso A; Malagigi V; Mangone I; Marcacci M; Puglia I; Rinaldi A; Savini G; Scialabba S; Tacconi P
EPI_ISL_458084	Laboratorio Biologia Molecolare Sars Cov2 - UOC Laboratorio Analisi - Servizio Medicina di Laboratorio, Ospedale "San Francesco" - ATS-ASSL Nuoro	Laboratorio specialistico UOC Ematologia - Ospedale "San Francesco" - ATS-ASSL Nuoro	Asproni Rosanna; Casu Gavino; Fancello Tatiana; Fiamma Maura; Floris Anna Rita; Lo Maglio Iana; Mameli Giuseppe.; Monne Maria Itria; Palmas Angelo Domenico; Piras Giovanna; Sanna Filomena; Sulis Vincenzo; Toja Alessandro
EPI_ISL_637107, EPI_ISL_1191739	Laboratorio Biologia Molecolare Sars Cov2 - UOC Laboratorio Analisi - Servizio Medicina di Laboratorio, Ospedale "San Francesco" - ATS-ASSL Nuoro	Laboratorio specialistico UOC Ematologia - Ospedale "San Francesco" - ATS-ASSL Nuoro	; Asproni Rosanna; Casu Gavino; Fancello Tatiana; Fiamma Maura; Floris Anna Rita; Lo Maglio Iana; Mameli Giuseppe; Monne Maria Itria; Palmas Angelo Domenico; Piras Giovanna; Sanna Filomena; Sulis Vincenzo; Toja Alessandro
EPI_ISL_613560	Laboratorio Biologia Molecolare Sars Cov2 - UOC Laboratorio Analisi - Servizio Medicina di Laboratorio, Ospedale "San Francesco" - ATS-ASSL Nuoro Via Manniròni 1, 08100 Nuoro	Laboratorio specialistico UOC Ematologia - Ospedale "San Francesco" - ATS-ASSL Nuoro Nuoro	Asproni Rosanna; Casu Gavino; Fancello Tatiana; Fiamma Maura; Floris Anna Rita; Lo Maglio Iana; Mameli Giuseppe; Monne Maria Itria; Palmas Angelo Domenico; Piras Giovanna; Sanna Filomena; Sulis Vincenzo; Toja Alessandro
EPI_ISL_547965	Laboratorio Biologia Molecolare SarsCov2 UOC LAboratorio Analisi SErvizio Medicina di Laboratorio Ospedale San Francesco ATS-ASSL Nuoro	Laboratorio Specialistico UOC Ematologia Ospedale San Francesco - ATS ASSL NUORO	Asproni Rosanna; Casu Gavino; Fancello Tatiana; Fiamma Maura; Floris Anna Rita; Lo Maglio Iana; Mameli Giuseppe.; Monne Maria Itria; Palmas Angelo Domenico; Piras Giovanna; Sanna Filomena; Sulis Vincenzo; Toja Alessandro
EPI_ISL_1395930	Laboratorio Central de la Ciudad de Santa Fe	Grupo de Genómica y Bioinformática del Instituto de Investigación de la Cadena Láctea CONICET-INTA on behalf of 'Proyecto Argentino Interinstitucional de genomica de SARS-CoV-2' (PAIS Consortium)	AF; Amadio; C; Eberhardt; G; Irazoqui; JM; MF; Mugna; Ojeda; Pastor; Rompato; V
EPI_ISL_837551, EPI_ISL_837596	Laboratorio Nacional de Salud	Laboratory of Respiratory Viruses and Measles, Oswaldo Cruz Institute, FIOCRUZ	Ana Carolina Mendonca; Anna Carolina Paixao; Cesar Roberto Conde Pereira; Claudia Estrada; Fernando Motta; Luciana Appolinario; Marilda Siqueira on behalf of the Fiocruz COVID-19 Genomic Surveillance Network; Paola Resende
EPI_ISL_1278283, EPI_ISL_1278284	Laboratorio de Biologia Molecular, SEDES-Potosi	Molecular Genetics Laboratory, Instituto de Investigaciones Químicas, Universidad Mayor de San Andrés	Aneth Vasquez Michel; Oscar M. Rollano-Peñaloza
EPI_ISL_1068130	Laboratorio de Ecologia de Doencas Transmissiveis na Amazonia, Instituto Leonidas e Maria Deane - Fiocruz Amazonia	Laboratorio de Ecologia de Doencas Transmissiveis na Amazonia, Instituto Leonidas e Maria Deane - Fiocruz Amazonia	André Corado; Debora Duarte; Felipe Naveca on behalf of the Fiocruz COVID-19 Genomic Surveillance Network; Fernanda Nascimento; George Silva; Karina Pessoa; Luciana Gonçalves; Maria Júlia Brandão; Matilde Mejía; Michele Jesus; Valdinete Nascimento; Victor Souza; Ágatha Costa
EPI_ISL_779170	Laboratorio de Infectología, Servicio de Infectología, Hospital Universitario Dr. José Eleuterio González - Universidad Autónoma de Nuevo León	Laboratorio de Infectología Molecular, Departamento de Bioquímica y Medicina Molecular,Facultad de Medicina - Universidad Autónoma de Nuevo León	Adrian Camacho-Ortiz; Ana M. Rivas-Estilla; Daniel Arellanos-Soto; Eduardo Perez-Alba; Elvira Garza-González; Kame A. Galán-Huerta; Laura Nuzzolo-Shihadeh; María F. Herrera-Saldivar; Natalia Martínez-Acuña; Paola Bocanegra-Ibarias; Samantha M. Flores-Treviño; Sonia A. Lozano-Sepúlveda
EPI_ISL_514339, EPI_ISL_1138413	Laboratorio de Referencia Nacional de Virus Respiratorio. Instituto Nacional de Salud Perú	Laboratorio de Referencia Nacional de Biotecnología y Biología Molecular. Instituto Nacional de Salud Perú	Carlos Padilla Rojas; Henri Bailon Calderon; Johanna Balbuena Torres; Johanna Balbuena Torrez; Karolyn Vega Chozo; Luis Barcena; Marco Galarza Perez; Maribel Huaringa Nuñez; Nancy Rojas Serrano; Nancy Rojas Serrano.; Omar Caceres Rey; Priscila Lope Pari
EPI_ISL_1111229	Laboratorio de Referencia Nacional de Virus Respiratorio. Instituto Nacional de Salud Perú	Laboratorio de Referencia Nacional de Enteropatógenos. Instituto Nacional de Salud del Perú	Fiorella Orellana Peralta; Iris Silva Molina; Junior Caro Castro; Ronnie Gavilan Chavez; Veronica Hurtado Vela; Willi Quino Sifuentes
EPI_ISL_568514	Laboratorio de Referencia Nacional de Virus Respiratorios, Instituto Nacional de Salud Peru	Laboratorio de Genómica Microbiana, Universidad Peruana Cayetano Heredia	Alejandra Dávila-Barclay; Brenda Ayzanoa; Camila Castillo-Vilcahuaman; Guillermo Salvatierra; Janet Huancachoque; Luis González; Maribel Huaringa; Pablo Tsukayama; Pedro E. Romero; Pool Marcos
EPI_ISL_717791	Laboratorio de Virologia Molecular / UFRJ	Bioinformatics Laboratory / LNCC	Alexandra L Gerber; Amilcar Tanuri; Ana Paula de C Guimarães; Ana Tereza R de Vasconcelos; Andréa Cony Cavalcanti; Carolina M Voloch; Claudia dos Santos Rodrigues; Cynthia C Cardoso; Diana Mariani; Luiz G P de Almeida; Otavio Bustrolini; Ronaldo da Silva F Jr; Terezinha M P P Castiñeira
EPI_ISL_1396122	Laboratorio de Virus Respiratorios y Neurovirosis. Hospital Señor del Milagro	Instituto de Patologia Vegetal (CIAP-INTA) on behalf of 'Proyecto Argentino Interinstitucional de genomica de	A. Dra. Raskovsky Viviana; Amadio; Debat, HJ.; Dr. Lavaque Esteban; Dra. Veronica Lesser. Tecnica: Pamela Cajal; FD; Fernanda Aguero.; Fernández; Irazoqui, M.; Marquez, N.

		SARS-CoV-2' (PAIS Consortium)	
EPI_ISL_1396358	Laboratorio del Hospital Regional Ushuaia Gdor. Ernesto Campos	Nodo de Secuenciación Tierra del Fuego - Hospital Regional Ushuaia - Centro Austral De Investigaciones Científicas - Universidad Nacional De Tierra Del Fuego on behalf of 'Proyecto Argentino Interinstitucional de genomica de SARS-CoV-2' (PAIS Consortium)	Alejandro Ezequiel Rojas; Carina Andrea De Roccis; Carolina Beatriz Yulan; Cristina Fernanda Nardi; Fernando Gallego; Gabriel Alejandro Castro; Ivan Dario Gramundi; Manuel Fabian Boutureira; Santiago Guillermo Ceballos; Silvana Beatriz Cáceres
EPI_ISL_541650, EPI_ISL_644565, EPI_ISL_904008	Laboratory Diagnostic, Veterinary Specialized Institute Kraljevo	Laboratory Diagnostic, Veterinary Specialized Institute Kraljevo	Afonso, C.; Banovic Djeri, B.; Jankovic, M.; Jovanovic, T.; Knezevic, A.; Petrovic, T.; Sekler, M.; Tesovic, B.; Vidanovic, D.; Volkening, J.
EPI_ISL_933533, EPI_ISL_1209407	Laboratory for HIV and opportunistic infections diagnosis The Republican Research and Practical Center for Epidemiology and Microbiology (RRPCEM)	Laboratory for HIV and opportunistic infections diagnosis The Republican Research and Practical Center for Epidemiology and Microbiology (RRPCEM)	Anatoly Krasko; Artur Akhremchuk; Elena Gasich; Kirill Bulda; Leonid Valentovich; Leonid Valentovich; Vladimir Gorbunov
EPI_ISL_779713	Laboratory of Infectious Diseases, Department of Biomedical and Clinical Sciences L. Sacco, University of Milan	Laboratory of Infectious Diseases, Department of Biomedical and Clinical Sciences L. Sacco, University of Milan	Alessia Lai; Annalisa Bergna; Carla Della Ventura; Claudia Balotta; Gianguglielmo Zehender on behalf of SARS-CoV-2 ITALIAN RESEARCH ENTERPRISE-(SCIRE) Collaborative Group; Massimo Galli
EPI_ISL_717979	Laboratory of Microbiology and Infectious Diseases, Faculty of Veterinary Medicine, Aristotle University of Thessaloniki, University Campus, 541 24, Thessaloniki, Greece.	Laboratory of Biology, Department of Medicine, Democritus University of Thrace, Alexandroupolis, Greece	Bampali M.; Chaintoutis S.; Dimitriou M.; Dovas C.; Dovrolis N.; Karakasiliotis I.
EPI_ISL_437899	Laboratory of Microbiology, Medical School, National and Kapodistrian University of Athens	Laboratory of Biology, Department of Medicine, Democritus University of Thrace	Bampali, M.; Dovrolis, N.; Froukala, E.; Gatzidou, E.; Kassela K.; N. and Karakasiliotis, I.; Spanakis; Stavropoulou, A.; Tsakris, A.; Veletza, S.
EPI_ISL_479791	Laboratory of Molecular Virology of the International Centre for Genetic Engineering and Biotechnology (ICGEB)	ARGO Open Lab Platform for Genome Sequencing	Confalonieri M; Confalonieri P; D; D'Agaro P; Dal Monego S; Licastro; Marcello A; Rajasekharan S; Salton F; Segat L
EPI_ISL_801761	Laboratory of Molecular Virology, Pontificia Universidad Católica de Chile	MSHS Pathogen Surveillance Program	Adolfo Garcia-Sastre; Adriana van De Guchte; Ajay Obla; Ana Maria Contreras; Ana S. Gonzalez-Reiche; Bremy Alburquerque; Carlos Palma; Constanza Maldonado; Edward C. Holmes; Eileen Serrano; Erick Salinas; Hala Alshammary; Harm van Bakel; Jayeeta Dutta; Jorge Levican; Juan Soto; Leonardo I. Almonacid; M. Belen Leyton; Marcela Ferres; Matthew M. Hernandez; Melissa Smith; Rafael A. Medina.; Robert Sebra; Shwetha Hara Sridhar; Tamara García-Salum; Viviana Simon; Ying-Chih Wang; Zenab Khan
EPI_ISL_1402430	Laboratory of Respiratory Viruses and Measles, Oswaldo Cruz Institute, FIOCRUZ	Laboratory of Respiratory Viruses and Measles, Oswaldo Cruz Institute, FIOCRUZ	Alex Pauvolid-Correa; Alice Sampaio Rocha; Ana Beatriz Machado Lima; Ana Carolina Mendonca; Anna Carolina Paixao; Fernando Motta; Luciana Appolinario; Marilda Siqueira on behalf of the Fiocruz COVID-19 Genomic Surveillance Network; Mia Ferreira Araujo; Paola Resende; Renata Serrano Lopes
EPI_ISL_451302	Laboratory of Virology, INMI Lazzaro Spallanzani IRCCS	Laboratory of Virology, INMI Lazzaro Spallanzani IRCCS	Antonino Di Caro; Barbara Bartolini; Cesare E.M. Gruber; Francesco Messina; Giuseppe Ippolito; Maria R. Capobianchi; Martina Rueca
EPI_ISL_454575	Laboratory of virology, National Center of Expertise	Laboratory of molecular-genetic research, National Center for Expertise, Kazakhstan National Center for Biotechnology, Kazakhstan	; Abdaliyev Askar; Akhmetollayev Ilyas; Amirgazin Asylulan; Aushakhmetova Zabira; Kalendar Ruslan; Lutsay Viktoriya; Rakhmetova Akbota; Ramankulov Yerlan; Shevtsov Alexandr
EPI_ISL_454584, EPI_ISL_454585	Laboratory of virology, National Center of Expertise	Laboratory of molecular-genetic research, National Center for Expertise, Kazakhstan National Center for Biotechnology, Kazakhstan	; Abdaliyev Askar; Akhmetollayev Ilyas; Amirgazin Asylulan; Aushakhmetova Zabira; Kalendar Ruslan; Lutsay Viktoriya; Rakhmetova Akbota; Ramankulov Yerlan; Shevtsov Alexandr
EPI_ISL_1334574, EPI_ISL_1364879	Laboratory of virology, National center of expertise	RSE "National Center for Biotechnology" and RSE "National Center of Expertise"	Abdaliyev Askar; Amirgazin Asylulan; Balykbaev Kanat; Kamalova Dinara; Ramankulov Yerlan; Sharipova Saule; Shevtsov Alexandr; Tungushbayev Talgat
EPI_ISL_576119	Laboratory, The Bio Arte Limited	Laboratory, The Bio Arte Limited	Biazzo, M.; Madeddu, S.; Pinzauti, D.; Santoro, F.
EPI_ISL_576121	Laboratory, The Bio Arte Limited	Laboratory, The Bio Arte Limited	Biazzo, M.; Madeddu, S.; Pinzauti, D.; Santoro, F.
EPI_ISL_1213249	Laboratório HLA/UERJ	Bioinformatics Laboratory / LNCC	Alessandra P Lamarca; Alexandra L Gerber; Ana Paula Melo Mariano; Ana Paula de C Guimarães; Ana Tereza R Vasconcelos; Angela Maria Guimarães Santos; Bianca Mendes Maciel; Danielle Angst Secco; Eduardo Sérgio Soares Sousa; Eloiza Helena Campana; Francisco Paulo Freire Neto; George Rego Albuquerque; Kátia Castanho Scortecci; Lucymara Fassarella Agnez Lima; Luiz G P de Almeida; Luís Cristóvão Porto; Otavio J. Brustolini; Paulo Ricardo Nascimento; Ronaldo da Silva Francisco Jr; Sandra Rocha Gadelha; Selma Maria Bezerra Jeronimo; Vinícius Pietta Perez
EPI_ISL_1233663	Labormedizinisches Zentrum Dr Risch	University Hospital Basel, Clinical Bacteriology	Adrian Egli; Alfredo Mari; Hans Hirsch; Helena MB Seth-Smith; Julia Bielicki; Karoline Leuzinger; Lorenz Risch; Madlen Stange; Manuel Battegay; Martin Risch; Nadia Wohlwend; Tim Roloff
EPI_ISL_738317, EPI_ISL_812279	Landstuhl Regional Medical Center	United States Air Force School of Aerospace Medicine	Amanda Javorina; Anthony Fries; Clarise Starr; Cole Anderson; Elizabeth Macias; Fritz Castillo; Jennifer Meyer; Sarah Purves; William Gruner
EPI_ISL_629009	Laverty Pathology	NSW Health Pathology - Institute of Clinical Pathology and Medical Research; Westmead Hospital; University of Sydney	CIDM-PH et al.
EPI_ISL_708939, EPI_ISL_730882, EPI_ISL_964220, EPI_ISL_1189707	Lighthouse Lab in Alderley Park	Wellcome Sanger Institute for the COVID-19 Genomics UK (COG-UK) Consortium	Cordelia Langford; David K. Jackson; Dominic Kwiatkowski; Ewan Harrison; Ian Johnston; Jacquelyn Wynn; Jeffrey Barrett; John Sillitoe on behalf of the Wellcome Sanger Institute COVID-19 Surveillance Team; Mairead Hyland; Roberto Amato; Sonia Goncalves; The Lighthouse Lab in Alderley Park and Alex Alderton
EPI_ISL_580822	Lighthouse Lab in Alderley Park	Wellcome Sanger Institute for the COVID-19 Genomics UK (COG-UK) consortium	Cordelia Langford; David K. Jackson; Dominic Kwiatkowski; Ewan Harrison; Ian Johnston; Jacquelyn Wynn; John Sillitoe on behalf of the Wellcome Sanger Institute COVID-19 Surveillance Team; Mairead Hyland; Roberto Amato; Sonia Goncalves; The Lighthouse Lab in Alderley Park and Alex Alderton
EPI_ISL_556699	Lighthouse Lab in Cambridge	Wellcome Sanger Institute for the COVID-19 Genomics UK (COG-UK) consortium	Cordelia Langford; David K. Jackson; Dominic Kwiatkowski; Ewan Harrison; Ian Johnston; John Sillitoe on behalf of the Wellcome Sanger Institute COVID-19 Surveillance Team; Rob Howes; Roberto Amato; Sonia Goncalves; The Lighthouse Lab in Cambridge and Alex Alderton
EPI_ISL_1344147	Lighthouse Lab in Milton Keynes	Wellcome Sanger Institute for the COVID-19 Genomics UK (COG-UK) Consortium	Cordelia Langford; David K. Jackson; Dominic Kwiatkowski; Ewan Harrison; Ian Johnston; Jeffrey Barrett; John Sillitoe on behalf of the Wellcome Sanger Institute COVID-19 Surveillance Team; Roberto Amato; Sonia Goncalves; The Lighthouse Lab in Milton Keynes and Alex Alderton
EPI_ISL_552233	Lighthouse Lab in Milton Keynes	Wellcome Sanger Institute for the COVID-19 Genomics UK (COG-UK) consortium	Cordelia Langford; David K. Jackson; Dominic Kwiatkowski; Ewan Harrison; Ian Johnston; John Sillitoe on behalf of the Wellcome Sanger Institute COVID-19 Surveillance Team (http://www.sanger.ac.uk/covid-team); Roberto Amato; Sonia Goncalves; The Lighthouse Lab in Milton Keynes and Alex Alderton
EPI_ISL_636906	Lithuanian University of Health Sciences Hospital, Department of Laboratory Medicine	Lithuanian University of Health Sciences, Molecular cardiology lab.	Arnoldas Pautienius; Astra Vitkauskiene; Dovydas Gecys; Ingrida Olendrait; Kamile Tamusauskaite; Laura Pareckaite; Lukas Zemaitis; Vaiva Lesauskaite
EPI_ISL_804581, EPI_ISL_896180	MEPHI, Aix Marseille University	MEPHI, Aix Marseille University	Anthony LEVASSEUR
EPI_ISL_903377	MOH - Jaber Al-Ahmad Hospital (Innovation Research Laboratory)	MOH - Jaber Al-Ahmad Hospital (Innovation Research Laboratory)	Mohammad Alghounaim; Salman Al-Sabah
EPI_ISL_471158, EPI_ISL_471165, EPI_ISL_471166, EPI_ISL_561024, EPI_ISL_561213, EPI_ISL_811015, EPI_ISL_1216127, EPI_ISL_1216133, EPI_ISL_1234529, EPI_ISL_1234533			
see above	MRCG at LSHTM Genomics lab	MRCG at LSHTM Genomics lab	Abdoulie Kanteh; Abdul Karim sesay; Bakary Sanyang; Jarra Manneh; Mariama Kujabi; Sesay et al
EPI_ISL_1181240	MVZ Dr. Eberhard & Partner Dortmund	Bielefeld University	Alexander Sczyrba; David Brandt; Jörn Kalinowski; Levin-Joe Klages; Marina Simunovic; Markus Haak; Svenja Vinke; Tobias Busche
EPI_ISL_860463	MVZ Labor Krone GbR	Center of Medical Microbiology, Virology, and Hospital Hygiene, University of Duesseldorf	Alexander Dilthey; Andreas Walker; André Heimbach; Bärbel Lippke; Carsten Tiemann; Dennis Deschka; Janine Silvery; Julia Fazaal; Jörg Timm; Kerstin Ludwig; Klaus Pfeffer; Malte Kohns Vasconcelos; Per Hoffmann; Tobias Wienemann; Torsten Houwaart
EPI_ISL_873165	Medical Laboratory Sciences, Arab American University	Medical Laboratory Sciences, Arab American University	Al-Jawabreh, A.; Al-Jawabreh, H.; Dumaidi, k.; Ereqat, S.; Nasereddin, A.
EPI_ISL_1367825	Michigan Department of Health and Human Services, Bureau	Michigan Department of Health and Human Services, Bureau	Blankenship HM; Riner D; Soehnlen MK

	of Laboratories	of Laboratories	
EPI_ISL_480342	Microbial Genomics Laboratory, Institut Pasteur de Montevideo	Microbial Genomics Laboratory, Institut Pasteur de Montevideo	Cecilia Salazar; Gonzalo Moratorio; Gregorio Iraola; Ignacio Ferrés; Marianoel Pereira; Pilar Moreno
EPI_ISL_977174, EPI_ISL_1260950	Microbiologia e Virologia	Istituto Zooprofilattico Sperimentale delle Venezie	Adelaide Milani; Alessia Schivo; Alice Fusaro; Ambra Pastori; Annalisa Salviato; Antonia Ricci; Bianca Zecchin; Calogero Terregino; Edoardo Giussani; Erika Giorgia Quaranta; Isabella Monne; Luca Tassoni
EPI_ISL_591596	Microbiological Diagnostic Unit - Public Health Laboratory (MDU-PHL)	MDU-PHL	M. B.; Sait, M.; Schultz; Seemann T.; Sherry, N.
EPI_ISL_1208753	Microbiology Department, Laboratori Clínic Metropolitana Nord. Hospital Universitari Germans Trias i Pujol.	Can Ruti SARS-CoV-2 Sequencing Hub (HUGTiP/IrsiCaixa/IGTP)	Alba Sánchez; Anna Not; Antoni E Bordoy; Bonaventura Clotet; Carol Galvez Maria Casadellà; Cristina Casañ; Cristina Esteban; Francesc Catala-Moll; Gemma Clara; Ignacio Blanco; Irina Pey; Jordi Barretina; Julia G Prado; Marc Noguera-Julian; Mariona Parera; Mercedes Guerrero; Montserrat Giménez; Pere-Joan Cardona; Pilar Armengol; Roger Paredes; Verónica Saludes; and Elisa Martró on behalf of the Can Ruti SARS-CoV-2 Sequencing Hub.
EPI_ISL_1064028, EPI_ISL_1064051, EPI_ISL_1064056, EPI_ISL_1064082, EPI_ISL_1064089, EPI_ISL_1064091, EPI_ISL_1064092, EPI_ISL_1064102, EPI_ISL_1064104			
see above	Microbiology and Virology Unit, Azienda Ospedale Padova, Padova, Italy	Department of Molecular Medicine, Computational Medicine Group, Univeresity of Padova, Padova, Italy	Andrea Crisanti; Andrea Spitaleri; Claudia Del Vecchio; Daniela Maria Cirillo; Dejan Lazarevic; Elisa Franchin; Enrico Lavezzo; Fabio Simeoni; Federico Bianca; Francesca Saluzzo; Francesco Onelia; Giovanni Lorenzin; Giovanni Tonon; Laura Manuto; Marco Grazioli; Stefano Toppo
EPI_ISL_911526	Microbiology and Virology Unit, Florence Careggi University Hospital	Microbiology and Virology Unit, Florence Careggi University Hospital	Alberto Antonelli; Emanuele Gori; Fabio Morecchiato; Gian Maria Rossolini; Ilaria Baccani; Marco Coppi; Noemi Aiezza; Vincenzo Di Pilato
EPI_ISL_1181737, EPI_ISL_1181740, EPI_ISL_1181761, EPI_ISL_1181783, EPI_ISL_1181788, EPI_ISL_1181793, EPI_ISL_1181814			
see above	Microbiology and Virology Unit,Azienda Ospedale Padova,Padova,Italy	Department of Molecular Medicine,Computational Medicine Group,Univeresity of Padova,Padova,Italy	Andrea Crisanti; Claudia Del Vecchio; Elisa Franchin; Enrico Lavezzo; Federico Bianca; Francesco Onelia; Laura Manuto; Marco Grazioli; Stefano Toppo
EPI_ISL_547433, EPI_ISL_547436, EPI_ISL_547437, EPI_ISL_615044	Microbiology, Department of Pathology, St. Bernard's Hospital, Gibraltar Health Authority	Respiratory Virus Unit, Microbiology Services Colindale, Public Health England	Charlotte Gillborn-Jones (Gibraltar); Dr Nicholas Cortes (Gibraltar); PHE Covid Sequencing Team
EPI_ISL_486652, EPI_ISL_486655, EPI_ISL_486657, EPI_ISL_486659, EPI_ISL_486664	Microbiology, Virology and Biemergency Laboratory-ASST FBF Sacco	Microbiology, Virology and Biemergency Laboratory-ASST FBF Sacco	Comandatore F; Mancon A; Micheli V; Rimoldi SG; Romeri F
EPI_ISL_894258, EPI_ISL_1379953	Ministry of Health Turkey	Ministry of Health Turkey	Aye Baak Alta; Fatma Bayrakdar; Gulay Korukluoglu; Gülay Korukluolu; Suleyman Yalcin; Süleyman Yalcin; Yasemin Cosgun; Yasemin Cogun
EPI_ISL_451307, EPI_ISL_460089, EPI_ISL_460090, EPI_ISL_460091	Molecular Virology Unit, Fondazione IRCCS Policlinico San Matteo , Pavia	Laboratory of Virology, INMI Lazzaro Spallanzani IRCCS	Antonino Di Caro; Antonio Piralla; Barbara Bartolini; Cesare E.M. Gruber; Fausto Baldanti; Maria R. Capobianchi; Martina Rueca
EPI_ISL_1133160, EPI_ISL_1133201, EPI_ISL_1133202	Molecular Virology Unit, Microbiology and Virology Dependment, Fondazione IRCCS Policlinico San Matteo, Pavia	Molecular Virology Unit, Microbiology and Virology Dependment, Fondazione IRCCS Policlinico San Matteo, Pavia	Antonio Piralla; Fausto Baldanti; Federica Giardina; Federica Novazzi; Gherard Batisti Biffignandi; Monica Tallarita; Stefania Paolucci; Stefano Gaiarsa
EPI_ISL_470539, EPI_ISL_653924	Molecular diagnostic laboratory of Federal Budget Institution of Science "Central Research Institute of Epidemiology" of The Federal Service on Customers' Rights Protection and Human Well-being Surveillance	Group of Genomics and Postgenomic Technologies of Central Research Institute of Epidemilology	Akimkin VG; Dudorova A.V.; Kaptelova VV; Korneenko EV; Samoilov AE; Shipulina OY; Sizova TV; Speranskaya AS; Tivanova EV
EPI_ISL_605897	NGS Lab, DNA SOLUTION LTD.	NGS Lab, DNA SOLUTION LTD.	Chowdhury, M.; H.U.; Haider; Hasan; K.N.; Khaleque, A.; Khan; Khan, M.; M.F.A.; M.H.; M.I.; M.N.I.; Polol; Rabbi; Rahman, M.; Razu; Sufian, A.
EPI_ISL_418242, EPI_ISL_766864	NIC Viral Respiratory Unit - Institut Pasteur of Algeria	National Reference Center for Viruses of Respiratory Infections, Institut Pasteur, Paris	Angela Brisebarre; Etienne Simon-Lorière; Fawzi Derrar; Flora Donati; Marion Barbet; Maud Vanpeene; Mélanie Albert; Méline Bizard; Sylvie Behillil; Sylvie van der Werf; Vincent Enouf
EPI_ISL_1278156	NL-Dr. Leonard A. Miller Centre for Health Services	National Microbiology Laboratory (NML)	Adel Malek; Anna Majer; Anneliese Landgraff; CanCOGeN's metadata curation team; Darian Hole; Elsie Grudeski; Gary Van Domselaar; George Zahariadis; Grace Seo; Jennifer Tanner; Kerri Smith; Kirsten Biggar; Laura Gilbert; Madison Chapel; Morag Graham; Natalie Knox; Nathalie Bastien; Philip Mabon; Public Health Agency of Canada CanCOGeN team; Rhiannon Huzarewich; Robert Needle; Russell Mandes; Shari Tyson; Timothy Booth; Yan Li; Yang Yu
EPI_ISL_1233623	National Center for Infectious and Parasitic Diseases (NCIPD)	National Center for Infectious and Parasitic Diseases (NCIPD)	Alexiev et al
EPI_ISL_626569, EPI_ISL_626627	National Centre for Communicable Disease (NCCD) National Influenza Center	National Centre for Communicable Disease (NCCD) National Influenza Center	Ankhbayar S; Bayasgalan N; Darmaa B; Erden-Ochir Ts; Naranzul Ts; Nyamkhuu D; Nymadawa P Chang-Seon Song
EPI_ISL_626566	National Centre for Communication Disease (NCCD) National Influenza Center	National Centre for Communication Disease (NCCD) National Influenza Center	Ankhbayar S; Bayasgalan N; Chang-Seon Song; Darmaa B; Erden-Ochir Ts; Naranzul Ts; Nymadawa P; Tsogtbaatar B
EPI_ISL_482492	National Centre for Disease control (NCDC)	NCDC/CSIR-IGIB	Anurag Agrawal*; Bharathram Uppili; Debasis Dash; Hema Gogia; Hemlata Lall; Mahesh S Dhar; Meena Datta; Mitali Mukerji; Mohammed Faruq; Nishu Tyagi; Partha Rakshit*; Pooja Sharma; Pramod Kumar#; Prateek Singh; Preeti Madan; Priyanka Singh; Radhakrishanan VS; Rajesh Pandey#; Robin Marwal; Sandhya Kabra; Saruchi Wadhwa; Sujeet Singh; Uma Sharma; Varun Jaiswal; Vivekanand A
EPI_ISL_410535	National Centre for Infectious Diseases	Programme in Emerging Infectious Diseases, Duke-NUS Medical School	Barnaby E Young; Danielle E Anderson; David CB Lye; Gavin JD Smith; Jayanthi Jayakumar; Martin Linster; Yan Zhuang; Yee Sin Leo; Yvonne CF Su
EPI_ISL_1252721	National Food and Veterinary Risk Assessment Institute	Vilnius University Hospital Santaros Klinikos, Center of Laboratory Medicine	Daniel Naumovas; Dovile Ezerskyte; Gytis Dudas; Ingrida Olendraite; Laimonas Griskevicius; Ligita Raugaite; Mindaugas Stoskus; Monika Katenaite; Rimvydas Norvilas
EPI_ISL_560388	National Health Laboratory	Botswana Institute for Technology Research and Innovation	Dineo Emang Tshiamo. Gape Nyepetsi; Kefentse Arnold Tumedi; Madisa Mine; Maitshwarelo Ignatius Matsheka; Thongbotho Mphoyakgosi
EPI_ISL_560386	National Health Laboratory	Botswana Institute for Technology Research and innovation	Dineo Emang Tshiamo. Gape Nyepetsi; Kefentse Arnold Tumedi; Madisa Mine; Maitshwarelo Ignatius Matsheka; Thongbotho Mphoyakgosi
EPI_ISL_456600	National Health Laboratory, Timor-Leste	Microbiological Diagnostic Unit Public Health Laboratory, The Peter Doherty Institute for Infection and Immunity	Antonia da Costa, E.; Baird, R.; Barreto, I.; Caly, L.; Canisia, D.; Dakh, F.; Dolores de Jesus da Costa, M.; Douglas, N.; Francis, J.; Freeman, K.; Jayanti Pereira Tilman, A.; Marr, I.; Sait, M.; Salles de Sousa, A.; Schultz, M.; Seemann, T.; Sherry, N.; Soares da Silva, E.; Wapling, J.; Ximenes, J.
EPI_ISL_770470	National Health laboratory	Botswana Institute for Technology Research and Innovation	Dineo Emang Tshiamo. Gape Nyepetsi; Kefentse Arnold Tumedi; Madisa Mine; Maitshwarelo Ignatius Matsheka; Malebogo Kebabonye; Thongbotho Mphoyakgosi
EPI_ISL_455695, EPI_ISL_455708, EPI_ISL_511898	National Hospital of Tropical Diseases	Oxford University Clinical Research Unit, Hanoi, Vietnam	H. Rogier van Doorn; Le Nguyen Minh Hoa; Nguyen Thi Hong Thuong; Nguyen Thi Ngoc Diep; Nguyen Thi Tam; Nguyen Thu Trang; Pham Ngoc Thach; Van Dinh Trang; Vu Thi Ngoc Bich; on behalf of the OUCRU COVID-19 research group
EPI_ISL_862077, EPI_ISL_862078, EPI_ISL_1014676, EPI_ISL_1014683, EPI_ISL_1014684, EPI_ISL_1014685	National Influenza Center, Virology Department	National Influenza Center	A Nejati; F Ajaminejad; F Ajaminejad and T Mokhtari Azad; J Yavarian; K Sadeghi; N Ghavvami; N Ghavvami and T Mokhtari Azad; NZ Shafiei Jandaghi; V Salimi
EPI_ISL_402125	National Institute for Communicable Disease Control and Prevention (ICDC) Chinese Center for Disease Control and Prevention (China CDC)	National Institute for Communicable Disease Control and Prevention (ICDC) Chinese Center for Disease Control and Prevention (China CDC)	Chen; Dai; F.-H.; Hu, Y.; J.-H.; J.-J.; J.-L. and Zhu; Liu, Y.; Pei; Q.-M.; She; Song; T.-Y.; Tao; Tian; Wang; Wang, W.; Wu, F.; Xu, L.; Y.-L.; Y.-M.; Y.-Y.; Y.-Z.; Yu, B.; Z.-G.; Z.-W.; Zhang; Zhao, S.; Zheng
EPI_ISL_718264, EPI_ISL_728279	National Institute for Infectious Diseases, INMI, "L. Spallanzani" IRCCS	National Institute for Infectious Diseases, INMI, "L. Spallanzani" IRCCS	A Di Caro; B Bartolini; C.E.M Gruber; E Giombini; F Messina; M Rueca; MR Capobianchi
EPI_ISL_591270, EPI_ISL_591280, EPI_ISL_850949	National Institute for Viral Disease Control and Prevention, China CDC	National Institute for Viral Disease Control and Prevention, China CDC	Cao Chen; Dayan Wang; George F.Gao; Hong Wang; Huilai Ma; Ji Wang; Jingdong Song; Jun Han; Kai Nie; Ruqin Gao; Shiwen Wang; Weimin Zhou; Wenbo Xu; Wenjie Tan; Xiang Zhao; Yang Song; Yanhai Wang; Yao Meng; Yenan Feng; Yong Zhang; Yuchao Wu; Zhaoguo Wang; Zhixiao Chen
EPI_ISL_709542	National Institute of Blood Diseases (NIBD), Molecular Biology Lab	Genomics Lab NIBD	Aneeta Shahni; Arshi Naz; Gul Sufaida; Samina Naz Mukry; Sayed Ali Raza; Shariq Ahmed; Tahir Sultan Shamsi

EPI_ISL_1118215	National Institute of Health Research and Development	National Institute of Health Research and Development	Agustiningsih; Arie Ardiansyah Nugraha; Fauzul Muna; Hana Apsari Pawestri; Hartanti Dian Ikawati; Herna; Holy Arif Wibowo; Irene Lorinda Indalao; Kartika Dewi Puspa; Kindi Adam; Krisna Nur Andriana Pangesti; Natalie Laurencia Kipuw; Nelly Puspandari; Ni Ketut Susilarini; Nike Susanti; Nurika Hariastuti; Reni Herman; Ririn Ramadhany; Subangkit; Tati Febriyanti; Triyani Soekarso; Ully Alfi Nikmah; Vivi Setiawaty.; Yuni Rukminiati
EPI_ISL_1279965	National Institute of Infectious Diseases-Prof. Dr. Matei Bals Molecular Diagnostics Laboratory	National Institute of Infectious Diseases-Prof. Dr. Matei Bals Molecular Diagnostics Laboratory	Andreea Tudor; Corina Casangiu; Dan Otelea; Leontina Banica; Marius Surleac; Petre Milu; Simona Paraschiv
EPI_ISL_475757	National Institute of Laboratory Medicine and Referral Center	Genomic Research Lab, BCSIR	A. K. M. Shamsuzzaman; Abu Sayeed Mohammad Mahmud; Arifa Akram; Asish Kumar Ghosh; Barna Goswami; Eshrar Osman; Iffat Jahan; Mahmuda Yeasmin; Md. Ahasan Habib; Md. Maruf Ahmed Molla; Md. Murshed Hasan Sarkar; Md. Saddam Hossain; Md. Salim Khan; Mohammad Samir Uzzaman; Salek Ahmed Sajib; Shahina Akter; Sheikh Md. Selim Al Din; Tanjina Akhter Banu; Tasnim Nafisa; Utpal Chandra Ray
EPI_ISL_1267023	National Laboratory for Health, Environment and Food, OMM, Maribor	CISLD (Clinical Institute of Special Laboratory Diagnostics), University Children's Hospital, University Medical Center Ljubljana	Ana Grom; Barbara Jenko Bizjan; Jernej Kova; Katarina Kozmos; Marko Pokorn; Maruša Debeljak; Robert Šket; Tadej Battelino; Tine Tesovnik
EPI_ISL_512614, EPI_ISL_512810	National Laboratory for Influenza/Virology reference laboratory, Public Health Center of the Ministry of Health of Ukraine	Respiratory Virus Unit, Microbiology Services Colindale, Public Health England	Dr. Iryna Demchyshyna; PHE Covid Sequencing Team
EPI_ISL_647971, EPI_ISL_1191806, EPI_ISL_1191808, EPI_ISL_1191819, EPI_ISL_1191826, EPI_ISL_1191833, EPI_ISL_1191861			
see above	National Microbiology Reference Laboratory	Quadram Institute Bioscience	Agnes Juru; Alexander Goredema; Ana-Victoria Gutierrez; Andrew J. Page; Andrew Tarupiwa; Barbra Murwira; Beuty Makamure; Charles Nyagupe; David Baker; Faustinos T Takawira; Gaetan Thilliez; Gemma Kay; Gibson Mhlanga; Hlanai Gumbo; Isaac Phiri; Justin O'Grady; Kenneth K Maeka; Leonardo de Oliveira Martins; Muchaneta Mugabe; Portia Manangazira; Raiva Simbi; Robert Kingsley; Sekesai Zinyowera; Tapfumanei Mashe; Tatenda Takawira; Thanh Le Viet
EPI_ISL_1095610, EPI_ISL_1195195, EPI_ISL_1195205, EPI_ISL_1195207	National Public Health Center, COVID Laboratory	National Public Health Center, National Biosafety Laboratory	Bernadett Pályi; Dániel Déri; Judit Henczkó; Norbert Solymosi; Nóra Magyar; Zoltán Kis
EPI_ISL_416885, EPI_ISL_416886	National Public Health Laboratory	Malaysia Genome Institute	Azrin Ahmad; Enizza Kasim; Hani Mat Hussin; Irni Suhayu Sapian; Mohd Faizal Abu Bakar; Mohd Noor Mat Isa; Noorliza Mohamad Noordin; Nor Azfa Johari; Norazimah Tajudin; Nurhezreen Md Iqbal; Rehan Shuhada Abu Bakar; Selvanesan Sengol; Shahrul Hisham Zainal Ariffin; Shamsidar Sopie; Siti Noraini Othman; W Afiza W Mohd Arifin; Yu Kie Chem; Yusuf Muhammad Noor
EPI_ISL_754072	National Public Health Laboratory	Nepal Health Research Council	Meghnath Dhimal; Pradip Gyanwali
EPI_ISL_845550, EPI_ISL_845551, EPI_ISL_845561	National Public Health Laboratory, Cameroon	African Centre of Excellence for Genomics of Infectious Diseases (ACEGID), Redeemer's University	Oluniyi P.E. et al
EPI_ISL_596482, EPI_ISL_645115, EPI_ISL_754079	National Public Health Laboratory, National Centre for Infectious Diseases	National Public Health Laboratory, National Centre for Infectious Diseases	Lin Cui; Raymond Tzer Pin Lin; Sophie Octavia; Tze Minn Mak; Zhenyang Zhou
EPI_ISL_1122419, EPI_ISL_1122420	National Public Health Laboratory, National Centre for Infectious Diseases	National Virology Reference Laboratory	Lin Cui; Raymond Tzer Pin Lin; Surita Taib; Tze Minn Mak; Zainun Zaini; Zhenyang Zhou
EPI_ISL_480302	National Reference Laboratory "Influenza and acute respiratory diseases"	NRL-HIV	Ivailo Alexiev; Ivan Ivanov; Ivva Philipova
EPI_ISL_1273401	National Reference Laboratory - Ministry of Health Maseru Lesotho	National Institute for Communicable Diseases of the National Health Laboratory Service	Amoako DG; Banda R; Bhiman JN; Gorova V; Ismail A; Mahlangu B; Mathabo M; Mohale T; Mooko M; Ntuli N; Scheepers C
EPI_ISL_605073, EPI_ISL_981204	National Virus Reference Laboratory	Irish Coronavirus Sequencing Consortium - Helixworks	Conor Crosbie; Nimesh Pinnamaneni; Sachin Chalapati
EPI_ISL_848159	National Virus Reference Laboratory	Irish Coronavirus Sequencing Consortium - National University of Ireland Galway	Grainne Mc Andrew; Kate Reddington; Simone Coughlan
EPI_ISL_512090, EPI_ISL_875385, EPI_ISL_1357899	National Virus Reference Laboratory	National Virus Reference Laboratory	Aditi Chaturvedi; Charlene Bennet; Cillian F De Gascun; Gabriel Gonzalez; Jonathan Dean; Michael Carr; Suzie Coughlan; Zoe Yandle
EPI_ISL_754068	Nepal Korea Friendship Municipality Hospital	Nepal Health Research Council	Meghnath Dhimal; Pradip Gyanwali
EPI_ISL_487108, EPI_ISL_729946, EPI_ISL_729979, EPI_ISL_729981, EPI_ISL_729985	Nigeria Centre for Disease Control (NCDC)	African Centre of Excellence for Genomics of Infectious Diseases (ACEGID), Redeemer's University, Ede, Osun State, Nigeria	Ajogbasile F.V.; Folarin O.A.; Happi C.T.; Ihekweazu C.; Kayode A.; Oguzie J.; Olawoye I.; Olumade T.; Oluniyi P.E.; Oluniyi P.E. et al; Uwanibe J.
EPI_ISL_775373	Nordland Hospital - Bodo, Laboratory Department, Molecular Biology Unit	Norwegian Institute of Public Health, Department of Virology	Atiya R Ali; Hilde Elshaug; Hilde Vollan; Kamilla Heddeland Instefjord; Karoline Bragstad; Kathrine Stene-Johansen; Marie Paulsen Madsen; Olav Hungnes; Rasmus Riis Kopperud
EPI_ISL_925901, EPI_ISL_960301	Nucleic Acid Testing, National Reference Laboratory	GIGA Medical Genomics	Bouchra Boujemla; Corinne Fasquelle; Esperence Umumararungu; Jacob Souopgui; Keith Durkin; Léon Mutesa; Maria Artesi; Marie-Pierre Hayette; Nathalie Renotte; Patrick Tuyisenge; Robert Rutayisire; Sabin Nsanzimana; Swaibu Gatare; Sébastien Bontems; Vincent Bours; Yvan Butera
EPI_ISL_833078	OSPEDALE CIVILE ATRI	Istituto Zooprofilattico Sperimentale dell'Abruzzo e Molise "G. Caporale"	Ancora M; Calistri P; Cammà C; Curini V; Delli Compagni E; Di Domenico M; Di Pasquale A; Lorusso A; Mangone I; Marcacci M; Puglia I; Rinaldi A; Savini G
EPI_ISL_882795, EPI_ISL_1074002, EPI_ISL_1074024	Office of Diseases Prevention and Control Region 4 Saraburi	COVID-19 Network Investigations (CONI) Alliance	Amornmas Kongklieng; Anek Mungaomklang; Angkana Huang; Anthony R. Jones; Arporn Wangwiwatsin; Bhakbhoom Panthan; Chonticha Klungtong; Duangkamon Loesbanluechai; Ekawat Pasomsub; Elizabeth Batty; Insee Sensorn; Janjira Thaipadungpanit; Jutikul Kaewmalakul; Kamolthip Atsawawaranunt; Khajohn Joonlasak; Khajohn Joonsalak; Kingkan Rakmanee; Krittikorn Kumpornsin; Namfon Kotanan; Nathamon Runnachot; Pakjira Rimdusit; Payon Pengyo; Praima Moonmuang; Prayuth Kaewmalang; Pukkaporn Parnwijitkul; Sataporn Hatsadichart; Sirinapa Singthong; Siriporn Lakesukthom; Siriwan Yaemnimnual; Stefan Fernandez; Suttiruk Changchawai; Thanat Chookajorn; Theerarat Kochakarn; Treewat Watthanachockchai; Vichan Pawun; Wasun Chantratita; Wonvimol Lemprasert; Wudtichai Manasatienkij
EPI_ISL_458121	Oman National Influenza Centre	Department of Microbiology and Immunology-SQUH	Abdulla Balkhair; Ahlam Al-Amri; Aisha Al-Amri; Aisha Al-Busaidi; Amina Al Jardani; Fahad Zadjali; Fatma BaAlawi; Hamida AL Barwani; Hanan Al-kindi; Intisar Al-Shukri; Khulood Al-Mammary; Mohammed Al-Tobi; Samiha Al Kharusi; Samira Al-Maruqi; Zeyana AL-Dahmani
EPI_ISL_457975	Oman-NIC	Oman-NIC	Abdulla Balkhair; Ahlam Al-Amri; Aisha Al-Amri; Aisha Al-Busaidi; Amina Al Jardani; Fahad Zadjali; Fatma BaAlawi; Hamida AL Barwani; Hanan Al-kindi; Intisar Al-Shukri; Khulood Al-Mammary; Mohammed Al-Tobi; Samiha Al Kharusi; Samira Al-Maruqi; Zeyana AL-Dahmani
EPI_ISL_491147	Oman-National Influenza Center	Biotechnology & OMICs Laboratory	Abdul Latif Khan; Adil Al-Wahaibi; Adil Khan; Ahlam Al-Amri; Ahmed Al-Harrasi; Ahmed Al-Rawahi; Aisha Al-Amri; Aisha Al-Busaidi; Amina Al-Jardani; Hanan Al-Kindi; Intisar Al-Shukri; Sajjad Asaf; Samiha Al-Kharusi; Samira Al-Mahruqi; Seif Al-Abri.
EPI_ISL_766569	Oman-National Influenza Center	Oman-National Influenza Center	Aisha Al-Busaidi; Amina Al-Jardani; Hamida Al-Barwani; Hanan Al-Kindi; Intisar Al-Shukri; Laila Al-Balushi; Samiha Al-Kharusi; Samira Al-Mahruqi
EPI_ISL_639921, EPI_ISL_639926	Omsk Research Institute of Natural Focal Infections	WHO National Influenza Centre Russian Federation	Aleksei Vasilenko; Andrey Komissarov; Artem Fadeev; Daria Nashatyreva; Ekaterina Gradoboeva; Ekaterina Savkina; Elena Poleshchuk; Valery Yakimenko
EPI_ISL_648208	Orebro	The Public Health Agency of Sweden	Anna Risberg; Anna-Malin Linde; Karin Tegmark-Wisell; Maria Lind Karlberg; Mattias Haukland; Mia Brytting; Olov Svartstrom; Oskar Karlsson Lindsjo; Petra Edquist; Reza Advani; Sandra Broddesson
EPI_ISL_1040028	Original detection - Virology Unit, Institut Pasteur du Cambodge; Sequencing - US National Institute of Allergy and Infectious Diseases Cambodia	Virology Unit, Institut Pasteur du Cambodge	Chau Darapheak; Chin Savuth; Erik A Karlsson; Jennifer Bohl; Jessica Manning; Kraing Sidonn; Ly Sovann; Sophana Chea; Sreyngim Lay; Veasna Duong; Yi Sengdoeurn
EPI_ISL_788949, EPI_ISL_788951, EPI_ISL_788954, EPI_ISL_788961,	Ospedale "Di Venere"	Beaconlab (Bioinformatics, Evolution and Comparative Genomics lab), Dept of Biosciences, University on Milan	Chiara M; Iacobellis M; Manzari C; Parisi A; Pesole G; Piluscio R; d'Avenia M

EPI_ISL_872022, EPI_ISL_872028			
EPI_ISL_529015	Ospedale "Ss. Annunziata"	Istituto Zooprofilattico Sperimentale dell'Abruzzo e Molise "G.Caporale"	Ancora M; Cammà C; Curini V; Di Domenico M; Di Pasquale A; Lorusso A; Mangone I; Marcacci M; Puglia I; Rinaldi A; Savini G.
EPI_ISL_747462	Ospedale San Bonifacio	Istituto Zooprofilattico Sperimentale delle Venezie	Adelaide Milani; Alessia Schivo; Alice Fusaro; Ambra Pastori; Annalisa Salviato; Antonia Ricci; Bianca Zecchin; Calogero Terregino; Erika Giorgia Quaranta; Isabella Monne
EPI_ISL_794751	Ospedale Vito Fazzi	Istituto Zooprofilattico Sperimentale della Puglia e della Basilicata	Bianco A.; Capozzi L.; Cipolletta D.; Del Sambro L.; Galante D.; Manzulli V; Pace L.; Parisi A.; Rondinone V.
EPI_ISL_1391049	Ospedale di Venere	Istituto Zooprofilattico Sperimentale della Puglia e della Basilicata	Bianco A.; Capozzi L.; Del Sambro L.; Giannico A.; Iacobellis M.; Parisi A.; Ridolfi D.; Simone D.
EPI_ISL_995378	Outre mer	National Reference Center for Viruses of Respiratory Infections, Institut Pasteur, Paris	Angela Brisebarre; Camille Capel; Combe Patrice; Etienne Simon-Lorière; Marion Barbet; Maud Vanpeene; Méline Bizard; Sylvie Behillil; Sylvie van der Werf; Vincent Enouf
EPI_ISL_1069156	PGIMER, Chandigarh	ICMR-NATIONAL INSTITUTE OF VIROLOGY, MICROBIAL CONTAINMENT COMPLEX	Pragya D. Yadav; Rupinder Bakshi
EPI_ISL_1403595	PRESIDIO TERRITORIALE CASOLI CASOLI(CHIETI)	Istituto Zooprofilattico Sperimentale dell'Abruzzo e Molise "G. Caporale"	Ancora M; Calistri P; Cammà C; Curini V; Delli Compagni E; Di Domenico M; Di Pasquale A; Lorusso A; Mangone I; Marcacci M; Puglia I; Rinaldi A; Savini G; Scialabba S
EPI_ISL_596510, EPI_ISL_596564	Palestinian Ministry of Health	Molecular Genetics Lab	Damien Richard; Dana Najjar; Francois Balloux; Hisham Darwish; Husam Sallam; Issa Shtayeh; Lucy van Dorp; Mahmoud Ruzayqat; Nouar Qutob; Osama Najjar; Zaidoun Salah
EPI_ISL_672628	PathWest Laboratory Medicine WA	PathWest Laboratory Medicine WA Microbial Surveillance Unit	PathWest Laboratory Medicine WA Microbial Surveillance Unit
EPI_ISL_591370, EPI_ISL_892410, EPI_ISL_895559, EPI_ISL_896618, EPI_ISL_898071, EPI_ISL_898860, EPI_ISL_900825, EPI_ISL_902692			
see above	Pathogen Genomics Center, National Institute of Infectious Diseases	Pathogen Genomics Center, National Institute of Infectious Diseases	Kentaro Itokawa; Makoto Kuroda; Masanori Hashino; Rina Tanaka; Tsuyoshi Sekizuka
EPI_ISL_421636	Pathology North	Public Health Virology Laboratory	Alyssa Pyke; Amanda De Jong; Andrew Van Den Hurk; Bixing Huang; Carmel Taylor; David Warrilow; Doris Genge; Elisabeth Gamez; Glen Hewitson; Ian Maxwell Mackay; Inga Sultana; Jamie McMahon; Jean Barcelon; Judy Northill; Mitchell Finger; Natalie Simpson; Neelima Nair; Peter Burtonclay; Peter Moore; Sarah Wheatley; Sean Moody; Sonja Hall-Mendelin; Timothy Gardam; and Frederick Moore
EPI_ISL_1081794	Philippine Red Cross	Philippine Genome Center	Alethea R. de Guzman; Anna Ong-Lim; Arianne A. Zamora; Asia Louisa U. Chong; Benedict A. Maralit; Candice Francheska B. Tambaoan; Carlo M. Lapid; Celia Carlos; Cynthia P. Saloma; Devon Ray Pacial; Edsel Maurice Salvaña; El King D. Morado; Eva Maria Cutiongco-de la Paz; Francis A. Tablizo; Irish Coleen A. Asin; Jaime C. Montoya; Jan Michael C. Yap; Jo-Hannah S. Llames; John Q. Wong; Joshua Gregor A. Dizon; Juan Antonio R. Magalang; Karol Sophia Agape R. Padilla; Kenneth M. Kim; Kris P. Punayan; Marc Edsel C. Ayes; Marc Jerrone R. Castro; Maria Sofia L. Yangzon; Marissa Alejandria; Razel Nikka M. Hao; Rianna Patricia S. Cruz; Sheila Mae M. Araiza; and Maria Rosario Singh-Vergeire
EPI_ISL_1180684, EPI_ISL_1180691	Public Health Authority of the Slovak Republic	Bergthaler laboratory, CeMM Research Center for Molecular Medicine of the Austrian Academy of Sciences	Andreas Bergthaler; Anna Schedl; Bekir Erguner; Benedikt Agerer; Christoph Bock; Fabian Amman; Jan Laine; Lukas Endler; Maelle Le Moing; Martin Senekowitsch; Michael Schuster; Thomas Penz
EPI_ISL_1112318, EPI_ISL_1112335, EPI_ISL_1112753	Public Health Center of Ukraine	Charite Universitatsmedizin Berlin, Institute of Virology	Barbara Mühlemann; Christian Drosten; Ihor Kuzin; Iryna Demchyshyna; Julia Schneider; Jörn Beheim-Schwarzbach; Liudmyla Chernenko; Roman Rodyna; Talitha Veith; Terry Jones; Victor M Corman
EPI_ISL_413015	Public Health Ontario Laboratory	National Microbiology Laboratory	Adrian Zetner; Anna Majer; Darryl Falzarano; Erika Landry; Gerdts Volker; Grace Seo; Guillaume Poliquin; Jocelyne Lew; Jonathan Gubbay; Matthew Gilmour; Morag Graham; Natalie Knox; Nathalie Bastien; Philip Mabon; Rob Kozak; Samira Mubareka; Shari Tyson; Stephanie Booth; Timothy Booth; Tom Graefenhan; Yan Li
EPI_ISL_639819	Public Health Virology Laboratory, Forensic and Scientific Services (PHV-FSS)	Public Health Virology Laboratory, Forensic and Scientific Services (PHV-FSS)	Son Nguyen et al
EPI_ISL_1273081	Public Health Virology-Forensic and Scientific Services (PHV-FSS)	Public Health Virology-Forensic and Scientific Services (PHV-FSS)	Son Nguyen
EPI_ISL_513313	Public Health, United States Air Force School of Aerospace Medicine	Public Health, United States Air Force School of Aerospace Medicine	A.C.; A.K.; A.W.; B.C.; C.R.; Chapleau; Connors; E.A.; Fries; J.R.; Javorina; Lambert; Macias; Meyer; Purves; R.R. and Starr; S.M.
EPI_ISL_632936	Puskesmas Mlati 1 Sleman	Genetics Working Group (Pokja Genetik) Faculty of Medicine, Public Health and Nursing Universitas Gadjah Mada (FK-KMK UGM); Disease Investigation Center Wates Ministry of Agriculture Indonesia; Department of Microbiology FK-KMK UGM; Laboratorium Diagnostik Yayasan Tahija World Mosquito Program (WMP) Yogyakarta Center for Tropical Medicine FK-KMK UGM; Integrated Research Center FK-KMK UGM; Department of Computer Science and Electronics FMIPA UGM; RSUP Dr. Sardjito	Afiahayati; Bambang Sigit Riyanto; Dwi AA Nugrahaningsih; Edwin W. Daniwijaya; Eggi Arguni; Eko Budiono; Endah Supriyati; Ernawati; Gunadi; Hendra Wibawa; Heni Retnowulan; Ika Trisnawati; Ira Puspitawati; Kristy Iskandar; Ludhang P. Rizki; Marcellus; Mohamad S. Hakim; Munawar Gani; Nungki Anggorowati; Nur Imma Fatimah Harahap; Nur Rahmi Ananda; Osman Sianipar; Riat El Khair; Satria Maulana; Siswanto; Sumardi; Titik Nuryastuti; Tri Wibawa; Umi Solekhah Intansari; Untung Wirawan; William Widitjiarso; Yunika Puspadewi; Elizabeth Henny Herningtiyas
EPI_ISL_518819	Qadr Hospital, Tangerang, Banten	Biosafety Level-3 Laboratory, Indonesian Institute of Sciences (LIPI)	Ade Andriani; Ahmad Fathoni; Andri Wardiana; Anggia Prasetyoputri; Anik Budhi Dharmayanthi; Ario Betha Juanssilfero; Asep M Ridwanuloh; Isa Nuryana; Puspita Lisdiyanti; Ratih Asmana Ningrum; Rifki Sadikin; Syam Budi Iryanto; Wien Kusharyoto
EPI_ISL_416477	R. G. Lugar Center for Public Health Research, National Center for Disease Control and Public Health (NCDC) of Georgia.	R. G. Lugar Center for Public Health Research, National Center for Disease Control and Public Health (NCDC) of Georgia.	Adam Kotorashvili; Amiran Gamkrelidze.; Ana Papkiauri; Ann Machablishvili; Anna Kasradze; Davit Tsaguria; Ekaterine Khmaladze; Ekaterine Zangaladze; Ekaterine Zhghenti; Giorgi Tomashvili; Gvantsa Brachveli; Gvantsa Chanturia; Irma Burjanadze; Ketevan Sidamonidze; Khatuna Zakhashvili; Lela Sabadze; Lela Urushadze; Magda Dgebuadze; Maia Alkhazashvili; Mari Gavashelidze; Mariam Zakalashvili; Marine Murtskhvaladze; Meri Pantsulaia; Nato Kotaria; Nino Berishvili; Paata Imnadze; Roena Sukhiashvili; Tamar Jashiashvili; Tata Imnadze; Tea Tevdoradze
EPI_ISL_413570	RIVM	Erasmus Medical Center	Anne van der Linden; Annemiek van der Eijk; Aura Timen; Bas Oude Munnink; Claudia Schapendonk; Corien Swaan; Corine GeurtsvanKessel; David Nieuwenhuijse; Irina Chestakova; Jeroen van Kampen; Jolanda Voermans; Madelief Mollers; Manon Haverkate; Marion Koopmans; Mark Pronk; Mart Stein; Pascal Lexmond; Reina Sikkema; Richard Molenkamp; Sandra Kengne Kamga Mobou; on behalf of the Dutch national COVID-19 response team.
EPI_ISL_574607	RS Husada	Eijkman Institute for Molecular Biology, Ministry of Research and Technology/National Agency for Research and Innovation	Amin Soebandrio; David H Muljono; Edison Johar; Frilasita A Yudhaputri; Herawati Sudoyo; Hidayat Trimarsanto; Iskandar A Adnan; Khin Saw Myint; Safarina G Malik; Willy Agustine
EPI_ISL_888991	RS Mitra Keluarga Gading Serpong	Eijkman Institute for Molecular Biology, Ministry of Research and Technology/National Agency for Research and Innovation	Amin Soebandrio; Edison Johar; Frilasita A Yudhaputri; Hidayat Trimarsanto; Iskandar Adnan; Khin Saw Myint; Lydia V. Panggalo; Safarina G Malik; Sukma Oktavianthi; Willy Agustine
EPI_ISL_1117590	RS Trimitra, Bogor, West java	Biosafety Level-3 Laboratory, Indonesian Institute of Sciences (LIPI)	Ade Andriani; Anik Budhi Dharmayanthi; Asep M. Ridwanuloh; Eko Wahyu Putro; Gita Syahputra; Hariyatun; Inswasti Cahyani; Matthew W. Loose; Mohammad Ilyas; Susanti; Syam Budi Iryanto; Wien Kusharyoto
EPI_ISL_454497	RSE "National Center for Biotechnology"	RSE "National Center for Biotechnology"	Akbota Rakhmetova; Alexandr Shevtsov; Askar Abdaliyev; Asylulan Amirgazin; Ilyas Akhmetollayev; Ruslan Kalendar; Viktoriya Lutsay; Yerlan Ramankulov; Zabira Aushakhmetova
EPI_ISL_576113, EPI_ISL_576130	RSUP Dr. Sardjito	Genetics Working Group (Pokja Genetik) Faculty of Medicine, Public Health and Nursing Universitas Gadjah Mada (FK-KMK UGM); Disease Investigation Center Wates Ministry of Agriculture Indonesia; Department of Microbiology FK-KMK UGM; Laboratorium Diagnostik Yayasan Tahija World	. Marcellus; Afiahayati; Alvin S. Kalim; Amalia Setyati; Cahya Dewi Satria; Desyifa Mursalin; Dwi AA Nugrahaningsih; Dwikisworo Setyowireni; Edwin W. Daniwijaya; Eggi Arguni; Elisabeth Siti Herini; Endah Supriyati; Fadil Fahri; Gunadi; Hendra Wibawa; Ida Safitri Laksanawati; Ika Trisnawati; Ira Puspitawati; Kristy Iskandar; Ludhang P. Rizki; Marcellus; Mohamad S. Hakim; Nungki Anggorowati; Nur Imma Fatimah Harahap; Osman Sianipar; Riat El Khair; Rina Triasih; Siswanto; Susan Simanjaya; Titik Nuryastuti; Titis Widowati; Tri Wibawa; Umi Solekhah Intansari; William Widitjiarso; Yunika Puspadewi; Elizabeth Henny Herningtiyas

		Mosquito Program (WMP) Yogyakarta Center for Tropical Medicine FK-KMK UGM; Integrated Research Center FK-KMK UGM; Department of Computer Science and Electronics FMIPA UGM	
EPI_ISL_450511, EPI_ISL_450512, EPI_ISL_450514	Rafik Hariri University Hospital	Rafik Hariri University Hospital	Rita Feghali
EPI_ISL_447000, EPI_ISL_812924	Ramathibodi Hospital	COVID-19 Network Investigations (CONI) Alliance	Angkana Huang; Anthony R. Jones; Arporn Wangwiwatsin; Bhakbhoom Panthan; Chonticha Klungtong; Duangkamon Loesbanluechai; Ekawat Pasomsub; Elizabeth Batty; Insee Sensorn; Janjira Thaipadungpanit; Khajohn Joonsalak; Kingkan Rakmanee; Krittikorn Kumpornsin; Namfon Kotanan; Stefan Fernandez; Thanat Chookajorn; Theerarat Kochakarn; Treewat Watthanachockchai; Wasun Chantratita; Wudtichai Manasatienkij
EPI_ISL_1123264	Rangamati General Hospital RT-PCR lab	Central Biological Research Laboratory and Department of Biochemistry and Molecular Biology	H. M. Abdullah Al Masud; Imam Hossen; Md. Arif Hossain; Md. Imranul Hoq; Md. Khondakar Raziur Rahman; Md. Omer Faruq; Mohammad Omar Faruque; Robiul Hasan Bhuiyan; Sajib Rudra; Shanta Paul
EPI_ISL_491474	Research Institute for Tropical Medicine	Research Institute for Tropical Medicine	Catalino Demetria; Criselda Bautista; Daria Manalo; Edelwisa Mercado; Francisco Gerardo Polotan; Inez Andrea Medado; Kirstyn Brunker; Ma. Angelica Tujan; Othoniel Jan Onza
EPI_ISL_430456	Rizal Medical Center	Research Institute for Tropical Medicine	Bautista; Brunker, K.; C.S.; C.T.; D.L.; Demetria; E.S.; F.G.M.; I.A.P.; Manalo; Medado; Mercado; O.J.T.; Onza; Polotan
EPI_ISL_516806	Rumah Sakit PKU Gamping	Genetics Working Group (Pokja Genetik) Faculty of Medicine, Public Health and Nursing Universitas Gadjah Mada (FK-KMK UGM); Disease Investigation Center Wates Ministry of Agriculture Indonesia; Department of Microbiology FK-KMK UGM; Laboratorium Diagnostik Yayasan Tahija World Mosquito Program (WMP) Yogyakarta Center for Tropical Medicine FK-KMK UGM; Integrated Research center FK-KMK UGM; Department of Computer Science and Electronics FMIPA UGM	. Afiahayati; . Marcellus; . Siswanto; Ardorisye Saptaty Fornia; Dwi AA Nugrahaningsih; Edwin W. Daniwijaya; Eggi Arguni; Endah Supriyati; Gunadi; Hendra Wibawa; Kemala Athollah; Ludhang P. Rizki; Mohamad S. Hakim; Titik Nuryastuti; Tri Wibawa
EPI_ISL_1138512, EPI_ISL_1138514	SA Pärnu Hospital Laboratory	1. Laboratory of Communicable Diseases (Estonia); 2. Eurofins Genomics Europe Sequencing GmbH	Liidia Dotsenko
EPI_ISL_1198837, EPI_ISL_1198841	SARS-CoV-2 testing team, National Institute of Infectious Diseases	Pathogen Genomics Center, National Institute of Infectious Diseases	Daisuke Kobayashi; Hussein H. Aly; Kentaro Itokawa; Kousho Wakae; Makoto Kuroda; Masanori Hashino; Rina Tanaka; Takanobu Kato; Tsuyoshi Sekizuka
EPI_ISL_1036200	SIESP CH	Istituto Zooprofilattico Sperimentale dell'Abruzzo e Molise "G. Caporale"	Ancora M; Calistri P; Cammà C; Curini V; Di Domenico M; Di Pasquale A; Lorusso A; Mangone I; Marcacci M; Puglia I; Rinaldi A; Savini G; Scialabba S
EPI_ISL_961689	SIESP CHIETI - DRIVE IN CHIETI	Istituto Zooprofilattico Sperimentale dell'Abruzzo e Molise "G. Caporale"	Ancora M; Calistri P; Cammà C; Curini V; Di Domenico M; Di Pasquale A; Lorusso A; Mangone I; Marcacci M; Puglia I; Rinaldi A; Savini G; Scialabba S
EPI_ISL_1403560	SIESP CHIETI - DRIVE IN LANCIANO LANCIANO(CHIETI)	Istituto Zooprofilattico Sperimentale dell'Abruzzo e Molise "G. Caporale"	Ancora M; Calistri P; Cammà C; Curini V; Delli Compagni E; Di Domenico M; Di Pasquale A; Lorusso A; Mangone I; Marcacci M; Puglia I; Rinaldi A; Savini G; Scialabba S
EPI_ISL_1058044	SIESP CHIETI - DRIVE IN ORTONA	Istituto Zooprofilattico Sperimentale dell'Abruzzo e Molise "G. Caporale"	Ancora M; Calistri P; Cammà C; Curini V; Di Domenico M; Di Pasquale A; Lorusso A; Mangone I; Marcacci M; Puglia I; Rinaldi A; Savini G; Scialabba S
EPI_ISL_918264	SIESP CHIETI-DRIVE IN CHIETI	Istituto Zooprofilattico Sperimentale dell'Abruzzo e Molise "G. Caporale"	Ancora M; Calistri P; Cammà C; Curini V; Di Domenico M; Di Pasquale A; Lorusso A; Mangone I; Marcacci M; Puglia I; Rinaldi A; Savini G; Scialabba S
EPI_ISL_883288	SIESP CHIETI-DRIVE IN ORTONA	Istituto Zooprofilattico Sperimentale dell'Abruzzo e Molise "G. Caporale"	Ancora M; Calistri P; Cammà C; Curini V; Di Domenico M; Di Pasquale A; Lorusso A; Mangone I; Marcacci M; Puglia I; Rinaldi A; Savini G; Scialabba S
EPI_ISL_918263	SIESP DIPARTIMENTO DI PREVENZIONE CHIE	Istituto Zooprofilattico Sperimentale dell'Abruzzo e Molise "G. Caporale"	Ancora M; Calistri P; Cammà C; Curini V; Di Domenico M; Di Pasquale A; Lorusso A; Mangone I; Marcacci M; Puglia I; Rinaldi A; Savini G; Scialabba S
EPI_ISL_833303, EPI_ISL_1336893	SIESP DIPARTIMENTO DI PREVENZIONE TERAMO	Istituto Zooprofilattico Sperimentale dell'Abruzzo e Molise "G. Caporale"	Ancora M; Calistri P; Cammà C; Caporale M; Curini V; Di Domenico M; Di Lollo Valeria; Di Pasquale A; Lorusso A; Mangone I; Marcacci M; Puglia I; Rinaldi A; Savini G; Scialabba S
EPI_ISL_833112	SIESP DIPARTIMENTO DI PREVENZIONE TERAMO C.DA CASALENA	Istituto Zooprofilattico Sperimentale dell'Abruzzo e Molise "G. Caporale"	Ancora M; Calistri P; Cammà C; Curini V; Delli Compagni E; Di Domenico M; Di Pasquale A; Lorusso A; Mangone I; Marcacci M; Puglia I; Rinaldi A; Savini G
EPI_ISL_961758	SIESP SULMONA	Istituto Zooprofilattico Sperimentale dell'Abruzzo e Molise "G. Caporale"	Ancora M; Calistri P; Cammà C; Curini V; Di Domenico M; Di Pasquale A; Lorusso A; Mangone I; Marcacci M; Puglia I; Rinaldi A; Savini G; Scialabba S
EPI_ISL_1357435	SYNLAB Labor München Zentrum LMZ	Robert Koch Institute	
EPI_ISL_1286866	SYNLAB MVZ Trier	Robert Koch Institute	
EPI_ISL_1257869, EPI_ISL_1257872	San Gallicano Dermatological Institute I.F.O.	San Gallicano Dermatological Institute I.F.O.	; Blandino G; D'Agosto G; Di Domenico EG; Donzelli S; Ensoli F; Federico A; Maione F; Mastrofrancesco A; Morrone A; Orlandi G; Petrolo S; Pimpinelli F.; Ricca V; Spinella F; Strano S
EPI_ISL_542336, EPI_ISL_542345, EPI_ISL_542355, EPI_ISL_542389	San Matteo Hospital Pavia	Dep. Of Oncology and Hemato-Oncology University of Milan	Antonio Piralla; Carlo Federico Perno; Chiara Vismara; Claudia Alteri; Elisa Matarazzo; Fausto Baldanti; Federica Giardina; Federica Novazzi; Luna Colagrossi; Maria Antonello; Massimo Puoti; Monica Tallarita; Oscar Massimiliano Epis; Roberto Fumagalli; Silvia Renica; Stefano Gaiarsa; Valentino Costabile; Valeria Cento
EPI_ISL_654496	Servicio de Microbiología. Hospital Clínico Universitario de Valencia	SeqCOVID-SPAIN consortium/IBV(CSIC)	David Navarro Ortega; Eliseo Albert Vicent; Ignacio Torres and SeqCOVID-SPAIN consortium
EPI_ISL_654478	Servicio de Microbiología. Hospital General Universitario de Castellón	SeqCOVID-SPAIN consortium/IBV(CSIC)	María Dolores Tirado Balaguer and SeqCOVID-SPAIN consortium; Rosario Moreno Muñoz
EPI_ISL_849657, EPI_ISL_849661	Servizio Igiene Epidemiologia e Sanità Pubblica (SIESP)-L'Aquila	Istituto Zooprofilattico Sperimentale dell'Abruzzo e Molise "G.Caporale"	Ancora M; Cammà C; Curini V; Di Domenico M; Di Pasquale A; Lorusso A; Mangone I; Marcacci M; Puglia I; Rinaldi A; Savini G.
EPI_ISL_849655	Servizio di igiene e sanità pubblica (SIESP)-Teramo	Istituto Zooprofilattico Sperimentale dell'Abruzzo e Molise "G.Caporale"	Ancora M; Cammà C; Curini V; Di Domenico M; Di Pasquale A; Lorusso A; Mangone I; Marcacci M; Puglia I; Rinaldi A; Savini G.
EPI_ISL_528994, EPI_ISL_528996, EPI_ISL_529003, EPI_ISL_529005, EPI_ISL_529024, EPI_ISL_529025, EPI_ISL_849662			
see above	Servizio di igiene epidemiologia e sanità pubblica (SIESP)-Chieti	Istituto Zooprofilattico Sperimentale dell'Abruzzo e Molise "G.Caporale"	Ancora M; Cammà C; Curini V; Di Domenico M; Di Pasquale A; Lorusso A; Mangone I; Marcacci M; Puglia I; Rinaldi A; Savini G.
EPI_ISL_1251082, EPI_ISL_1251089	Siesp dipartimento di prevenzione	Istituto Zooprofilattico Sperimentale dell'Abruzzo e Molise "G. Caporale"	Ancora M; Calistri P; Cammà C; Curini V; Di Domenico M; Di Pasquale A; Lorusso A; Mangone I; Marcacci M; Puglia I; Rinaldi A; Savini G; Scialabba S
EPI_ISL_482683	Singapore General Hospital	Department of Microbiology	Chenhao Li; Karrie Ko; Kern Rei Chng; Kian Sing Chan; Kun Lee Lim; Lynette Oon; Niranjan Nagarajan; Nurdyana Abdul Rahman
EPI_ISL_768523	Singburi Hospital	National Institute of Health, Department of Medical Sciences, Ministry of Public Health, Thailand	; Natchaya Khiadsang; Pakorn Piromtong; Pilailuk Okada; Ratana Tacharoenmuang; Siripaporn Phuygun; Sittiporn Parnmen; Sunthareeya Waicharoen; Thanutsapa Thanadachakul; Warawan Wongboot; sirikanda wimol
EPI_ISL_882771	Siti Khodijah Hospital	Institute of Tropical Disease, Universitas Airlangga	Aldise M Nastri; Gatot Soegiarto; Jezzy R Dewantari; Kazufumi Shimizu; Krisnoadi Rahardjo; Laksmi Wulandari; Maria I Lusida; Muhammad Hamdan; Resti Yudhawati; Rima R Prasetya; Soetjipto; Yasuko Mori

EPI_ISL_475140	Skovde/Unilabs	The Public Health Agency of Sweden	Anna Risberg; Anna-Malin Linde; Karin Tegmark-Wisell; Maria Lind Karlberg; Mattias Haukland; Olov Svartstrom; Oskar Karlsson Lindsjo; Petra Edquist; Reza Advani; Sandra Broddesson; Shamam Muradrasoli
EPI_ISL_1098651	South Eastern Area Laboratory Services (SEALS)	NSW Health Pathology - Institute of Clinical Pathology and Medical Research; Westmead Hospital; University of Sydney	CIDM-PH et al.
EPI_ISL_849738	Special Operations Medical Research Division, Defence Services Medical Research Centre	Special Operations Medical Research Division, Defence Services Medical Research Centre	Aung; Htun; K.K.; K.Z.; Lwin; Myint, K.; N.M.; Oo; P.K.; Win; Z.W.; Zaw, T.
EPI_ISL_491071	Suceava County Emergency Hospital	"Stefan cel Mare" University Metagenomics Lab	Lobiuc Andrei et al.
EPI_ISL_1259062	Synlab Eesti OÜ	1. Laboratory of Communicable Diseases (Estonia); 2. Eurofins Genomics Europe Sequencing GmbH	Liidia Dotsenko et al.
EPI_ISL_914116, EPI_ISL_914523	TGen North	TGen North	"Jolene Bowers; Ashlyn Pfeiffer; Chris French; Darrin Lemmer; Dave Engelthaler; Hayley Yaglom; Megan Folkerts; The Arizona COVID Genomics Union (ACGU)"
EPI_ISL_966940	Technical Support Units for Scientific Research (UATRS), National Centre for Scientific and Technical Research (CNRST)	Technical Support Units for Scientific Research (UATRS), National Centre for Scientific and Technical Research (CNRST)	Alaoui; Elalaoui; Elannaz, H.; Elouanass, M.; Ennibi; H. and El Fahime, E.; Hemlali, M.; Lahlou; M.A.; Melloul, M.; Rfaki, A.; S.A.; Touil, N.; a.I.
EPI_ISL_756367	The Caribbean Public Health Agency	Carrington Lab, Department of PreClinical Sciences, Faculty of Medical Sciences, The University of the West Indies	; Adesh Ramsubhag; Arianne Brown-Jordan; Avery Hinds; Chinna Chinnadurai; Christine V. F. Carrington; Christopher Oura; Gabriel Escobar; Jacquiline Bissor-McKenzie; Jaya Jayaraman; Jerome Foster; Karla Georges; Marsha Ivey; Naresh Nandram; Nikita S. D. Sahadeo; Nuno Faria; Oliver Pybus; Rahul Naidu; Rajini Haraksingh; Risha Singh; Sarah Hill; Stanley Giddings; SueMin Nathaniel; Vernie Ramkissoon
EPI_ISL_417765, EPI_ISL_828543, EPI_ISL_829364, EPI_ISL_829414	The National University Hospital of Iceland	deCODE genetics	Agnar Helgason; Alma Moller; Arna B Agustsdottir; Arnaldur Gylfason; Asgeir Sigurdsson; Aslaug Jonasdottir; Berglind Eiriksdottir; Bjarni Thorbjornsson; Brynjar O Jensson; Daniel F Gudbjartsson; Droplaug N Magnusdottir; Elisabet E Gardarsdottir; Emil A Thorarensen; Gardar Sveinbjornsson; Gisli Masson; Gudmundur Georgsson; Gudmundur L Norddahl; Gudrun Sigmundsdottir; Hakon Jonsson; Hannes Eggertsson; Hilma Holm; Ingileif Jonsdottir; Jona Saemundsdottir; Kamilla S Josefsdottir; Kari Stefansson; Karl G Kristinsson; Kjartan R Gudmundsson; Kristin E Sveinsdottir; Kristin E Sveinsdottir; Louise le Roux; Maney Sveinsdottir; Olafia S Gretarsdottir; Olafia S Gretarsdottir; Olafur T Magnusson; Pall Melsted; Patrick Sulem; Run Fridriksdottir; Solvi Rognvaldsson; Thora R Gunnarsdottir; Thordur Kristjansson; Thorolfur Gudnason; Unnur Thorsteinsdottir
EPI_ISL_891230	The Oncology Institute "Prof. Dr. Ion Chiricuta" Cluj Napoca	"Stefan cel Mare" University Metagenomics Lab	Gheorghita Roxana; Lobiuc Andrei
EPI_ISL_754231	The Republican Research and Practical Center for Epidemiology and Microbiology (RRPCEM)	WHO National Influenza Centre Russian Federation	Anatoly Krasko; Andrey Komissarov; Anna Ivanova; Artem Fadeev; Daria Danilenko; Dmitry Bazhenov; Dmitry Lioznov; Elena Gasich; Elena Nabieva; Georgii Bazykin; Kirill Bulda; Ksenia Safina; Kseniya Komissarova
EPI_ISL_685975	Tokyo Metropolitan Institute of Public Health	Pathogen Genomics Center, National Institute of Infectious Diseases	Kentaro Itokawa; Makoto Kuroda; Masanori Hashino; Rina Tanaka; Tsuyoshi Sekizuka
EPI_ISL_1260919	ULSS 03 Venezia	Istituto Zooprofilattico Sperimentale delle Venezie	Adelaide Milani; Alessia Schivo; Alice Fusaro; Ambra Pastori; Annalisa Salviato; Antonia Ricci; Calogero Terregino; Edoardo Giussani; Erika Giorgia Quaranta; Isabella Monne; Luca Tassoni
EPI_ISL_977154, EPI_ISL_1260875, EPI_ISL_1260878	ULSS 2 Treviso	Istituto Zooprofilattico Sperimentale delle Venezie	Adelaide Milani; Alessia Schivo; Alice Fusaro; Ambra Pastori; Annalisa Salviato; Antonia Ricci; Bianca Zecchin; Calogero Terregino; Edoardo Giussani; Erika Giorgia Quaranta; Isabella Monne; Luca Tassoni
EPI_ISL_747480, EPI_ISL_747487	ULSS 5 Polesana	Istituto Zooprofilattico Sperimentale delle Venezie	Adelaide Milani; Alessia Schivo; Alice Fusaro; Ambra Pastori; Annalisa Salviato; Antonia Ricci; Bianca Zecchin; Calogero Terregino; Erika Giorgia Quaranta; Isabella Monne
EPI_ISL_733000	UMMC-Health	WHO National Influenza Centre Russian Federation	Andrey Komissarov; Anna Ivanova; Artem Fadeev; Daria Danilenko; Dmitry Bazhenov; Dmitry Lioznov; Elena Nabieva; Georgii Bazykin; Ksenia Safina; Kseniya Komissarova; Tatiana Platonova
EPI_ISL_583963	UOC Microbiologia e Virologia, Azienda Ospedaliera Universitaria Senese, Siena, Italy	Dipartimento di Biotecnologie Mediche	Claudia Gandolfo; David Pinzauti; Francesco Santoro; Gabriele Anichini; Gianni Pozzi; Maria Grazia Cusi
EPI_ISL_1303547	UPA Vila Santa Catarina	Instituto Adolfo Lutz, Interdiciplinary Procedures Center, Strategic Laboratory	Caio Vinicius Dias Lopes; Claudia Regina Gonçalves; Claudio Tavares Sacchi; Erica Valessa Ramos Gomes; Karoline Rodrigues Campos
EPI_ISL_737958, EPI_ISL_737961, EPI_ISL_737962	Uganda Central Public Health Lab and Uganda Virus Research Institute	MRC/UVRI & LSHTM Uganda Research Unit	Dan Lule Bugembe; Matthew Cotten; My V.T. Phan; Pontiano Kaleebu et al.
EPI_ISL_1299859, EPI_ISL_1299874	Unit of lab surveillance of viral emerging diseases, National Lab of Influenza	Respiratory Virus Unit, National Infection Service, Public Health England	Iris Hasibra; PHE Covid Sequencing Team; Prof Albana Fico; Prof Silvia Bino
EPI_ISL_570008	Unity Health Toronto	Ontario Institute for Cancer Research	Bernard Lam; Felicia Vincelli; Ilinca Lungu; Jared T. Simpson; Jeremy Johns; Karel Boissinot; Larissa M. Matukas; Le Luu; Mark Downing; Paul Krzyzanowski; Philip Zuzarte; Ramzi Fattouh; Richard de Borja; Samira Mubareka; TIBDN; Trina Otterman; Wai Sum Siu; Yan Chen; Zhi Cui
EPI_ISL_812967	University Clinical Research Center, University of Sciences	University Clinical Research Center, University of Sciences	A.A.; Bane, S.; Dao, S.; Diakite, M.; Diarra, B.; Doumbia, S.; Guindo, I.; Iknane; Kone, A.
EPI_ISL_710551, EPI_ISL_710572	University Hospital Dubrava	Ruer Boškovic Institute; Forensic Science Centre Ivan Vueti; University of Zagreb Faculty of Science	Ana Livun; Antonela Blažekovi; Boris Maek; Danilo Licastro; Dunja Glavaš; Fran Boroveki; Fuad osovi; Gordana Maravi Vlahoviek; Ivan Šamija; Ivana elap; Jasna Kašman; Josipa Skelin; Katarina Marija Tupek; Kristian Vlahoviek; Kristina Gotovac Jerei; Lidija Cvetko-Krajinovi; Lucija Basi; Lucija Markulin; Maja Kuzman; Marina Korolija; Mario Štefanovi; Mirjana Domazet-Lošo; Paula Štancl; Petra Vrabec; Robert Beluži; Rosa Karli; Sanja Tadinac; Senica Pejša; Tomislav Domazet-Lošo; Valentina umlijan-Combaj; Vjekoslav Tomai; Vladimir Krajinovi; Željka Maak Šafranko
EPI_ISL_1166735	University Hospitals of Geneva, Laboratory of Virology	HUG, Laboratory of Virology and the Health2030 Genome Center	Ana Rita Goncalves; Deborah Penet; Emmanouil Dermitzakis; Henri Pegeot; Ioannis Xenarios; Keith Harshman; Laurent Kaiser; Lorenzo Cerutti; Melyssa Elies; Samuel Cordey
EPI_ISL_775644, EPI_ISL_960043	University Medical Center Hamburg Eppendorf	Heinrich Pette Institute, Leibniz Institute for Experimental Virology	Adam Grundhoff; Alexis Robitaille; Johannes Knobloch; Martin Aepfelbacher; Nicole Fischer; Thomas Günther
EPI_ISL_1072985	University of Balamand	Microbial Genomics Lab, Lebanese American University, Byblos	Mira El Chaar; Sima Tokajian; Youssef Bassim
EPI_ISL_738144	University of Bari Biomedical Sciences and Human Oncology	University of Bari Biomedical Sciences and Human Oncology	Anna Sallustio; Daniela Loconsole; Maria Chironna; Marisa Accogli
EPI_ISL_730652	University of Bari, Valenzano, Italy	Dept. Food safety, Nutrition and Veterinary Public Health, Istituto superiore di sanità	C. Buonavoglia; C. Desario; D. Buonavoglia, V.; E. Lorusso; G. Elia; G. Vaccari; I. Di Bartolo; L. De Sabato; Martella; N. Decaro; U. Agrimi
EPI_ISL_671453, EPI_ISL_671484	University of Debrecen, Department of Medical Microbiology	National Laboratory of Virology, Szentágothai Research Centre	Balázs Somogyi; Brigitta Zana; Endre Gábor Tóth; Eszter Csoma; Ferenc Jakab; Gábor Kemenesi
EPI_ISL_540447	University of Liège COVID-19 testing center	GIGA Medical Genomics	Emmanuel André; Fabrice Bureau; Keith Durkin; Laurent Gillet; Marc Van Ranst; Maria Artesi; Vincent Bours; Wouter Coppieters
EPI_ISL_896375	University of Medicine and Pharmacy of Craiova	"Stefan cel Mare" University Metagenomics Lab	Gheorghita Roxana; Lobiuc Andrei
EPI_ISL_677733, EPI_ISL_677809	University of Szeged, Institute of Clinical Microbiology	National Laboratory of Virology, Szentágothai Research Centre	Balázs Somogyi; Brigitta; Endre Gábor Tóth; Ferenc Jakab; Gabriella Terhes; Gábor Kemenesi
EPI_ISL_779332	University of Wisconsin-Madison AIDS Vaccine Research Laboratories	University of Wisconsin-Madison AIDS Vaccine Research Laboratories	Gage Moreno; Katarina Braun; et al. AIDS Vaccine Research Laboratories
EPI_ISL_977362, EPI_ISL_977365, EPI_ISL_977395, EPI_ISL_977421, EPI_ISL_977427	University of Zambia, School of Veterinary Medicine	UNZAVET and PATH	Daniel Bridges; Mulenga Mwenda-Chimfwembe; Ngonda Saasa

EPI_ISL_1173974	Università Federico II - Dipartimento di scienze mediche traslazionali - Napoli	TIGEM	Antonio Grimaldi Patrizia Annunziata Francesco Panariello Michele Cennamo Valentina Bouche Chiara Colantuono Lucio Di Filippo Mariano Fiorenza Anna Manfredi Marcello Salvi Giuseppe Portella Andrea Ballabio Davide Cacchiarelli
EPI_ISL_1321737	Università degli Studi di Perugia	Istituto Zooprofilattico Sperimentale dell'Abruzzo e Molise "G. Caporale"	Ancora M; Calistri P; Camilloni B; Cammà C; Curini V; Di Domenico M; Di Pasquale A; Lorusso A; Mangone I; Marcacci M; Mencacci A; Puglia I; Rinaldi A; Savini G; Scialabba S
EPI_ISL_1318050	Vestfold Hospital, Toensberg Department of Microbiology	Norwegian Institute of Public Health, Department of Virology	Atiya R Ali; Debech Nadia; Engebretsen Serina Beate; Garcia Llorente Ignacio; Hilde Elshaug; Hilde Vollan; Jon Bråte; Kamilla Heddeland Instefjord; Karoline Bragstad; Kathrine Stene-Johansen; Marie Paulsen Madsen; Olav Hungnes; Pedersen Benedikte Nevjen; Rasmus Riis Kopperud
EPI_ISL_904007	Veterinary Specialized Institute Kraljevo	Veterinary Specialized Institute "Kraljevo", Serbia	Afonso, C.; Banovic Djeri, B.; Jankovic, M.; Jovanovic, T.; Knezevic, A.; Petrovic, T.; Sekler, M.; Tesovic, B.; Vidanovic, D.; Volkening, J.
EPI_ISL_802857	Vilnius University Hospital Santaros Klinikos, Vilnius University	Institute of Biotechnology, Life Sciences Center, Vilnius University	Albertas Timinskas; Alma Gedvilaite; Aurelija Zvirbliene; Daniel Naumovas; Emilija Vasiliunaite; Laimonas Griskevicius; Milda Norkiene
EPI_ISL_560405	Vilnius University Hospital Santaros Klinikos, Vilnius University	Institute of Biotechnology, Life Sciences Center, Vilnius University and Thermo Fisher Scientific	Albertas Timinskas; Alma Gedvilaite; Aurelija Zvirbliene; Daniel Naumovas; Justinas Slikas; Laimonas Griskevicius; Ligita Jancioriene; Mindaugas Paulauskas
EPI_ISL_914886	Vilnius university hospital Santaros Klinikos, Center of Laboratory Medicine	Vilnius University Hospital Santaros Klinikos	Daniel Naumovas; Dovil Ežerskyt; Ingrida Olendraite; Justinas Šlikas; Rimvydas Norvilas
EPI_ISL_934196	Vilnius university hospital Santaros Klinikos, Center of Laboratory Medicine	Vilnius university hospital Santaros Klinikos, Center of Laboratory Medicine	Daniel Naumovas; Dovile Ezerskyte; Gytis Dudas; Ingrida Olendraite; Justinas Slikas; Rimvydas Norvilas
EPI_ISL_467014, EPI_ISL_510754, EPI_ISL_1131002, EPI_ISL_1259894, EPI_ISL_1361488	Viollier AG	Department of Biosystems Science and Engineering, ETH Zürich	Andrea Patrignani; Andreia Cabral de Gouvea; Catharine Aquino; Chaoran Chen; Christian Beisel; Christiane Beckmann; Christoph Noppen; David Dreifuss; Deborah Penet; Doris Popovic; Elodie Burcklen; Emmanouil Dermitzakis; Griffin White; Henri Pegeot; Ina Nissen; Ioannis Xenarios; Ivan Topolsky; Jay Tracy; Katharina Jahn; Keith Harshman; Lara Fuhrmann; Laura Neff; Lennart Opitz; Lorenzo Cerutti; Maria Domenica Moccia; Maurice Redondo; Mirjam Feldkamp; Natascha Santacroce; Niko Beerenwinkel; Noemie Santamaria de Souza; Olivier Kobel; Pedro Ferreira; Philipp Jablonski; Ralph Schlapbach; Rebecca Denes; Sarah Nadeau; Simon Grüter; Sophie Seidel; Susana Posada-Céspedes; Tanja Stadler; Timothy Sykes; Tobias Schär
EPI_ISL_420841, EPI_ISL_447234, EPI_ISL_591083, EPI_ISL_591085, EPI_ISL_961011	Viral Respiratory Lab, National Institute for Biomedical Research (INRB)	Pathogen Sequencing Lab, National Institute for Biomedical Research (INRB)	Allison Black; Amuri Aziza; Andrew Rambaut; Catherine Pratt; Eddy Kinganda-Lusamaki; Edith Nkwembe; Emmanuel Lokilo Lofiko; Francisca Muyembe Mawete; Ian Goodfellow; James Hadfield; Jean Claude Makangara; Jean-Claude Makangara Cigolo; Jean-Jacques Muyembe Tamfum; Josh Quick; Kristian Andersen; Matthias Pauthner; Michael Wiley; Nick Loman; Placide Mbala-Kingebeni; Steve Ahuka-Mundeke; Trevor Bedford
EPI_ISL_1363102	Virological laboratory FBIH "The Center for Hygiene and Epidemiology in the Khabarovsk Region"	Group of Genomics and Postgenomic Technologies of Central Research Institute of Epidemiology	Akimkin VG; Bulanenko VP; Golubeva AG; Kaptelova VV; Kondrasheva LY; Korneenko EV; Lebedeva LA; Lukyanov AV; Saenko SS; Samoilov AE; Savosina LV; Shubina YA; Speranskaya AS; Tivanova EV; Valdokhina AV; Zhaleyko ZP
EPI_ISL_439225	Virology Department, Royal Infirmary of Edinburgh, NHS Lothian / School of Biological Sciences, University of Edinburgh / Institute of Genetics and Molecular Medicine, University of Edinburgh	COVID-19 Genomics UK (COG-UK) Consortium	Balcaza C; Colquhoun R; Dewar R; Gallagher M; Hill V; Jackson B; McCrone JT; McHugh M; O'ÄôToole Å; Rambaut A; Rooke S; Scher E; Templeton K; Williams TC; Yu X
EPI_ISL_1191604	Virology Department, Victoria Hospital, Plaine-Wilhems, Mauritius	National Institute for Communicable Diseases of the National Health Laboratory Service	Amoako DG; Baboo SB; Bhiman JN; Ismail A; Mahlangu B; Manraj SS; Mohale T; Ntuli N; Ramuth M; Scheepers C; Sonoo J
EPI_ISL_856870	Virology Laboratory, Scientific Department, Army Medical Center	Virology Laboratory, Scientific Department, Army Medical Center	Anella Monte; Anna Anselmo; Antonella Fortunato; Filippo Molinari; Florigio Lista; Francesco Giordani; Giancarlo Petralito; Giovanni Faggioni; Nino D'Amore; Riccardo De Sanctis; Silvia Fillo; Vanessa Vera Fain
EPI_ISL_625456, EPI_ISL_677634	Virology Unit, Institut Pasteur de Madagascar	Virology Unit, Institut Pasteur de Madagascar	Cara Brook; Cara E. Brook; Christian Ranaivoson; Cristina M. Tato; Helisoa Razafimanjato; Jean-Michel Heraud; Joseph L. DeRisi; Michelle Tan; Norosoa Razanajatovo; Philippe Dussart; Soa Fy Andriamandimby; Tsiry Randriambolamanantsoa; Vida Ahyong; Vololoniaina Raharinosy
EPI_ISL_918366, EPI_ISL_1098606	Virology Unit, Institut Pasteur du Cambodge	Virology Unit, Institut Pasteur du Cambodge	Chau Darapheak; Chin Savuth; Erik A Karlsson; Etienne Simon-Loriere; Kraing Sidonn; Ly Sovann; Sokhoun Yann; Veasna Duong; Yi Sengdoeurn
EPI_ISL_507286	WHO National Influenza Centre Russian Federation	WHO National Influenza Centre Russian Federation	Andrey Komissarov; Anna Ivanova; Artem Fadeev; Daria Danilenko; Mariia Sergeeva
EPI_ISL_860814, EPI_ISL_861456	WHO/Minsk	Charité Universitätsmedizin Berlin, Institut für Virologie	Barbara Mühlemann; Christian Drosten; Julia Schneider; Julia Tesch; Jörn Beheim-Schwarzbach; Shmialiova Natallia; Sivets Natallia; Talitha Veith; Terry Jones; Tobias Bleicker; Victor M Corman
EPI_ISL_417083, EPI_ISL_417154	Washington State Department of Health	Seattle Flu Study	Chu etl al
EPI_ISL_1255106, EPI_ISL_1255108, EPI_ISL_1255138, EPI_ISL_1255153, EPI_ISL_1255170, EPI_ISL_1255200, EPI_ISL_1255234, EPI_ISL_1255252, EPI_ISL_1255270			
see above	West African Centre for Cell Biology of Infectious Pathogens (WACCBIP), University of Ghana, Accra, Ghana	West African Centre for Cell Biology of Infectious Pathogens (WACCBIP), University of Ghana, Volta Road, Legon-Accra, Ghana	; Abdoulaye B Diallo; Abdul-Karim Abass; Aisha Mohammed; Benjamin Demah Nuertey; Collins M. Morang'a; Dam Kenneth Mibut; Dominic S.Y. Amuzu; Emmanuella Amoako4; Evelyn B. Quansah; Frederick Kumi-Ansah; Frederick Tei-Maya; Gordon A Awandare; Joyce M. Ngoi; Kesego Tapela; Lucas N. Amenga-Etego; Nelson Kibinge; Oliver D Boakye5; Peter K Quashie; Philip M. Soglo; Samirah Saiid; Samuel Kaba Akoriyea; Theophilus Odoom; Vanessa Magnussen; Vincent Appiah; Yaw Bediako
EPI_ISL_1281543	amedes MVZ DIAMEDIS Sennestadt	Robert Koch Institute	
EPI_ISL_1149360	amedes MVZ für Laboratoriumsdiagnostik Raubling GmbH	Robert Koch Institute	
EPI_ISL_827259, EPI_ISL_827393, EPI_ISL_829280	deCODE genetics	deCODE genetics	Agnar Helgason; Alma Moller; Arna B Agustsdottir; Arnaldur Gylfason; Asgeir Sigurdsson; Aslaug Jonasdottir; Berglind Eiriksdottir; Bjarni Thorbjornsson; Brynjar O Jensson; Daniel F Gudbjartsson; Droplaug N Magnusdottir; Elisabet E Gardarsdottir; Emil A Thorarensen; Gardar Sveinbjornsson; Gisli Masson; Gudmundur Georgsson; Gudmundur L Norddahl; Gudrun Sigmundsdottir; Hakon Jonsson; Hannes Eggertsson; Hilma Holm; Ingileif Jonsdottir; Jona Saemundsdottir; Kamilla S Josefsdottir; Kari Stefansson; Karl G Kristinsson; Kjartan R Gudmundsson; Kristin E Sveinsdottir; Louise le Roux; Maney Sveinsdottir; Olafia S Gretarsdottir; Olafur T Magnusson; Pall Melsted; Patrick Sulem; Run Fridriksdottir; Solvi Rognvaldsson; Thora R Gunnarsdottir; Thordur Kristjansson; Thorolfur Gudnason; Unnur Thorsteinsdottir
EPI_ISL_415649	unknown	National Reference Center for Viruses of Respiratory Infections, Institut Pasteur, Paris	Angela Brisebarre; Flora Donati Vincent Enouf; Marion Barbet; Maud Vanpeene; Méline Bizard; Mélnie Albert; Sylvie Behillil; Sylvie van der Werf
