# Supplementary material for: COVseq is a cost-effective workflow for mass-scale SARS-CoV-2 genomic surveillance
Source: Nat Commun. 2021 Jun 23;12:3903. doi: 10.1038/s41467-021-24078-9 (PMC8222401; doi:10.1038/s41467-021-24078-9)
Supplement: Supplementary file 10 — Reporting Summary [file 41467_2021_24078_MOESM10_ESM.pdf]

nature research

Corresponding author(s): Valeria Ghisetti & Nicola Crosetto

Last updated by author(s): May 22, 2021

Reporting Summary

Nature Research wishes to improve the reproducibility of the work that we publish. This form provides structure for consistency and transparency in reporting. For further information on Nature Research policies, see our Editorial Policies and the Editorial Policy Checklist.

Statistics

For all statistical analyses, confirm that the following items are present in the figure legend, table legend, main text, or Methods section.

n/a	Confirmed	
☐	☒	The exact sample size (*n*) for each experimental group/condition, given as a discrete number and unit of measurement
☐	☒	A statement on whether measurements were taken from distinct samples or whether the same sample was measured repeatedly
☐	☒	The statistical test(s) used AND whether they are one- or two-sided<br>*Only common tests should be described solely by name; describe more complex techniques in the Methods section.*
☒	☐	A description of all covariates tested
☒	☐	A description of any assumptions or corrections, such as tests of normality and adjustment for multiple comparisons
☐	☒	A full description of the statistical parameters including central tendency (e.g. means) or other basic estimates (e.g. regression coefficient) AND variation (e.g. standard deviation) or associated estimates of uncertainty (e.g. confidence intervals)
☐	☒	For null hypothesis testing, the test statistic (e.g. *F*, *t*, *r*) with confidence intervals, effect sizes, degrees of freedom and *P* value noted<br>*Give P values as exact values whenever suitable.*
☒	☐	For Bayesian analysis, information on the choice of priors and Markov chain Monte Carlo settings
☒	☐	For hierarchical and complex designs, identification of the appropriate level for tests and full reporting of outcomes
☒	☐	Estimates of effect sizes (e.g. Cohen's *d*, Pearson's *r*), indicating how they were calculated

Our web collection on statistics for biologists contains articles on many of the points above.

Software and code

Policy information about availability of computer code

Data collection	Sample information (date of collection and Ct value) was stored in an Excel (version 16.48) spreadsheet and is available in Supplementary Data 4.
Data analysis	We extracted all the cut sites from the SARS-CoV-2 reference genome (NC_045512.2) using a custom Python script. Following this, we predicted the COVseq breadth of coverage by extending known cut site locations in the SARS-CoV-2 genome by the effective read length (theoretical sequence read length minus 20 bp of adapter sequence) on both sides.<br>We demultiplexed raw sequence reads to fastq files based on index sequences using the BaseSpace® Sequence Hub cloud service of Illumina. We then further demultiplexed individual libraries to fastq files for each sample using a custom Python script. Following this, we processed the samples using a Nextflow22 (version 20.10.0) based analysis pipeline from nf-core23 called viralrecon24 (version 1.1.0). In short, we trimmed the adapters from the fastq reads using fastp25 (version 0.20.1) and aligned them to the SARS-CoV-2 reference genome (NC_045512.2) using bowtie 226 (version 3.5.1). Following this, we sorted and indexed the reads using samtools27 (version 1.9), we trimmed amplicon primer sequences using ivar28 (version 1.2.2), called variants, and generated the subsequent consensus sequence also using ivar. To determine the percentage of reads that mapped to different organisms and common contaminants we used FastQ-screen29 (version 0.14.1). Briefly, 100,000 reads were sampled from the fastq files and aligned to 14 reference sequences using bowtie226 (version 3.5.1) (see Supplementary Table 2). We performed all subsequent analyses using custom R scripts.<br>We downloaded sequences and sequence metadata from GISAID (https://www.gisaid.org/ 2021-03-31) and added all the COVseq generated libraries and relevant metadata from the OAS-29, CCI-55, and OAS-95 samples. We then used the ncov tool (https://github.com/nextstrain/ncov) built on nextstrain21 to generate a temporal and spatial phylogenetic tree. Additionally, we randomly sampled 800 samples from around the world available in GISAID. We analyzed and visualized the resulting newick tree in R using ggtree30 (version 2.2.4).

For manuscripts utilizing custom algorithms or software that are central to the research but not yet described in published literature, software must be made available to editors and reviewers. We strongly encourage code deposition in a community repository (e.g. GitHub). See the Nature Research guidelines for submitting code & software for further information.

Data

Policy information about availability of data

All manuscripts must include a data availability statement. This statement should provide the following information, where applicable:

- Accession codes, unique identifiers, or web links for publicly available datasets
- A list of figures that have associated raw data
- A description of any restrictions on data availability

The BAM files used to generate all the plots in the main Figures and Supplementary Figures have been deposited to ENA and can be accessed at https://www.ebi.ac.uk/ena/browser/view/PRJEB42601. All reference sequences used in this study are listed in Supplementary Table 2. All the GISAID data used in this study are described in Supplementary Data 7.

All the custom code used for processing COVseq sequencing data and the custom MATLAB code used in the Cost Analysis (see Supplementary Notes) is available at https://github.com/ljwharbers/COVseq and the repository is linked to Zenodo: https://doi.org/10.5281/zenodo.4776499.

Field-specific reporting

Please select the one below that is the best fit for your research. If you are not sure, read the appropriate sections before making your selection.

[x] Life sciences [ ] Behavioural & social sciences [ ] Ecological, evolutionary & environmental sciences

For a reference copy of the document with all sections, see nature.com/documents/nr-reporting-summary-flat.pdf

Life sciences study design

All studies must disclose on these points even when the disclosure is negative.

Sample size	Sample sizes were chosen based on the number of available left-over RNA samples collected during Phase 1 (Mar-Apr 2020), Phase 2 (Oct-Nov 2020) and 3 (Feb-Mar 2021) of the pandemic at Ospedale Amedeo di Savoia (OAS) and Candiolo Cancer Institute (CCI) in Turin, Italy, respectively.
Data exclusions	We included in the manuscript all left-over RNA samples (274) that were retrospectively collected for this study. For specific analyses (e.g., variant calling), we only considered samples with low Ct value (<=35), which correspond to samples with the highest viral load and consequently high genome coverage.
Replication	We successfully assessed the reproducibility of our method by preparing four replicate libraries from the CCI-55 samples and three replicates from the OAS-95 samples. The results of these experiments are presented in Figures 2 and Supplementary Figure 3d-f, and are described in the manuscript. Briefly, as described in the manuscript, we observed high correlation between replicate samples with regards to genome coverage and SNV identification.
Randomization	Since we did not perform any statistical comparisons between distinct groups, randomization was not applicable.
Blinding	Since we did not perform any statistical comparisons between distinct groups, blinding was not applicable. However, during analysis we had no prior information on patient-specific PCR Ct values or on patient-specific SARS-CoV-2 lineage.

Reporting for specific materials, systems and methods

We require information from authors about some types of materials, experimental systems and methods used in many studies. Here, indicate whether each material, system or method listed is relevant to your study. If you are not sure if a list item applies to your research, read the appropriate section before selecting a response.

Materials & experimental systems

n/a	Involved in the study	
x		Antibodies
x		Eukaryotic cell lines
x		Palaeontology and archaeology
x		Animals and other organisms
	x	Human research participants
x		Clinical data
x		Dual use research of concern

Methods

n/a	Involved in the study	
x		ChIP-seq
x		Flow cytometry
x		MRI-based neuroimaging

Human research participants

Policy information about studies involving human research participants

Population characteristics	Part of the study was conducted on fully anonymous left-over RNA samples, collected from patients for which no population characteristics are known since no clinical or personal information was collected.
Recruitment	The samples were collected from COVID-19-positive individuals during the pandemic at the Ospedale Amedeo di Savoia hospital and the Candiolo Cancer Institute Turin, Italy. See Supplementary Data 4 for the date of collection.
Ethics oversight	The study was approved by the Ethical Committee of the Candiolo Cancer Institute (permit no. 57/2021) and by the Swedish Ethical Review Authority (permit no. 2020-06694).

Note that full information on the approval of the study protocol must also be provided in the manuscript.
