# Supplementary material for: COVseq is a cost-effective workflow for mass-scale SARS-CoV-2 genomic surveillance
Source: Nat Commun. 2021 Jun 23;12:3903. doi: 10.1038/s41467-021-24078-9 (PMC8222401; doi:10.1038/s41467-021-24078-9)
Supplement: Supplementary file 11 — Description of Additional Supplementary Files [file 41467_2021_24078_MOESM11_ESM.pdf]

Title: Supplementary Data 1.
Description: List of primers used in the CDC SARS-CoV-2 multiplexed PCR assay.

Title: Supplementary Data 2.
Description: List of oligonucleotides for preparing COVseq adapters.

Title: Supplementary Data 3.
Description: Summary of sequencing results.

Title: Supplementary Data 4.
Description: List of samples and corresponding Ct values.

Title: Supplementary Data 5.
Description: List of all SNVs identified and corresponding annotations by Pangolin. P-value is calculated with fisher's exact test.

Title: Supplementary Data 6.
Description: List of reagents and relative costs for sequencing SARS-CoV-2 samples using COVseq vs. three commercially available library preparation kits.

Title: Supplementary Data 7.
Description: Acknowledgement table of GISAID sequences used for the phylogenetic analysis shown in Fig. 3.
